# Inhibitors of the Elastase LasB for the Treatment of *Pseudomonas aeruginosa* Lung Infections

**DOI:** 10.1021/acscentsci.3c01102

**Published:** 2023-10-27

**Authors:** Jelena Konstantinović, Andreas M. Kany, Alaa Alhayek, Ahmed S. Abdelsamie, Asfandyar Sikandar, Katrin Voos, Yiwen Yao, Anastasia Andreas, Roya Shafiei, Brigitta Loretz, Esther Schönauer, Robert Bals, Hans Brandstetter, Rolf W. Hartmann, Christian Ducho, Claus-Michael Lehr, Christoph Beisswenger, Rolf Müller, Katharina Rox, Jörg Haupenthal, Anna K.H. Hirsch

**Affiliations:** † Helmholtz Institute for Pharmaceutical Research Saarland (HIPS)−Helmholtz Centre for Infection Research (HZI), Saarbrücken 66123, Germany; ‡ Department of Chemistry of Natural and Microbial Products, Institute of Pharmaceutical and Drug Industries Research, National Research Centre, El-Buhouth Street, Dokki, Cairo 12622, Egypt; § Department of Pharmacy, Pharmaceutical and Medicinal Chemistry, Saarland University, Saarbrücken 66123, Germany; ∥ Department of Internal Medicine V − Pulmonology, Allergology and Critical Care Medicine, Saarland University, Homburg 66421, Germany; ⊥ Saarland University, Department of Pharmacy, Saarbrücken 66123, Germany; # Department of Biosciences and Medical Biology, Division of Structural Biology, University of Salzburg, Salzburg 5020, Austria; ○ Helmholtz International Lab for Anti-infectives, Saarbrücken 66123, Germany; △ Department of Chemical Biology (CBIO), Helmholtz Centre for Infection Research (HZI), Braunschweig 38124, Germany; □ Deutsches Zentrum für Infektionsforschung (DZIF) e.V., Braunschweig 38124, Germany

## Abstract

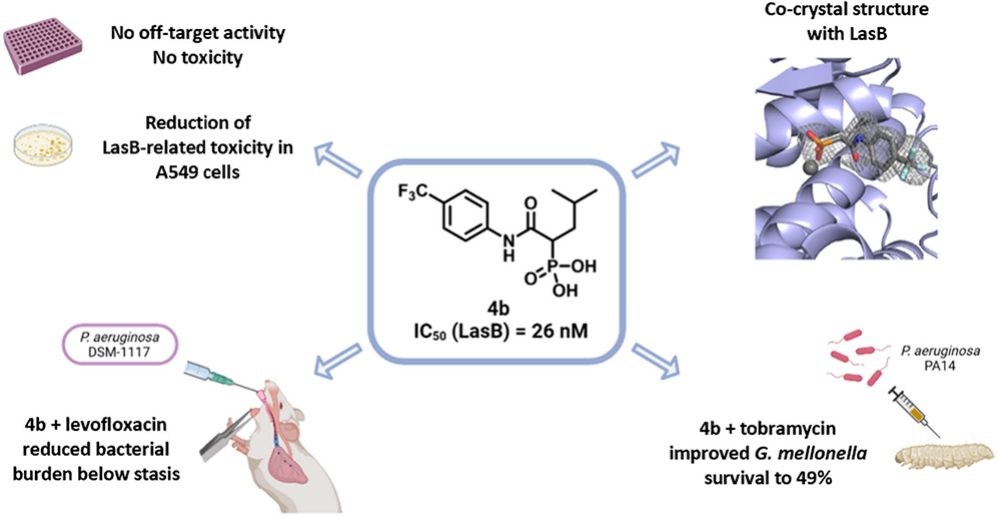

Infections caused by the Gram-negative pathogen *Pseudomonas aeruginosa* are emerging worldwide as a major threat to human health. Conventional antibiotic monotherapy suffers from rapid resistance development, underlining urgent need for novel treatment concepts. Here, we report on a nontraditional approach to combat *P. aeruginosa*-derived infections by targeting its main virulence factor, the elastase LasB. We discovered a new chemical class of phosphonates with an outstanding *in vitro* ADMET and PK profile, auspicious activity both *in vitro* and *in vivo*. We established the mode of action through a cocrystal structure of our lead compound with LasB and in several *in vitro* and *ex vivo* models. The proof of concept of a combination of our pathoblocker with levofloxacin in a murine neutropenic lung infection model and the reduction of LasB protein levels in blood as a proof of target engagement demonstrate the great potential for use as an adjunctive treatment of lung infections in humans.

## Abstract 2

New antivirulence agents targeting LasB demonstrate *in vivo* proof-of-concept and *in vivo* target engagement in *Pseudomonas aeruginosa* infections.

## Introduction

The silent pandemic of antimicrobial resistance is a global health threat, affecting millions of people worldwide. It is caused by natural mechanisms of pathogen defense, over- as well as misuse of antibiotics in humans as well as in animal husbandry. Consequently, even common infections are becoming increasingly problematic to treat. This leads to augmented costs in the healthcare sector including the necessity for longer treatments, and, despite that, still high mortality rates.^1−4^*Pseudomonas aeruginosa* is a Gram-negative bacterium which typically infects lungs, the urinary tract, and wounds, leading to severe infections challenging to treat due to drug-resistance.^5^ In 2017, the World Health Organization (WHO) published a priority list of pathogens raising the topic of an urgent need for novel antibiotics, in particular *P. aeruginosa*, identified as a top-three critical pathogen devoid of sufficient treatment options for drug-resistant strains.^6^*P. aeruginosa* is one of the major pathogens in cystic fibrosis (CF), as well as in noncystic fibrosis bronchiectasis (NCFB) patients, leading to chronic lung infection and poor pulmonary function.^7−10^ Furthermore, the pathogen plays important roles in hospital-acquired and ventilator-associated pneumonia, urinary-tract infections,^11^ keratitis,^12^ and wound infections.^13^ Taken together, there is an immediate necessity for the development of new anti-infectives–antibiotics with novel mechanisms of action or nontraditional approaches to fight antibiotic resistance, in particular, against Gram-negative pathogens.^14^

Recently, significant efforts have been put into the development of “pathoblockers”, agents capable of blocking bacterial virulence by disarming, rather than killing the pathogen. This should reduce the selection pressure and the formation of resistance.^15,16^ In particular, pathoblocker-antibiotic combinations are expected to have a synergistic effect and result in a more successful treatment.^17^*P. aeruginosa* secretes several virulence factors that serve as promising targets for the development of such pathoblockers.^18^ A central contributor to *P. aeruginosa* virulence is the elastase LasB, which plays a crucial role in the infection process. This extracellular proteolytic enzyme is responsible for tissue damage and has a destructive effect on various components of the immune system.^19^ These pathological roles strongly advise the development of drugs against *P. aeruginosa* by inactivating LasB, as recently thoroughly reviewed by Everett and Davies.^20^ In addition, one recent study came to the remarkable conclusion that elastase activity appears to be associated with 30-day mortality in intensive care unit patients.^21^ LasB or pseudolysin is a zinc-dependent metalloenzyme with an additional metal cation (Ca^2+^) as a cofactor in the active site.^22^ Therefore, the vast majority of the studied inhibitors contain zinc-binding groups (ZBGs), such as thiols,^23,24^ hydroxamates,^25^ carboxylic acids,^26^ tropolones,^27^ or 3-hydroxypyridine-4(1*H*)-thiones.^28^ We have extensively explored these ZBGs in the past few years and used those findings as the basis for the current work.^29−33^

Given the instability of thiols due to oxidation, we here present a systematic study of the 15 most common ZBGs as a suitable replacement for the thiol liability present in our inhibitors of LasB. Our ultimate goal is the identification of a stable, active, and safe pathoblocker to fill the dry antibiotic pipeline. In view of the envisioned application of these compounds as novel anti-infectives *in vivo*, the rationale of the most favorable ZBG was supported by *in vitro* absorption, distribution, metabolism, excretion, and toxicity (ADMET) profiling. Taken together, these data strongly favored the phosphonic acid derivatives as the most promising class. Pharmacokinetic studies in mice showed that these compounds have an excellent retention in lung tissue and epithelial lining fluid (ELF). In a pharmacodynamics study, the combination of the phosphonic acid inhibitor **4b** with levofloxacin reduced the bacterial burden below stasis in mice infected with *P. aeruginosa* DSM-1117. Additionally, a reduction of LasB protein in blood was observed, demonstrating target attainment of our pathoblockers.

## Results and Discussion

### Replacing the Thiol

**Figure 1:**
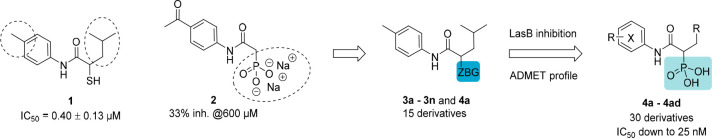
Optimization of **1** leading to phosphonate derivatives with nanomolar IC_50_ values against LasB (Table 1, Table S2–S4).

Previously, we reported on *N*-aryl-2-isobutylmercaptoacetamides showing submicromolar potencies against *P. aeruginosa* elastase LasB (thiol **1**, Figure 1).^29^ The approach to inhibit LasB (as a metalloprotease) comes with the intrinsic challenge of target selectivity over biochemically related human off-targets such as the human matrix-metalloproteases (MMPs). Despite being highly potent and selective over MMPs, the potential therapeutic application of *N*-aryl-2-isobutylmercaptoacetamides is limited due to the poor chemical stability of the free thiol group. Recently, we performed a screening of various ZBGs and investigated their effect on the activity against ColH, a collagenase secreted by *Clostridium histolyticum*.^34^ As this target is structurally and mechanistically closely related to LasB, we built on this knowledge and performed a similar screening on LasB. Phosphonate derivative **2** demonstrated modest activity toward LasB (Figure 1, Table S1). Our recent work on *N*-aryl-2-isobutylmercaptoacetamide **1**(29) suggested the importance of an alpha-alkyl substituent for the inhibition of LasB. Therefore, we designed 15 derivatives bearing an alpha-isobutyl side chain and different ZBGs, which yielded sulfonates, triazoles, hydroxamates, and phosphonates as possible alternatives to the thiol. Among them, the most potent turned out to be the hydroxamic acid derivative **3g** and the phosphonic acid **4a**. Subsequent exploration of their *in vitro* ADMET properties confirmed phosphonates to be the most promising class (Table 1, Table S2–S4). The detailed synthetic procedures of mentioned inhibitors have been described in Figures S1–S5.

**Table 1:**
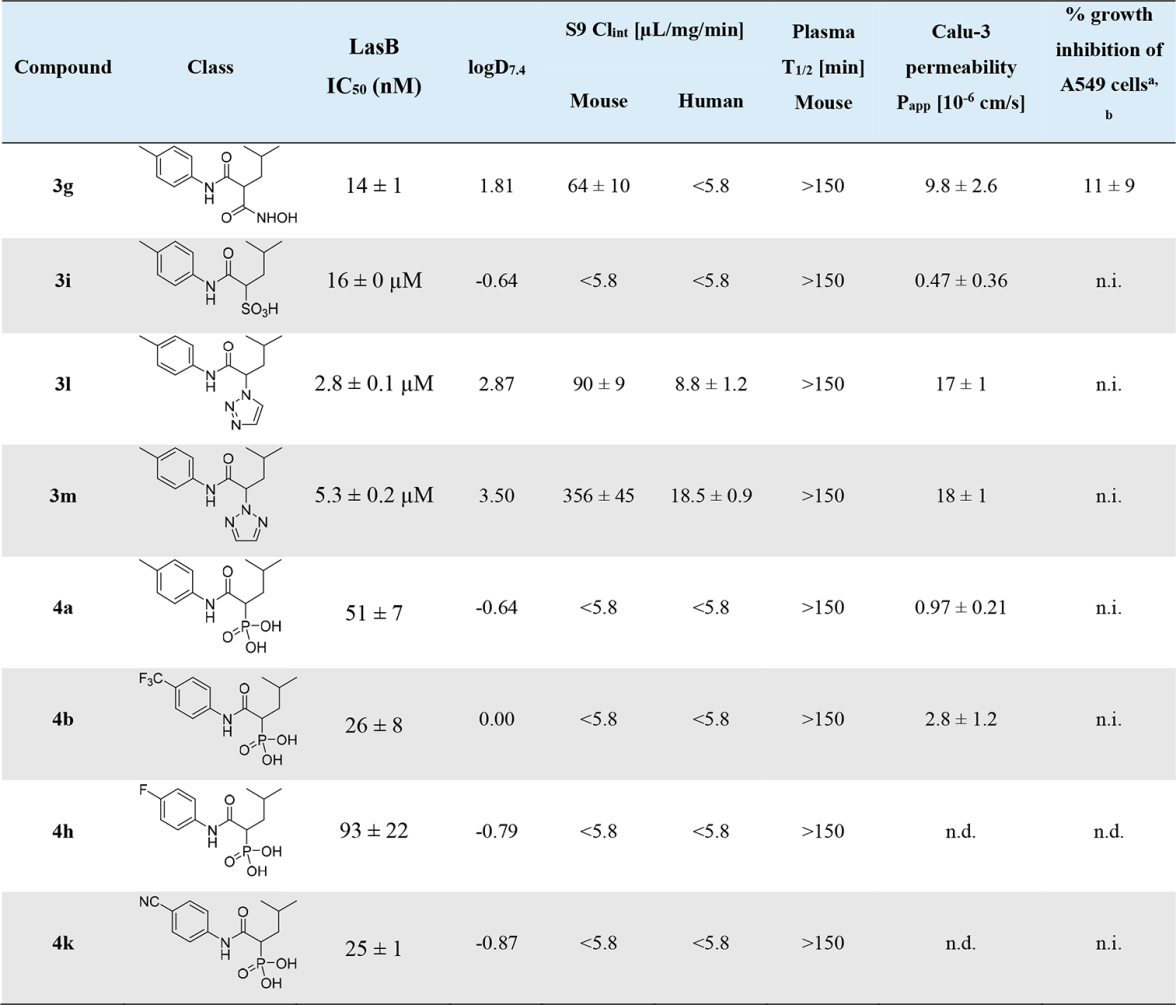
LasB Inhibition and Absorption, Distribution, Metabolism, Excretion, and Toxicity (ADMET) Profiling of Selected Compounds Bearing Different Zinc-Binding Groups (ZBGs)

a After 48 h at 100 μM.

b Further cytotoxicity data using cell lines HEK293 and HepG2 can be found in Table S6; n.d. = not determined; n.i. = < 10% inhibition; kinetic solubility was determined to be >200 μM for all tested compounds.

### LasB Inhibition by Compounds with Different ZBGs

We evaluated new derivatives for *in vitro* inhibition of LasB (Table S2) using a functional FRET-based assay, as established by Nishino et al.^35^ The corresponding thiol analogue **1** was used as comparator.^29^ Among 15 derivatives, five compounds showed IC_50_ values below 20 μM. The sulfonic acid derivative **3i** and two triazole derivatives **3l** and **3m** showed activity in the micromolar range, while the most pronounced activity was observed for the hydroxamic acid **3g** (IC_50_ = 14 ± 1 nM) and the phosphonic acid derivative **4a** (IC_50_ = 51 ± 7 nM). We improved activity by 30-fold and 8-fold, respectively, compared to our previous thiol hit **1**. As it has been shown previously that surfactant can impair the activity of drugs targeting the lung,^36^ we determined IC_50_ values in the presence of 1% pulmonary surfactant for our frontrunners. **4b** did only exhibit a 3-fold increase in IC_50_, resulting in potency in the nanomolar range (IC_50_ = 76 ± 23 nM).

#### Structure–Activity Relationships (SARs) of Phosphonic Acid Derivatives

The SARs were investigated by exploring three major modifications, including the substitution pattern of the aromatic core, replacement of the phenyl ring with 6-membered nitrogen heterocycles, and structural variations of the side chain, yielding in total a library of 30 phosphonic acid derivatives (Tables S3–S4). The most active derivative showed 16-fold improvement in the activity compared to our previous hit **1**, showing efficacy against LasB in the nanomolar range.

Among the first group of compounds bearing an α-isobutyl side-chain, we explored a different substitution pattern of the aromatic core, including electron-withdrawing and electron-donating substituents, both polar and lipophilic. From our previous work on α-substituted and nonsubstituted *N*-arylmercaptoacetamides,^29−32^ we knew that the para-position is more favorable for the activity compared to ortho and meta. Therefore, we explored the para-position with regard to the nature of the substituents introduced. All derivatives bearing electron-withdrawing substituents proved to be more active than those bearing electron-donating substituents. Among them, those with lipophilic substituents, such as 3,4-dichloro (**4c**) or 4-trifluoromethyl (**4b**), were 1.6-fold more active than those with polar electron-withdrawing substituent such as 4-acetyl (**4g**). Not only did the electronic properties influence the activity but the steric effects did as well, as illustrated by compounds **4h** (−F), **4i** (−Cl), and **4j** (−Br). These compounds all bear lipophilic substituents, only the former one bears fluorine, which turned out to be 2-fold less active than the other compounds bearing chlorine and bromine. The tolerability of sterically even more demanding substituents can be further seen in compounds with 1-naphthyl (**4m**), 2-naphthyl (**4n**), and 2,3-dihydro-1*H*-inden-5-yl (**4o**) as an aryl ring.

*Ortho*-substituents have a great impact on the conformation and electronic properties, thus indirectly influencing solubility. To explore the SAR around the aromatic core by not only changing the nature of the para-substituents, we synthesized 2-F-4-Me (**4d**) and 2,4-diMe derivatives (**4e**). Both of them demonstrated a ∼2-fold drop in activity compared to the 4-Me derivative (**4a**), most probably by changing the active conformation, **4d** through plausible hydrogen-bonding with the amide functional group and **4e** through steric effects.

To assess the importance of the α-isobutyl side-chain, we synthesized two derivatives with an α-benzyl (**4x** and **4y**), two with an α-methylcyclohexyl (**4z** and **4aa**), and three with an α-propyl side chain (**4ab–4ad**). In our previous work with thiol-containing compounds, we demonstrated that α-benzyl derivatives exhibit similar activities as α-isobutyl, while the methylcyclohexyl ones show up to a 30-fold drop in activity.^29−32^ To explore the α-substitution pattern in the new phosphonic acid-class, we have chosen 4-Me and 4-CF_3_ substituents on the left-hand side of the molecule, the former one being a direct comparison to **1**, while the latter being one of the most active representatives within the isobutyl class. With regard to the nature of the substituents on the aromatic ring, we noticed the same trend in all three classes: compounds bearing lipophilic trifluoromethyl substituent were more active than their methyl-analogues (**4b** vs **4a**, **4y** vs **4x**, and **4aa** vs **4z**). On the other hand, we observed that a benzyl side-chain led to a 3-fold drop in activity, while the methylcyclohexyl derivatives were active in the micromolar range. That the isobutyl side-chain is crucial for the activity is further confirmed with the series of three propyl derivatives (**4ab–4ad**), all three showing a micromolar potency, with a 20–80-fold drop in activity compared to their isobutyl analogs.

To further expand the SAR, we synthesized a small series of eight derivatives with 6-membered nitrogen-containing heterocycles, including pyridine, pyrimidine, and pyridazine, which might have an influence on the solubility and a metabolic profile (Table S4). In most of the compounds, we kept the 4-halo substituent, as we have shown that it is beneficial for activity. Unfortunately, all eight heterocyclic derivatives showed a drop in activity, from 8-fold (**4s**, IC_50_ = 0.19 μM) to 800-fold (**4v**, IC_50_ = 18.8 μM) compared to the most active compounds of the isobutyl series (IC_50_ ∼ 25 nM). That the position of the nitrogen has an impact on the activity is seen from a comparison of the 5-chloropyridin-2-yl- (**4p**) and 6-chloropyridin-3-yl- (**4q**) derivative, with the former one being 2-fold more potent. Surprisingly, substituents such as carboxamide and carboxymethyl reversed the activity profile of derivatives containing a nitrogen atom in the meta-position. Among pyrimidine and pyridazine derivatives, bromo-substituents proved more beneficial for activity compared to chloro-substituents, while in cases of both halogens, pyrimidine derivatives (**4t** and **4u**) were significantly more active than the pyridazine (**4v** and **4w**).

### Crystal Structure of **4b**

**Figure 2:**
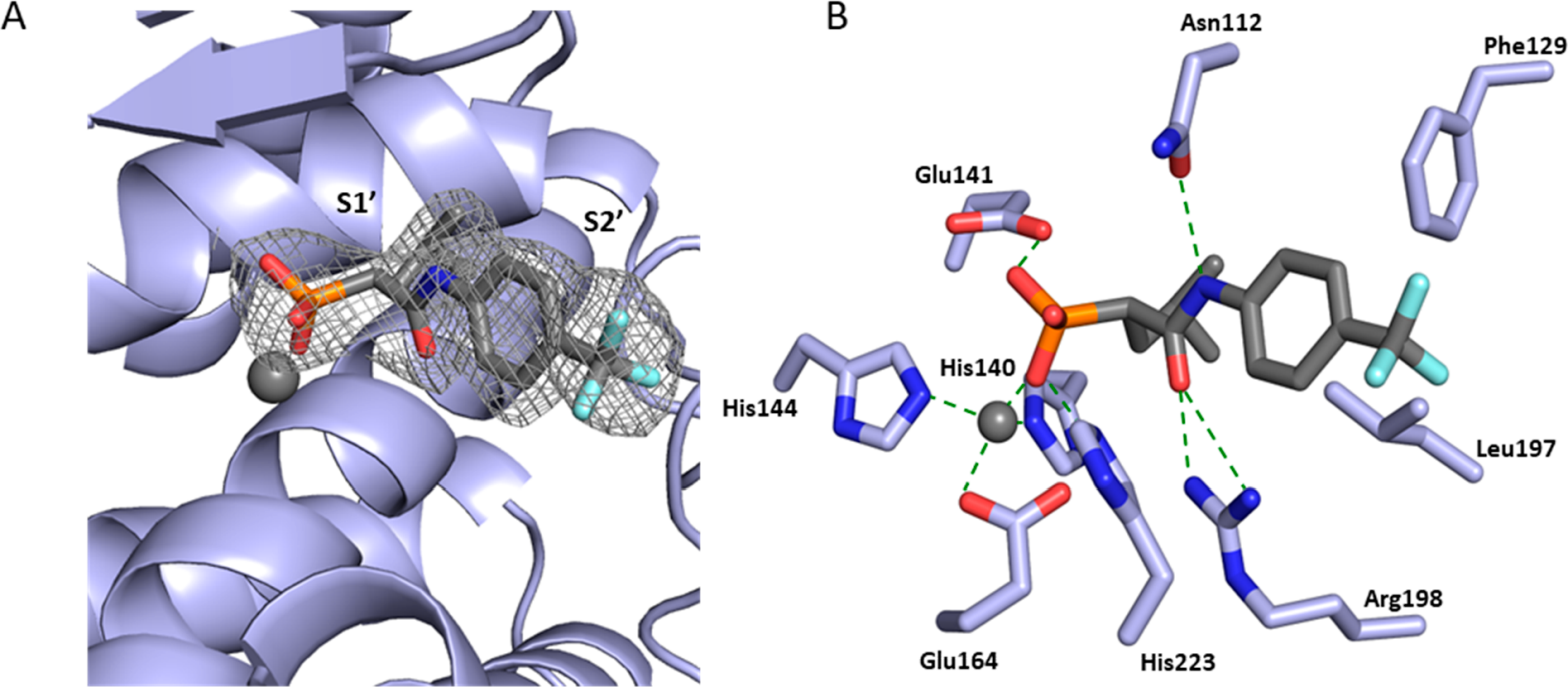
Crystal structure of LasB in complex with **4b** (PDB code: 8CC4). (**A**) Cartoon representation of LasB (slate) in complex with **4b** (gray), with the S1′ and S2′ binding-sites of the enzyme occupied by the compound highlighted. The gray isomesh represents a polder map of **4b** contoured at 3 σ. (**B**) Schematic 2D representation of LasB-**4b** interactions. Hydrogen bonds are displayed in dotted green lines, while all other residues exhibit hydrophobic interactions with the ligand. The active site Zn^2+^ cation is shown as a gray sphere.

To elucidate the binding mode of phosphonic acid derivatives, we cocrystallized **4b** with LasB (Figure 2A). Full details of the data collection and refinement statistics can be found in Table S13. The compound binds in a fashion that is similar to a previously reported α-substituted mercaptoacetamide derivative (PDB code: 7OC7)^31^ and occupies the S1′-S2′ pockets with the phosphonate group coordinating the active site Zn^2+^ cation (Figure S13A). In addition, **4b** also forms a number of additional H bonds and hydrophobic interactions that can be seen on Figure 2B. This explains the significant improvement in activity over the mercaptoacetamide class. The carbonyl oxygen of **4b** forms a bidentate hydrogen bond with Arg198, while the side chains of His223, Glu141, and Asn112 form hydrogen bonds with the phosphonate and amide groups. The aryl group occupies the wide, open, and solvent-accessible entrance of the lipophilic S2′ binding pocket, which rationalizes the tolerance for substitution at this position (Figure S13B). The replacement of the isobutyl group by bulkier substituents is detrimental, likely due to the steric constraints of the S1′ pocket that cannot accommodate such large groups without a steric clash (Figure S13A).

### ADMET Profiling

To select the most suitable ZBG for our LasB inhibitors, we considered their *in vitro* ADMET profile along with their *in vitro* potency (Table 1). For this purpose, we compared hydroxamic acid derivative **3g**, sulfonic acid **3i**, and the two triazoles **3l** and **3m**, with the phosphonic acids **4a**, **4b**, **4h**, and **4k**. A key decision criterion was permeability across Calu-3 monolayers, which constitutes an important feature of antipseudomonal drugs targeting the lung. Since our overall goal was to achieve good lung retention after pulmonary administration, low permeability was desirable to avoid rapid dissemination of the LasB inhibitor into systemic circulation. Sulfonic acid **3i** (*P*_app_ = 0.47 × 10^–6^ cm/s) and phosphonic acids **4a** and **4b** (*P*_app_ = 0.97 × 10^–6^ cm/s and 2.8 × 10^–6^ cm/s, respectively) showed the lowest *P*_app_ values. Additionally, we investigated kinetic solubility and lipophilicity (log *D*_7.4_), murine and human metabolic stability, as well as murine plasma stability (Table 1). The results suggest that the phosphonic acid derivatives have the best overall ADMET profile, i.e., high solubility, low lung permeability, and high stability in mouse and human liver S9 fractions and mouse plasma. Further, we profiled three selected phosphonates regarding their metabolism in different species, confirming excellent metabolic and plasma stability in rat and minipig (Table S5).

To rationalize the observed striking differences in cell permeability, we correlated the measured permeabilities *P*_app_ with chromatographic lipophilicities log *D*_7.4_ (Figure S6). Notably, there is a very good correlation between compound lipophilicity and Calu-3 permeability for our set of LasB inhibitors (*R*^2^ = 0.9865). Namely, hydroxamate **3g** as well as triazoles **3l** and **3m** give significantly higher log *D*_7.4_ values (>1.8) compared to the phosphonates (log *D*_7.4_ ≤ 0). This correlation strengthens our selection of phosphonates as the most promising ZBG, given that their high hydrophilicity and negative charge at physiological pH reduces the permeability across the more lipophilic cell layer, hence favoring lung retention. Furthermore, this good correlation can potentially be applied in the future as a useful tool to rationally design compounds with physicochemical properties favorable for good lung exposure.

Representative derivatives were tested for their cytotoxicity on three human cell-lines: HepG2 (hepatocellular carcinoma), HEK293 (embryonal kidney), and A549 (lung carcinoma). All measured growth inhibition values were less than 30% at 100 μM, thus all compounds showed an excellent profile and did not bear cytotoxicity potential at concentrations relevant for *in vivo* assessment (Table 1, Table S6).

### Biological Evaluation of Phosphonic Acids in Target Validation Models

To assess the activity of the phosphonic acid class in target–validation models, we tested selected compounds in two different cell-based assays including lung organoids, constituting an increasingly complex matrix close to the physiological setting, as well as in a simple in vivo model based on *Galeria mellonella* larvae.

#### Evaluation of LasB Inhibitors in A549 Cells Treated with *P. aeruginosa* PAO1 and PA14 Supernatants

First, we challenged the human lung adenocarcinoma cell line A549 with culture supernatants (csn) derived from wild-type *P. aeruginosa* PAO1 or PA14 (wt PAO1; wt PA14) as well as lasB knockout strains (PAO1 ΔlasB; PA14 ΔlasB) to assess the inhibitory effect of two phosphonate LasB inhibitors, **4a** and **4b**, on the proteolytic, elastolytic, and cytotoxic properties. Both compounds demonstrated an excellent dose-dependent reduction of LasB-related cytotoxicity in csn-treated cells, comparable to the levels of the knockout strain. This illustrates impressively the relevance of LasB and its inhibition (Figure S7A–B). Interestingly, higher concentrations of **4b** were necessary to achieve similar effects against PA14 csn, which could be attributed to the higher amounts of secreted LasB by PA14 (Figure S7D).

**Figure 3:**
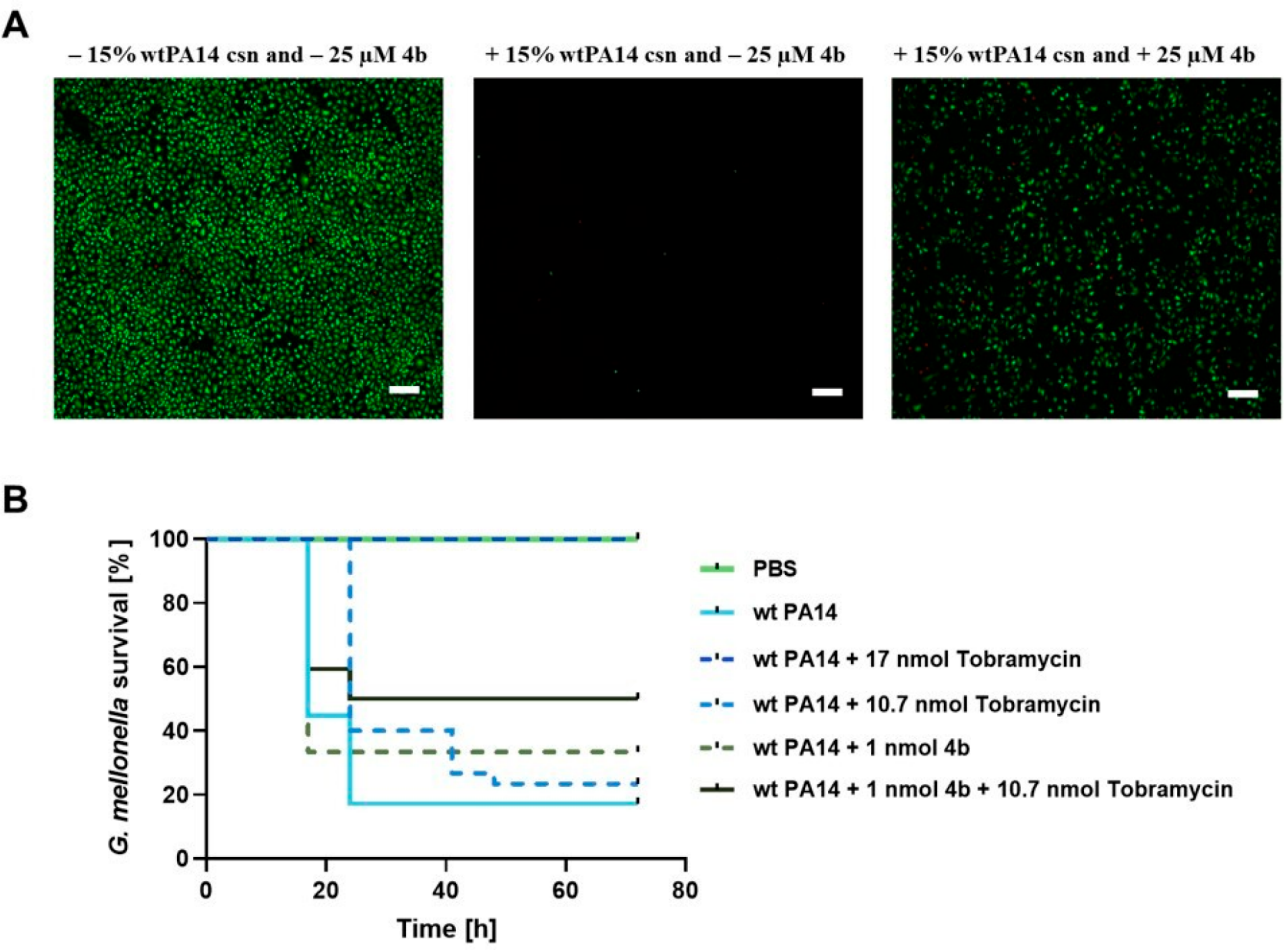
(A) **4b** maintained the viability of lung (A549) cells upon treatment with 15 wt % PA14 *csn*. Live/dead imaging with A549 cells challenged with 15% (*v/v*) of PA14 csn with and without **4b**. Green signals: living cells; red signals: dead cells. Red signals in some cases were lost because the detached cells were washed away after the rinsing step with PBS. Scale bar: 200 μm for images. (B) Probability of survival of the *Galleria mellonella* larvae infected with wt PA14: The survival of larvae infected with wt PA14 is shown after treatment with 1 nmol **4b**, 10.7 nmol tobramycin, or a combination of both. The survival in the PBS-**4b** or tobramycin or combination of both treated groups was 100%. The statistical difference between groups treated with 1 nmol **4b** + 10.7 nmol tobramycin and treated with only wt PA14 is *p* = 0.0005 (log-rank). Each curve represents results of three independent experiments. wt PA14: wild-type PA14; ΔPA14: LasB knockout PA14; and csn: culture supernatant.

Compound **4b** sustained viability of cells, even at low concentrations (Figure 3A, Figure S8).^29,32,37^ LasB targets the extracellular matrix component collagen and leads to its degradation. **4b** reduced cleavage of collagen by 20% at a concentration of 3.15 μM (Figure S7C).^20,38,39^ To exclude that effects of **4b** might be attributed to other proteases in PA14 csn, we deployed PA14 *ΔlasB* csn. No effect on the viability and on collagen was observed using the Δ*lasB* csn (Figure S7B–C). This demonstrates that **4b** selectively inhibited LasB and did not affect other proteases in the csn.

#### Lung Organoid Assay

In the past years, lung organoids have been extensively used to study pulmonary diseases due to the high resemblance of their structural features and their functions with the native lung.^40,41^ We studied the effect of PA14 csn on the viability of 3D lung organoids and the rescue effect by inhibitors **4a** and **4b** (Figure S9). In this experiment, primary human bronchial epithelial cells (HBECs) were differentiated to human 3D bronchospheres^42^ and challenged with 5% PA14 csn. LasB inhibitors **4a** and **4b** did not have significant toxic effects on the nontreated lung organoids in concentrations tested up to 100 μM. Upon treatment with PA14 csn, viability of the organoids was reduced to 30–60%. By contrast, treatment with LasB inhibitors **4a** and **4b** resulted in a strong beneficial effect as viability of the organoids was improved in a dose-dependent manner (Figure S9). Therefore, effectiveness of LasB inhibitors was also demonstrated in the 3D model constituting a complex matrix system close to the physiological situation.

#### Effect of 4b in a *G. mellonella* Infection Model

Next, we infected *G. mellonella* larvae, a standard model for evaluation of novel anti-infectives,^43^ with wt PA14 and treated them with **4b** and tobramycin (individually and in combination), a standard-of-care antibiotic in CF.^44^ For the combination experiments, we chose a lower tobramycin dose (10.7 nmol, corresponding to 0.5 μg/mL in the dosed solution), aiming at the improvement of a subefficacious dose of tobramycin with **4b**. A higher tobramycin dose of 17 nmol (0.8 μg/mL) was used as 100% efficacious dose of the antibiotic. While treatment with **4b** or tobramycin at a low dose alone only led to a slight increase in survival (33% and 23%, respectively, compared to 17% in the wild type), the combination of **4b** and tobramycin significantly improved the survival to 49% (Figure 3B).

Importantly, **4b** as well as further selected phosphonates do not possess any antibacterial activity up to 100 μM (Figure S10C). In line with this, the combination with tobramycin did not increase the antibacterial activity of the antibiotic alone *in vitro* (Figure S10A-B), indicating that the observed improvement of *in vivo* activity was caused by the antivirulence effect of the LasB inhibitor. Hence, this study reinforced the potential of our compound to boost the efficacy of antibiotics *in vivo* by targeting the virulence factor LasB.

### Selectivity for LasB over Human off-Targets and Other Bacterial Proteases As Well As Advanced off-Target Safety Screening and Zebrafish Embryo Toxicity

Next, we aimed to test selectivity against human off-targets and other bacterial proteases and safety. We assessed four most potent inhibitors (**4a**, **4b**, **4k**, and **4l)** which showed a significantly improved selectivity profile against human off-targets compared to our previously published hit, compound **1** (Table S7). **4b** and **4k** had slight effects at 100 μM against MMP-1, -2, and -3 (with shallow, intermediate, and deep binding pockets, respectively), which was negligible when compared to their activity toward LasB (IC_50_ ∼ 25 nM). On the contrary, the hydroxamic acid **3g**, although being 2-fold more potent compared to the best phosphonate inhibitor, exhibited slight effects on MMP-1, -2, and -3 and inhibition of tumor necrosis factor-alpha converting enzyme (TACE) in the micromolar range (Table S7). In contrast, the phosphonic acids did not inhibit TACE, HDAC-3, HDAC-8, and COX-1 (Table S7). We previously reported on a structural similarity between inhibitors of *P. aeruginosa* elastase LasB and *C. histolyticum* collagenase ColH.^30,33,34^ Most of our newly developed inhibitors demonstrated no or only weak inhibition of ColH-PD (**4a**, **4b**, **4c**, **4d**, and **4f**), with the notable exception of **4g**. This compound was a more potent ColH inhibitor than the previous “best-in-class” phosphonate inhibitor of ColH, i.e., **1** (see Supporting Information). It might therefore be developed toward a dual inhibitor of both metalloprotease virulence factors LasB and ColH if a medical need for such an agent with dual activity arises (Table S8).

Then, we assessed potential additional safety liabilities of **4a** and **4b** using the Eurofins SafetyScreen44 panel to identify the most important off-target interactions at an early stage. When tested at a concentration of 10 μM, compound **4a** demonstrated an excellent safety profile with no significant inhibition of any of the mentioned targets (Figure S11). Furthermore, to gain a deeper insight into the potential clinical applicability, we investigated the toxicity of compounds *in vivo* on zebrafish embryos. Compounds **4a** and **4b** demonstrated a maximum tolerated concentration (MTC) of ≥100 μM (Table S9). In summary, **4a** and **4b** demonstrated an excellent safety profile qualifying for additional *in vivo* preclinical testing.

### Pharmacokinetic Studies in Mice

**Figure 4:**
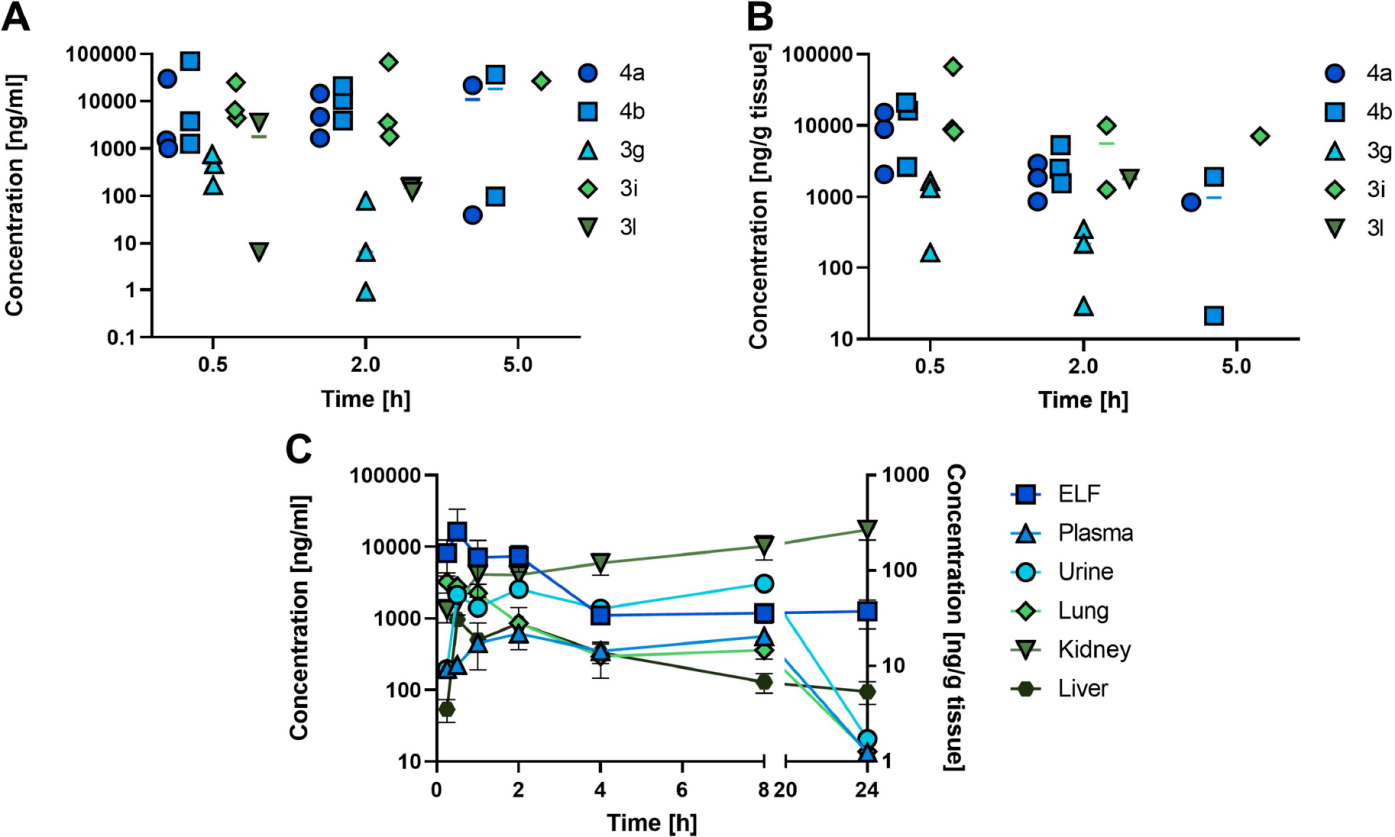
(A) Concentrations of five selected ZBGs in ELF and (B) lung tissue after IT administration at 0.25 mg/kg (cassette dosing). (C) Concentrations of **4b** after nebulization of 10 mg/kg in ELF, plasma, urine, lung tissue, kidney tissue, and liver tissue.

To further assess the potential of the new inhibitors for the treatment of lung infections, we subjected five selected compounds (**3g**, **3i**, **3l**, **4a**, and **4b**) to pharmacokinetic studies in mice as a cassette dosing via intratracheal (IT) administration. The selection comprised representatives bearing different ZBGs in order to assess whether the observed differences in their *in vitro* permeabilities also resulted in different lung retentions *in vivo*. Hydroxamic acid **3g** and triazole derivative **3l** were found in ELF and lung tissue at significantly lower concentrations compared to sulfonic acid **3i** and phosphonic acids **4a** and **4b**. Moreover, hydroxamic acid **3g** was not detected 2 h after administration, which is perfectly in line with the observed differences in Calu-3 cell permeation. Quite remarkably, the concentrations of both phosphonic acids **4a** and **4b** were significantly above the IC_50_ values determined in the FRET-assay in the presence of surfactant (Figure 4A,B, Table S10). The concentration levels of the five selected compounds in BALF, plasma, urine, kidney, and liver tissue are summarized in Figure S12.

Following IT cassette dosing, we performed a more focused PK study with compound **4b** at a single dose of 10 mg/kg, administered by nebulization (Figure 4C). Selection of this compound for further *in vivo* analysis was based on the highest levels reached in ELF. Samples were analyzed for a period of 24 h. The compound rapidly appeared systemically and was detected in plasma, urine, ELF, lung tissue, kidney, and liver already after 15 min (Figure 4B). Still, the compound had high initial ELF levels with a *C*_max_ of about 20 μg/mL (∼770-fold relative to the IC_50_ value in the presence of surfactant) and remaining in a moderate range until 24 h (Table S11). Lung tissue concentrations followed similar kinetics, although the concentration levels dropped significantly after 8 h. **4b** appeared rapidly in plasma with a *T*_max_ of about 4 h and a *C*_max_ of about 633 ng/mL (Figure 4B, Table S12). Nevertheless, **4b** exhibited only a moderate to low fractionated plasma clearance (Table S12). Furthermore, low compound levels were observed in the liver tissue, whereas kidney tissue showed increasing levels. In line with the observed absence of liver metabolism *in vitro* and high concentrations found in urine, these findings suggest primarily renal clearance of the unmodified inhibitors.

### Pharmacodynamic Studies in Mice Infected with *P. aeruginosa* DSM-1117

**Figure 5:**
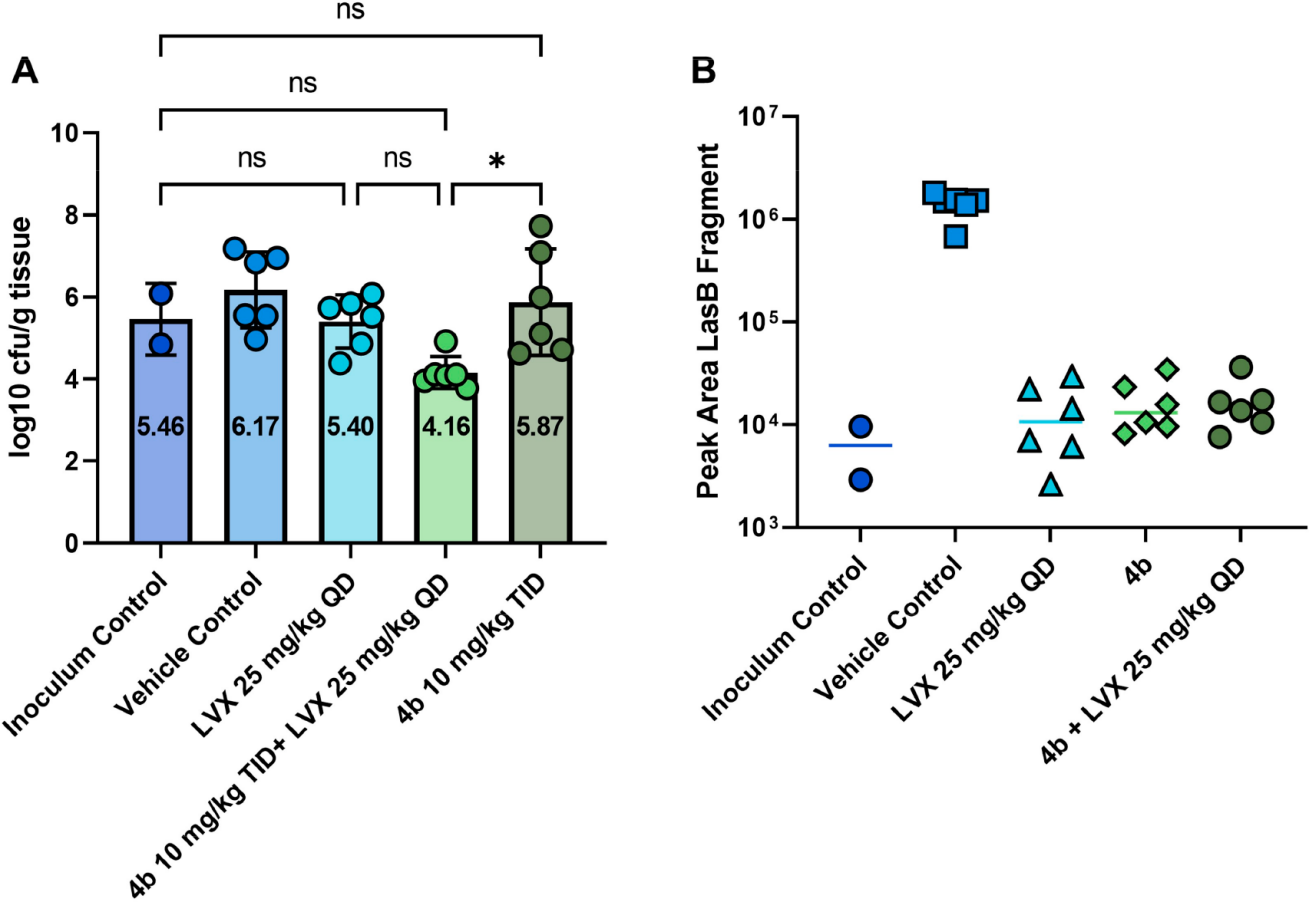
(A) Bacterial growth in lung tissue given as log 10 cfu/g tissue. Mice in all groups were treated with *P. aeruginosa* DSM-1117. Inoculum control, the number of colony-forming units (cfu) in the lungs was determined 15 min after infection; all other lungs were taken after 24 h; vehicle control: mice treated with a vehicle; LVX 25 mg/kg: mice treated with levofloxacin; **4b** 10 mg/kg TID (3 times per day) + LVX 25 mg/kg: mice treated with a combination of **4b** and levofloxacin; **4b** 10 mg/kg TID: mice treated with **4b**. (B) LasB levels in blood in mice infected with *P. aeruginosa* DSM-1117.

Encouraged by the results of **4b** in several *in vitro* and *ex vivo* assays, its efficacy in the *in vivo* infection model in *G. mellonella*, as well as the excellent lung and ELF retention after IT administration (0.25 mg/kg, cassette dosing) and nebulization (10 mg/kg), we performed a pharmacodynamic (PD) study using a neutropenic lung infection model with *P. aeruginosa* DSM-1117. In this experiment, we evaluated the effect of the antibiotic levofloxacin at 25 mg/kg, **4b** at 10 mg/kg TID, and a combination of both on the number of CFU in the lungs of the mice. The bacteria and **4b** were applied by nebulization, whereas levofloxacin was administered IP. Figure 5A shows the bacterial growth in lung tissue. A slight growth of bacteria was observed in the vehicle control group compared to the inoculum control. The levofloxacin group reduced the bacterial burden back to stasis, while **4b** alone did not show an effect on the number of CFU. For a pathoblocker reducing virulence of a bacterium rather than killing it, this is not necessarily to be expected, especially in a neutropenic model. Adjunctive therapy is further considered a valid strategy in the development of antivirulence agents.^14,45−48^ Remarkably, the combination of **4b** and levofloxacin showed a clear synergistic effect, significantly reducing the bacterial burden below stasis. Moreover, assessment of LasB protein levels in blood as an indicator of dissemination showed a clear reduction for levofloxacin and the combination group compared to the vehicle group and back to the level of the inoculum control group. Remarkably, a strong reduction was also observed for the **4b** only group, although no effect on CFU had been detected in lung tissue. This reduction in the presence of the LasB inhibitor, **4b**, and in absence of levofloxacin provided the proof of concept and target engagement (Figure 5B) and allows speculation about a possibly, highly desirable, lower bacterial dissemination into the body.

## Conclusions

Targeting bacterial virulence is a compelling approach as it provides a milder selective pressure for the emergence of resistance compared to conventional antibiotics, which are killing the bacteria or preventing their growth. Given its extracellular location, LasB is considered a particularly attractive target in the current antimicrobial resistance crisis. Inhibitors targeting this enzyme are circumventing the permeability challenge as they do not need to cross the Gram-negative cell wall of *P. aeruginosa* to demonstrate activity. A systematic exploration of ZBGs furnished a new class of phosphonic acid inhibitors showing nanomolar potency toward LasB and an excellent *in vitro* ADMET profile. We demonstrated that the compounds with the lowest permeability in the Calu-3 assay are at the same time showing the highest levels in ELF and a good lung retention with concentrations several magnitudes above IC_50_ values in the presence of surfactant. This correlation of Calu-3 *in vitro* and PK *in vivo* data supports the use of Calu-3 assays as a predictive tool for the selection of compounds for *in vivo* studies, thereby increasing the success rate of these animal models while at the same time reducing the number of animals needed for testing. In the *G. mellonella* infection model, compound **4b** in combination with tobramycin demonstrated a significant improvement in survival which was translated into efficacy in a murine neutropenic lung infection model with *P. aeruginosa* DSM-1117, where the same compound in combination with levofloxacin reduced the bacterial burden significantly below stasis. These results highlight the potential of LasB inhibitors to be combined with different classes of antibiotics. Further *in vivo* proof of concept and target engagement was demonstrated by significantly reduced LasB levels in the blood even when **4b** was used as monotherapy. Without affecting bacterial growth while at the same time clearly demonstrating strong antivirulence effects against LasB *in vitro* and in several target-validation models, the potential of our inhibitors as a new class to be used in a combination therapy with standard-of-care (SOC) antibiotics for the treatment of lung infections in humans is supported. In conclusion, our study provides novel pathoblockers targeting LasB with high potency, excellent safety, and selectivity profile and demonstrated *in vivo* proof of concept as well as proof of target engagement, perfectly suited for further development via inhalative administration to enrich treatment options of infections in CF and NCFB patients.^49^ The results described above represent an important milestone for the development of anti-infective agents targeting LasB. To the best of our knowledge, here we demonstrated for the first time a significant increase of the SOC antibiotics’ efficacy in an adjunctive therapy with a LasB inhibitor. Our future work will focus on further optimization of the presented inhibitors with regard to their potency, pharmacokinetic properties, and *in vivo* efficacy.

## Data Availability

All data are available in the main text or the Supporting Information.

## Supporting Information Available

Experimental procedures for the synthesis of all compounds; NMR spectra of compounds **3g**, **3i**, **3l**, **3m**, **4a**, **4b**, **4h**, and **4k**; materials and methods; and supplementary text: synthesis of derivatives bearing different ZBGs, ADMET profiling, selectivity against human off-targets, activity against *Clostridium histolyticum* collagenase ColH, off-target safety screen44 panel, and zebrafish embryo toxicity; and Figures S1–S13, Tables S1–S13, and references (50–74) (PDF)
