# Supplementary material for: Inhibitors of the Elastase LasB for the Treatment of Pseudomonas aeruginosa Lung Infections
Source: ACS Cent Sci. 2023 Oct 27;9(12):2205–15. doi: 10.1021/acscentsci.3c01102 (PMC10755728; doi:10.1021/acscentsci.3c01102)
Supplement: Supplementary file 1 — oc3c01102_si_001.pdf [file oc3c01102_si_001.pdf]

Supporting Information for

Inhibitors of the Elastase LasB for the treatment of *Pseudomonas aeruginosa* lung infections

Jelena Konstantinović, Andreas M. Kany, Alaa Alhayek, Ahmed S. Abdelsamie, Asfandyar Sikandar, Katrin Voos, Yiwen Yao, Anastasia Andreas, Roya Shafiei, Brigitta Loretz, Esther Schönauer, Robert Bals, Hans Brandstetter, Rolf W. Hartmann, Christian Ducho, Claus-Michael Lehr, Christoph Beisswenger, Rolf Müller, Katharina Rox, Jörg Haupenthal, Anna K.H. Hirsch.

Correspondence to: anna.hirsch@helmholtz-hips.de

This PDF file includes:

Experimental section
Materials and Methods
Supplementary Text
Figures S1 to S13
Tables S1 to S13

Experimental section

Chemistry. All reagents were used from commercial suppliers without further purification. Procedures were not optimized regarding yield. NMR spectra were recorded on a Bruker AV 500 (500 MHz) spectrometer. Chemical shifts are given in parts per million (ppm) and referenced against the residual proton, ^{1}H, or carbon, ^{13}C, resonances of the >99% deuterated solvents as internal reference. Coupling constants (J) are given in Hertz. Data are reported as follows: chemical shift, multiplicity, coupling constants, and integration. Liquid chromatography-mass spectrometry was performed on a LC-MS system, consisting of a Dionex UltiMate 3000 pump, autosampler, column compartment and detector (Thermo Fisher Scientific, Dreieich, Germany) and ESI quadrupole MS (MSQ Plus or ISQ EC, Thermo Fisher Scientific, Dreieich, Germany). High resolution mass was determined by LC-MS/MS using Thermo Scientific Q Exactive Focus Orbitrap LC-MS/MS system. Purity of the final compounds was determined by LC-MS using the area percentage method on the UV trace recorded at a wavelength of 254 nm and found to be >95%.

General procedure A-1: Amide coupling to afford derivatives 3a, S3, S5a–S5q, S5t, S10a–S10d and S13a–S13c

The acid (1.2–2.0 eq) was dissolved in DCM. EDC·HCl (1.2–2.0 eq) was added, followed by the corresponding aniline (1.0 eq). The resultant mixture was stirred at room temperature, until the starting aniline was consumed. The obtained solution was washed with 1 M HCl and brine. The organic layer was dried over anhydrous sodium sulfate, filtered and concentrated under reduced pressure to afford the crude product. The obtained crude product was either used in the next step without further purification or purified using column chromatography.

General procedure A-2: Amide coupling to afford derivatives S5r–S5s and S5u

A mixture of 2-bromo-4-methylpentanoic acid (1.0 eq), thionyl chloride (10.0 eq) and DMF (5 drops) in THF was heated at 50 °C for 3 hours. The reaction mixture was cooled to room temperature, the solvent and the excess of thionyl chloride were removed under reduced pressure. The crude 2-bromo-4-methylpentanoyl chloride in THF was added at 0 °C under N_2 atmosphere to the corresponding aniline (1 eq). After 30 minutes at 0 °C, the ice bath was removed and the solution was warmed up and stirred at 50 °C for 2 hours. The reaction mixture was cooled to room temperature, the solvent was removed under reduced pressure. The reaction mixture was extracted

with CH_2Cl_2, the organic layer was dried over $MgSO_4$, filtered and the solution was concentrated under reduced pressure to afford the crude product. The obtained crude product was either used in the next step without further purification or purified using column chromatography.

General procedure B: Synthesis of imidazole and triazole derivatives 3k and 3m–3n

2-Bromo-4-methyl-*N*-(*p*-tolyl)pentanamide **S5a** (1.0 equiv.) was placed in a crimp vial and dissolved in acetone. Corresponding imidazole or triazole (1.1 equiv.) and K_2CO_3 (1.1. equiv.) were added and the mixture heated to 70 °C overnight (or otherwise specified). EtOAc was added, the organic layer washed with water and saturated aqueous NaCl solution, dried over anhydrous Na_2SO_4, filtered and concentrated under reduced pressure. The crude was purified by preparative HPLC.

General procedure C: Synthesis of diethyl phosphonate derivatives S7a–S7u, S11a–S11d and S14a–S14c

N-Aryl-2-halo-2-alkylacetamide derivative (1.0 eq) was suspended in triethyl phosphite (10 eq) and heated to 150 °C in a sealed tube for a total of 18 h (or otherwise specified). Most of unreacted triethyl phosphite was evaporated in vacuo and the resultant oil was purified by column chromatography.

General procedure D: Synthesis of phosphonic acid derivatives 4a–4ad

To a solution of diethyl phosphonate (1.0 eq) in dry DCM, bromotrimethylsilane (5.0–7.0 eq) was added dropwise over a period of 15 min. The reaction mixture was stirred at r.t. overnight (or otherwise specified). If no full conversion was achieved, the excess of bromotrimethylsilane (5.0 eq) was added next day. Then MeOH was added and stirred for 30 min at room temperature to cleave the previously formed TMS ester. Solvents were concentrated in vacuo and the resultant oil was purified by preparative HPLC.

2-Chloro-3-phenylpropanoic acid **S9a** and 2-chloro-3-cyclohexylpropanoic acid **S9b** were synthesized according to the procedure we described previously.[31]

***Tert*-butyl (*S*)-(4-methyl-1-oxo-1-(*p*-tolylamino)pentan-2-yl)carbamate (3a).**

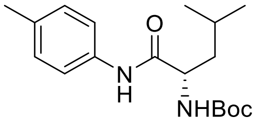

Compound **3a** was synthesized according to general procedure A-1, using *p*-toluidine (193 mg, 1.80 mmol), (*tert*-butoxycarbonyl)-L-leucine (500 mg, 2.16 mmol) and EDC·HCl (414 mg, 2.16 mmol) in DCM (30 mL). The

reaction was stirred at room temperature for 3 h. The crude product was purified using column chromatography (Hex/EtOAc=9/1 to 8/2). The product was obtained as white solid (525 mg, 91%). ^{1}H NMR (500 MHz, DMSO-d_6) δ ppm: 9.86 (s, 1H), 7.47 (d, *J* = 8.2 Hz, 2H), 7.09 (d, *J* = 8.4 Hz, 2H), 7.02 (d, *J* = 8.1 Hz, 1H), 4.12–4.06 (m, 1H), 2.24 (s, 3H), 1.68–1.57 (m, 1H), 1.55–1.46 (m, 1H), 1.43–1.39 (m, 1H), 1.37 (s, 9H), 0.90–0.86 (m, 6H). ^{13}C NMR (126 MHz, DMSO-d_6) δ ppm: 171.66, 155.54, 136.63, 132.18, 129.17, 119.29, 78.07, 53.53, 40.76, 28.29, 24.43, 23.06, 21.65, 20.54. MS (ESI$^+$) m/z 221.16 [M-Boc+H]$^+$.

(*S*)-2-amino-4-methyl-*N*-(*p*-tolyl)pentanamide (3b).

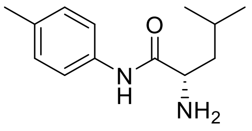

Compound **3a** (50 mg, 0.16 mmol) was dissolved in DCM (2 mL) and TFA (120 µL, 1.6 mmol) was added. The reaction was stirred at room temperature overnight. Solvents were evaporated, fresh DCM added and washed with 2 M NaOH and brine. The product was obtained as beige solid (17 mg, 49%). ^{1}H NMR (500 MHz, $CDCl_3$) δ ppm: 9.42 (br s, 1H), 7.49 (d, *J* = 8.4 Hz, 2H), 7.13 (d, *J* = 8.4 Hz, 2H), 3.51 (dd, *J* = 10.0, 3.7 Hz, 1H), 2.32 (s, 3H), 1.86–1.74 (m, 2H), 1.47–1.39 (m, 1H), 1.00 (d, *J* = 6.4 Hz, 3H), 0.98 (d, *J* = 6.3 Hz, 3H). ^{13}C NMR (126 MHz, $CDCl_3$) δ ppm: 173.61, 135.54, 133.60, 129.59, 119.45, 54.05, 44.06, 25.16, 23.59, 21.47, 21.01.

2-Hydroxy-4-methyl-*N*-(*p*-tolyl)pentanamide (S3).

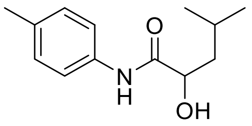

Compound **S3** was synthesized according to general procedure A-1, using *p*-toluidine (184 mg, 1.7 mmol), 2-hydroxy-4-methylpentanoic acid (343 mg, 2.6 mmol) and EDC·HCl (502 mg, 2.6 mmol) in DCM (20 mL). The reaction was stirred at room temperature for 18 h. The crude product obtained after the workup was used in the next step without further purification.

4-Methyl-1-oxo-1-(*p*-tolylamino)pentan-2-yl sulfamate (3c).

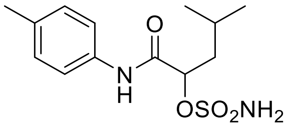

Compound **S3** (100 mg, 0.45 mmol) was dissolved in *N*,*N*-dimethylacetamide (0.6 mL) under Ar atmosphere and cooled in an ice-bath. Sulfamoyl chloride (208 mg, 1.8 mmol) was added, the reaction was stirred at 0 °C for 10 minutes and then at room temperature overnight. EtOAc was added and washed with water and brine. The crude was purified by preparative HPLC (CH_3CN (HCOOH 0.05%)-H_2O (HCOOH 0.05%): 1.0:9.0 to 10.0:0.0). The product **3c** was obtained as white solid (57 mg, 42%). ^{1}H NMR (500 MHz, DMSO-d_6) δ ppm: 9.91 (s, 1H), 7.68 (s, 2H), 7.50 (d, *J* = 8.5 Hz, 2H), 7.11 (d, *J* = 8.4 Hz, 2H), 4.83–4.77 (m, 1H), 2.25 (s, 3H), 1.81–1.71 (m, 2H), 1.63–1.51

(m, 1H), 0.96–0.92 (m, 6H). ^{13}C NMR (126 MHz, DMSO-d_6) δ ppm: 167.63, 135.94, 132.67, 129.01, 119.81, 77.31, 41.10, 23.70, 22.88, 21.68, 20.46. HRMS (ESI$^+$) calculated for $C_{13}H_{21}N_2O_4S$ [M+H]$^+$ 301.1217, found 301.1215.

Ethyl 4-methyl-2-(*p*-tolylcarbamoyl)pentanoate (3d).

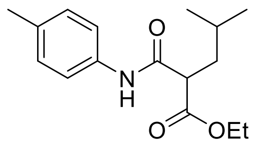

Diethyl 2-ethylmalonate (645 mg, 2.98 mmol) was dissolved in EtOH/H_2O (30 mL, 4:1) and NaOH (143 mg, 3.58 mmol) was added. The reaction was stirred at r.t. overnight. EtOH was evaporated under reduced pressure, saturated aqueous $NaHCO_3$ solution was added and extracted with DCM. The organic layer was discarded. The aqueous layer was acidified with 6 M HCl and extracted with DCM. The organic layer was washed with brine, dried over anhydrous Na_2SO_4, filtered and evaporated under reduced pressure. In the second step, the obtained mono-acid **S4** (505 mg, 2.68 mmol) and EDC·HCl (515 mg, 2.68 mmol) were added to a solution of *p*-toluidine (240 mg, 2.23 mmol) in DCM (20 mL). The resultant mixture was stirred at rt overnight. The mixture was washed with 1 M HCl and saturated aqueous NaCl solution. After the workup, the obtained crude product was purified using column chromatography (Hex/EtOAc=8/2). The product was obtained as orange crystals (441 mg, 71%). ^{1}H NMR (500 MHz, $CDCl_3$) δ ppm: 8.45 (br s, 1H), 7.42 (d, J = 8.1 Hz, 2H), 7.13 (d, J = 8.1 Hz, 2H), 4.31–4.17 (m, 2H), 3.43 (t, J = 7.7 Hz, 1H), 2.32 (s, 3H), 1.94–1.80 (m, 2H), 1.69–1.61 (m, 1H), 1.35–1.28 (m, 3H), 0.96 (d, J = 6.6 Hz, 6H). ^{13}C NMR (126 MHz, $CDCl_3$) δ ppm: 173.33, 166.58, 135.18, 134.19, 129.60, 119.99, 61.88, 52.53, 40.98, 26.53, 22.62, 22.16, 21.02, 14.23. HRMS (ESI$^+$) calculated for $C_{16}H_{24}NO_3$ [M+H]$^+$ 278.1751, found 278.1747.

4-Methyl-2-(*p*-tolylcarbamoyl)pentanoic acid (3e).

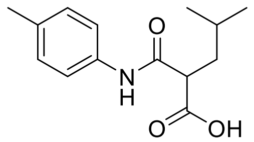

Compound **3d** (100 mg, 0.36 mmol) was dissolved in EtOH (5 mL) and NaOH (28.8 mg, 0.72 mmol) in water (1.2 mL) was added. The reaction was stirred at r.t. overnight. The mixture was acidified with 1 M HCl and extracted with EtOAc. The organic layer was washed with 1M HCl and brine, dried over anhydrous Na_2SO_4, filtered and evaporated under reduced pressure. The product was obtained as beige solid (87.3 mg, 97%). ^{1}H NMR (500 MHz, DMSO-d_6) δ ppm: 12.63 (br s, 1H), 10.09 (s, 1H), 7.47 (d, J = 8.5 Hz, 2H), 7.10 (d, J = 8.2 Hz, 2H), 3.47–3.43 (m, 1H), 2.24 (s, 3H), 1.76–1.68 (m, 1H), 1.64–1.57 (m, 1H), 1.53–1.43 (m, 1H), 0.89 (d, J = 6.6 Hz, 3H), 0.87 (d, J = 6.6 Hz, 3H). ^{13}C NMR (126 MHz, DMSO-d_6) δ ppm: 171.56, 167.45, 136.57, 132.42, 129.20, 119.22, 51.04, 37.46, 25.73, 22.85, 22.02, 20.53. HRMS (ESI$^+$) calculated for $C_{14}H_{20}NO_3$ [M+H]$^+$ 250.1438, found 250.1434.

2-Isobutyl-*N*1-(*p*-tolyl)malonamide (3f).

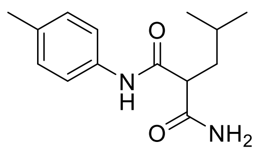

Compound **3d** (50 mg, 0.18 mmol) was dissolved in MeOH (6 mL) and ammonia (3 mL, 25% aq) was added. The reaction was stirred at r.t. overnight. The solvents were evaporated under reduced pressure and the crude was purified by preparative HPLC (CH_3CN (HCOOH 0.05%)-H_2O (HCOOH 0.05%): 1.0:9.0 to 10.0:0.0) to obtain compound **3f** as white solid (23.5 mg, 52%). ^{1}H NMR (500 MHz, DMSO-d_6) δ ppm: 9.86 (s, 1H), 7.46 (d, *J* = 8.4 Hz, 2H), 7.25 (s, 1H), 7.15 (s, 1H), 7.10 (d, *J* = 8.4 Hz, 2H), 3.29 (t, *J* = 7.4 Hz, 1H), 2.24 (s, 3H), 1.72–1.59 (m, 2H), 1.53–1.43 (m, 1H), 0.88 (d, *J* = 6.4 Hz, 3H), 0.87 (d, *J* = 6.6 Hz, 3H). ^{13}C NMR (126 MHz, DMSO-d_6) δ ppm: 171.39, 168.40, 136.44, 132.44, 129.19, 119.35, 52.59, 38.79, 25.91, 22.57, 22.34, 20.54. HRMS (ESI$^+$) calculated for $C_{14}H_{21}N_2O_2$ $[M+H]^+$ 249.1598, found 249.1595.

***N*^{1}-hydroxy-2-isobutyl-*N*^{3}-(*p*-tolyl)malonamide (3g).**

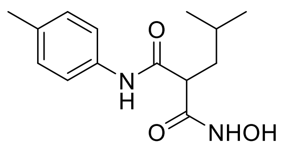

Ethyl 4-methyl-2-(*p*-tolylcarbamoyl)pentanoate **3d** (100 mg, 0.36 mmol) was dissolved in MeOH (2 mL). NH_2OH 50 wt % in H_2O (2 mL) and KCN (4.7 mg, 0.07 mmol) were added and the mixture was stirred at rt overnight. Solvents were concentrated in vacuo and the resultant oil was purified by preparative HPLC (CH_3CN (HCOOH 0.05%)-H_2O (HCOOH 0.05%): 1.0:9.0 to 10.0:0.0). The product was obtained as white solid (49 mg, 52%). ^{1}H NMR (500 MHz, DMSO-d_6) δ ppm: 10.54 (s, 1H), 9.66 (s, 1H), 9.00 (s, 1H), 7.45 (d, *J* = 8.4 Hz, 2H), 7.10 (d, *J* = 8.4 Hz, 2H), 3.18 (t, *J* = 7.6 Hz, 1H), 2.24 (s, 3H), 1.67 (t, *J* = 7.2 Hz, 2H), 1.47 (dquin, *J* = 13.4, 6.7, 6.7, 6.7, 6.7 Hz, 1H), 0.87 (br d, *J* = 6.6 Hz, 3H), 0.87 (br d, *J* = 6.6 Hz, 3H). ^{13}C NMR (126 MHz, DMSO-d_6) δ ppm: 167.58, 166.49, 136.36, 132.45, 129.17, 119.42, 49.96, 38.12, 25.78, 22.52, 22.32, 20.53. HRMS (ESI$^+$) calculated for $C_{14}H_{21}N_2O_3$ $[M+H]^+$ 265.1547, found 265.1545.

2-Bromo-4-methyl-*N*-(*p*-tolyl)pentanamide (S5a).

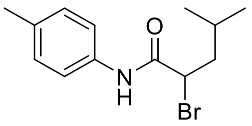

Compound **S5a** was synthesized according to general procedure A-1, using *p*-toluidine (92 mg, 0.85 mmol), 2-bromo-4-methylpentanoic acid **S6** (200 mg, 1.02 mmol) and EDC·HCl (197 mg, 1.02 mmol) in DCM (15 mL). The reaction was stirred at room temperature for 5 h. After the extraction, the product was obtained as beige solid (242 mg, quant. yield) and used in the next step without further purification.

2-Cyano-4-methyl-*N*-(*p*-tolyl)pentanamide (3h).

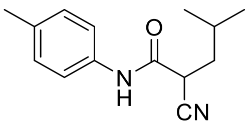

NaCN (51.7 mg, 1.06 mmol) was dissolved in water/DMF mixture (0.15 mL/0.5 mL). Compound **S5a** (150 mg, 0.53 mmol) was dissolved in DMF (1 mL) and added dropwise to the mixture. The reaction was stirred at r.t. for 4 days. Et_2O was added, the organic layer washed brine, dried over anhydrous Na_2SO_4, filtered and concentrated under reduced pressure. The crude was purified by preparative HPLC (CH_3CN (HCOOH 0.05%)-H_2O (HCOOH 0.05%): 1.0:9.0 to 10.0:0.0) to give product **3h** (92 mg, 76%) as white solid. ^1H NMR (500 MHz, DMSO-d_6) δ ppm: 10.32 (s, 1H), 7.44 (d, *J* = 8.4 Hz, 2H), 7.14 (d, *J* = 8.2 Hz, 2H), 3.88 (dd, *J* = 9.1, 6.5 Hz, 1H), 2.26 (s, 3H), 1.84–1.65 (m, 3H), 0.95 (d, *J* = 6.4 Hz, 3H), 0.92 (d, *J* = 6.4 Hz, 3H). ^{13}C NMR (126 MHz, DMSO-d_6) δ ppm: 163.78, 135.74, 133.29, 129.39, 119.55, 118.58, 38.35, 37.16, 26.08, 22.36, 21.45, 20.54. HRMS (ESI$^+$) calculated for $C_{14}H_{19}N_2O$ $[M+H]^+$ 231.1492, found 231.1490.

4-Methyl-1-oxo-1-(*p*-tolylamino)pentane-2-sulfonic acid (3i).

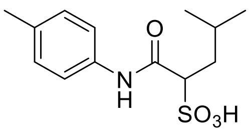

Compound **S5a** (50 mg, 0.18 mmol) was dissolved in EtOH (1 mL) and added dropwise to the solution of Na_2SO_3 (33.3 mg, 0.26 mmol) in water (1 mL). The mixture is stirred at 80 °C overnight. The solvents were evaporated and crude purified by preparative HPLC (CH_3CN (HCOOH 0.05%)-H_2O (HCOOH 0.05%): 1.0:9.0 to 10.0:0.0) to give product **3i** (21.9 mg, 44%) as pale orange oil. ^1H NMR (500 MHz, DMSO-d_6) δ ppm: 9.75 (s, 1H), 7.46 (d, *J* = 8.4 Hz, 2H), 7.07 (d, *J* = 8.4 Hz, 2H), 3.50–3.46 (m, 1H), 2.23 (s, 3H), 1.91 (ddd, *J* = 13.2, 11.0, 4.6 Hz, 1H), 1.60–1.44 (m, 2H), 0.84 ppm (d, *J* = 6.4 Hz, 6H). ^{13}C NMR (126 MHz, DMSO-d_6) δ ppm: 167.24, 137.17, 131.58, 129.03, 118.80, 65.34, 37.56, 25.86, 23.42, 21.71, 20.52. HRMS (ESI$^-$) calculated for $C_{13}H_{18}NO_4S$ $[M-H]^-$ 284.0962, found 284.0964.

4-Methyl-2-(2*H*-tetrazol-5-yl)-*N*-(*p*-tolyl)pentanamide (3j).

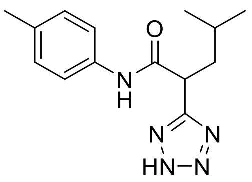

Suspension of **3h** (92 mg, 0.40 mmol), $ZnBr_2$ (98.8 mg, 0.44 mmol) and NaN_3 (39 mg, 0.60 mmol) in iPrOH/water mixture (0.5 mL/1.5 mL) in a sealted tube was stirred at 130 °C overnight. Sticky orange solid precipitated at the bottom of the tube. Solvents were removed and solid washed with EtOAc and water. Dissolved in methanol and purified by preparative HPLC (CH_3CN (HCOOH 0.05%)-H_2O (HCOOH 0.05%): 1.0:9.0 to 10.0:0.0) to give product **3j** (15.4 mg, 14%) as beige solid. ^1H NMR (500 MHz, DMSO-d_6) δ ppm: 10.41 (s, 1H), 7.48–7.44 (m, 2H), 7.12 (d, *J* = 8.4

Hz, 2H), 4.36 (t, *J* = 7.9 Hz, 1H), 2.24 (s, 3H), 2.01–1.94 (m, 1H), 1.88–1.81 (m, 1H), 1.41–1.31 (m, 1H), 0.92–0.88 (m, 6H). ^{13}C NMR (126 MHz, DMSO-d_6) δ ppm: 167.90, 154.98, 136.04, 132.88, 129.21, 119.45, 41.17, 25.70, 22.13, 22.04, 20.46. HRMS (ESI$^+$) calculated for $C_{14}H_{20}N_5O$ [M+H]$^+$ 274.1662, found 274.1655.

2-(1*H*-imidazol-1-yl)-4-methyl-*N*-(*p*-tolyl)pentanamide (3k).

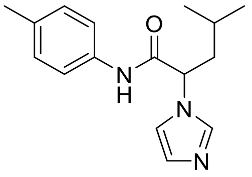

Compound **3k** was synthesized according to general procedure B, using **S5a** (70 mg, 0.25 mmol), acetone (7 mL), 1*H*-imidazole (18.4 mg, 0.27 mmol) and K_2CO_3 (37.4 mg, 0.27 mmol). The mixture was stirred at 70 °C for 4 days. The crude was purified by preparative HPLC (CH_3CN (HCOOH 0.05%)-H_2O (HCOOH 0.05%): 1.0:9.0 to 10.0:0.0) to give product **3k** (15 mg, 22%) as white solid. ^{1}H NMR (500 MHz, DMSO-d_6) δ ppm: 10.27 (s, 1H), 7.73 (s, 1H), 7.45 (d, *J* = 8.4 Hz, 2H), 7.26 (s, 1H), 7.12 (d, *J* = 8.2 Hz, 2H), 6.91 (s, 1H), 5.00 (dd, *J* = 9.2, 6.6 Hz, 1H), 2.25 (s, 3H), 1.98–1.86 (m, 2H), 1.33–1.23 (m, 1H), 0.91 (d, *J* = 6.7 Hz, 3H), 0.89 (d, *J* = 6.7 Hz, 3H). ^{13}C NMR (126 MHz, DMSO-d_6) δ ppm: 167.70, 136.63, 135.85, 132.91, 129.23, 128.12, 119.48, 118.53, 58.46, 40.70, 24.40, 22.47, 21.63, 20.45. HRMS (ESI$^+$) calculated for $C_{16}H_{22}N_3O$ [M+H]$^+$ 272.1757, found 272.1754.

4-Methyl-*N*-(*p*-tolyl)-2-(1*H*-1,2,3-triazol-1-yl)pentanamide (3l) and 4-methyl-*N*-(*p*-tolyl)-2-(2*H*-1,2,3-triazol-2-yl)pentanamide (3m).

4-Methyl-*N*-(*p*-tolyl)-2-(1*H*-1,2,3-triazol-1-yl)pentanamide (**3l**) and 4-methyl-*N*-(*p*-tolyl)-2-(2*H*-1,2,3-triazol-2-yl)pentanamide (**3m**) were synthesized according to general procedure B, using **S5a** (70 mg, 0.25 mmol), acetone (7 mL), 1*H*-1,2,3-triazole (18.7 mg, 0.27 mmol) and K_2CO_3 (37.4 mg, 0.27 mmol). The crude product was purified by preparative HPLC (CH_3CN (HCOOH 0.05%)-H_2O (HCOOH 0.05%): 1.0:9.0 to 10.0:0.0), giving products **3l** (20.3 mg, 30%) and **3m** (30 mg, 45%) as white solids.

4-Methyl-*N*-(*p*-tolyl)-2-(1*H*-1,2,3-triazol-1-yl)pentanamide (3l).

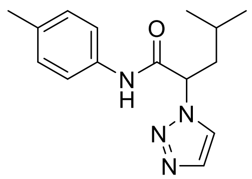

^{1}H NMR (500 MHz, DMSO-d_6) δ ppm: 10.52 (s, 1H), 8.30 (d, *J* = 0.8 Hz, 1H), 7.77 (d, *J* = 0.6 Hz, 1H), 7.46 (d, *J* = 8.4 Hz, 2H), 7.13 (d, *J* = 8.2 Hz, 2H), 5.61 (dd, *J* = 9.8, 6.1 Hz, 1H), 2.25 (s, 3H), 2.16–2.06 (m, 1H), 2.01–1.93 (m, 1H), 1.31–1.20 (m, 1H), 0.92 (d, *J* = 6.8 Hz, 3H), 0.90 (d, *J* = 6.6 Hz, 3H). ^{13}C NMR (126 MHz, DMSO-d_6) δ ppm: 166.59, 135.70, 133.36, 133.13, 129.28, 123.91,

119.53, 61.57, 40.45, 24.50, 22.38, 21.46, 20.46. HRMS (ESI$^+$) calculated for $C_{15}H_{21}N_4O$ [M+H]$^+$ 273.1710, found 273.1708.

4-Methyl-*N*-(*p*-tolyl)-2-(2*H*-1,2,3-triazol-2-yl)pentanamide (3m).

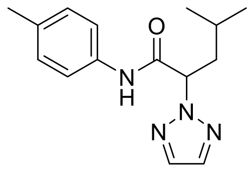

^{1}H NMR (500 MHz, DMSO-d_6) δ ppm: 10.35 (s, 1H), 7.83 (s, 2H), 7.45 (d, *J* = 8.4 Hz, 2H), 7.11 (d, *J* = 8.4 Hz, 2H), 5.46 (dd, *J* = 9.3, 6.0 Hz, 1H), 2.34–2.28 (m, 1H), 2.24 (s, 3H), 1.99 (ddd, *J* = 13.8, 7.8, 6.2 Hz, 1H), 1.47–1.36 (m, 1H), 0.91 (t, *J* = 6.2 Hz, 6H). ^{13}C NMR (126 MHz, DMSO-d_6) δ ppm: 166.18, 135.91, 134.49, 132.87, 129.21, 119.46, 65.81, 24.52, 22.50, 21.70, 20.46. HRMS (ESI$^+$) calculated for $C_{15}H_{21}N_4O$ [M+H]$^+$ 273.1710, found 273.1708.

4-Methyl-*N*-(*p*-tolyl)-2-(1*H*-1,2,4-triazol-1-yl)pentanamide (3n).

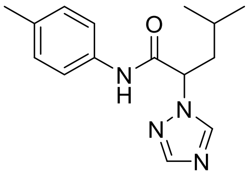

Compound **3n** was synthesized according to general procedure B, using **S5a** (100 mg, 0.35 mmol), acetone (10 mL), 1*H*-1,2,4-triazole (26.7 mg, 0.39 mmol) and K_2CO_3 (53.5 mg, 0.39 mmol). The mixture was stirred at 70 °C for 3 days. The crude was purified by preparative HPLC (CH_3CN (HCOOH 0.05%)-H_2O (HCOOH 0.05%): 1.0:9.0 to 10.0:0.0) to give product **3n** (52.8 mg, 55%) as white solid. ^{1}H NMR (500 MHz, DMSO-d_6) δ ppm: 10.41 (s, 1H), 8.72 (s, 1H), 7.98 (s, 1H), 7.45 (d, *J* = 8.5 Hz, 2H), 7.12 (d, *J* = 8.4 Hz, 2H), 5.26 (dd, *J* = 9.6, 6.3 Hz, 1H), 2.24 (s, 3H), 2.17–2.09 (m, 1H), 1.99–1.91 (m, 1H), 1.39–1.30 (m, 1H), 0.94–0.88 (m, 6H). ^{13}C NMR (126 MHz, DMSO-d_6) δ ppm: 166.98, 150.97, 143.69, 135.84, 133.11, 129.35, 119.57, 61.84, 24.60, 22.47, 21.67, 20.54. HRMS (ESI$^+$) calculated for $C_{15}H_{21}N_4O$ [M+H]$^+$ 273.1710, found 273.1708.

2-Bromo-4-methyl-*N*-(4-(trifluoromethyl)phenyl)pentanamide (S5b).

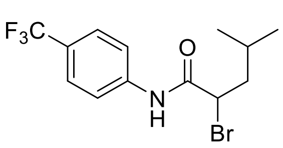

Compound **S5b** was synthesized according to general procedure A-1, using 4-(trifluoromethyl)aniline (125 µL, 0.98 mmol), 2-bromo-4-methylpentanoic acid **S6** (230 mg, 1.18 mmol) and EDC·HCl (226 mg, 1.18 mmol) in DCM (20 mL). The reaction was stirred at room temperature for 48 h. The crude product was purified using column chromatography (Hex/EtOAc=9/1 to 8/2). The product was obtained as white solid (193 mg, 58%). ^{1}H NMR (500 MHz, $CDCl_3$) δ ppm: 8.14 (br s, 1H), 7.68 (d, *J* = 8.5 Hz, 2H), 7.62 (d, *J* = 8.7 Hz, 2H), 4.47 (dd, *J* = 9.5, 5.3 Hz, 1H), 2.10–1.97 (m, 2H), 1.96–1.88 (m, 1H), 1.02 (d, *J* = 6.7 Hz, 3H), 0.97 (d, *J* = 6.6 Hz, 3H). ^{13}C NMR (126 MHz, $CDCl_3$) δ ppm: 167.32, 140.23, 126.76 (q, J_{C-F} = 34 Hz), 126.43–126.30 (m), 123.94 (q, J_{C-F} = 273 Hz), 119.52, 50.33, 44.42, 26.41, 22.64, 21.01. MS (ESI$^+$) m/z 338.04 [M+H]$^+$.

2-Bromo-*N*-(3,4-dichlorophenyl)-4-methylpentanamide (S5c).

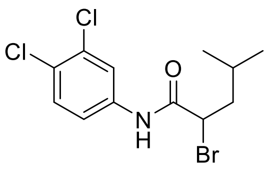

Compound **S5c** was synthesized according to general procedure A-1, using 3,4-dichloroaniline (208 mg, 1.28 mmol), 2-bromo-4-methylpentanoic acid **S6** (300 mg, 1.54 mmol) and EDC·HCl (295 mg, 1.54 mmol) in DCM (20 mL). The reaction was stirred at room temperature overnight. The crude product was purified using column chromatography (Hex/EtOAc=9/1 to 8/2). The product was obtained as white solid (293 mg, 67%). ^{1}H NMR (500 MHz, DMSO-d_6) δ ppm: 10.69 (s, 1H), 7.99 (d, J = 2.4 Hz, 1H), 7.60 (d, J = 8.7 Hz, 1H), 7.48 (dd, J = 8.8, 2.4 Hz, 1H), 4.57 (t, J = 7.6 Hz, 1H), 1.95–1.81 (m, 2H), 1.70–1.60 (m, 1H), 0.93 (d, J = 6.7 Hz, 3H), 0.88 (d, J = 6.6 Hz, 3H). ^{13}C NMR (126 MHz, DMSO-d_6) δ ppm: 167.57, 138.55, 131.22, 130.96, 125.49, 120.52, 119.42, 48.01, 42.49, 26.10, 22.21, 21.56.

2-Bromo-*N*-(2-fluoro-4-methylphenyl)-4-methylpentanamide (S5d).

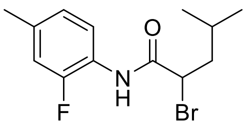

Compound **S5d** was synthesized according to general procedure A-1, using 2-fluoro-4-methylaniline (120 µL, 1.07 mmol), 2-bromo-4-methylpentanoic acid **S6** (250 mg, 1.28 mmol) and EDC·HCl (246 mg, 1.28 mmol) in DCM (20 mL). The reaction was stirred at room temperature overnight. The crude product was purified using column chromatography (Hex/EtOAc=9/1 to 8/2). The product was obtained as pale-yellow solid (244 mg, 76%). ^{1}H NMR (500 MHz, $CDCl_3$) δ ppm: 8.15 (br s, 1H), 8.10 (t, J = 8.5 Hz, 1H), 7.00–6.90 (m, 2H), 4.46 (dd, J = 9.4, 5.6 Hz, 1H), 2.33 (s, 3H), 2.10–1.97 (m, 2H), 1.96–1.89 (m, 1H), 1.01 (d, J = 6.7 Hz, 3H), 0.97 (d, J = 6.6 Hz, 3H). ^{13}C NMR (126 MHz, $CDCl_3$) δ ppm: 166.98, 152.63 (d, $J_{C\text{-}F}$ = 244 Hz), 135.60 (d, $J_{C\text{-}F}$ = 8.3 Hz), 125.01 (d, $J_{C\text{-}F}$ = 1.6 Hz), 122.99 (d, $J_{C\text{-}F}$ = 10.1 Hz), 121.44, 115.45 (d, $J_{C\text{-}F}$ = 19.4 Hz), 50.28, 44.52, 26.40, 22.63, 21.06, 20.91. MS (ESI$^+$) m/z 302.02 $[M+H]^+$.

2-Bromo-*N*-(2,4-dimethylphenyl)-4-methylpentanamide (S5e).

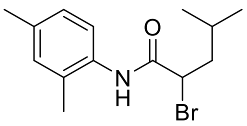

Compound **S5e** was synthesized according to general procedure A-1, using 2,4-dimethylaniline (110 µL, 0.85 mmol), 2-bromo-4-methylpentanoic acid **S6** (200 mg, 1.02 mmol) and EDC·HCl (196 mg, 1.02 mmol) in DCM (20 mL). The reaction was stirred at room temperature overnight. The crude product was purified using column chromatography (Hex/EtOAc=9/1 to 85/15). The product was obtained as white solid (215 mg, 85%). ^{1}H NMR (500 MHz, $CDCl_3$) δ ppm: 7.95 (br s, 1H), 7.64 (d, J = 7.9 Hz, 1H), 7.06–7.01 (m, 2H), 4.51 (dd, J = 9.8, 5.1 Hz, 1H), 2.31 (s, 3H), 2.27 (s, 3H), 2.13–1.92 (m, 3H), 1.02

(d, *J* = 6.6 Hz, 3H), 0.97 (d, *J* = 6.4 Hz, 3H). ^{13}C NMR (126 MHz, $CDCl_3$) δ ppm: 167.11, 135.44, 132.36, 131.21, 129.51, 127.32, 122.83, 51.48, 44.83, 26.45, 22.71, 20.93, 20.89, 17.59. MS (ESI$^+$) m/z 298.00 $[M+H]^+$.

2-Bromo-*N*-(4-methoxyphenyl)-4-methylpentanamide (S5f).

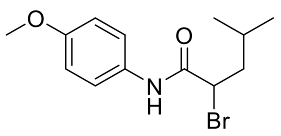

Compound **S5f** was synthesized according to general procedure A-1, using 4-methoxyaniline (141 µL, 1.14 mmol), 2-bromo-4-methylpentanoic acid **S6** (267 mg, 1.37 mmol) and EDC·HCl (262 mg, 1.37 mmol) in DCM (20 mL). The reaction was stirred at room temperature for 3 h. After the extraction, the product was obtained as white solid (334 mg, 98%) and used in the next step without further purification. ^{1}H NMR (500 MHz, $CDCl_3$) δ ppm: 7.93 (br s, 1H), 7.47–7.42 (m, 2H), 6.92–6.87 (m, 2H), 4.45 (dd, *J* = 9.5, 5.3 Hz, 1H), 3.81 (s, 3H), 2.10–1.88 (m, 3H), 1.01 (*d*, *J* = 6.6 Hz, 3H), 0.96 (d, *J* = 6.6 Hz, 3H). ^{13}C NMR (126 MHz, $CDCl_3$) δ ppm: 166.92, 156.87, 130.21, 121.83, 114.21, 55.49, 50.83, 44.64, 26.43, 22.67, 21.02. MS (ESI$^+$) m/z 300.01 $[M+H]^+$.

***N*-(4-acetylphenyl)-2-bromo-4-methylpentanamide (S5g).**

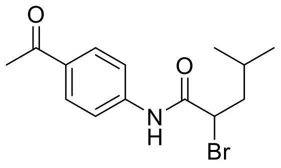

Compound **S5g** was synthesized according to general procedure A-1, using 4′-aminoacetophenone (118 mg, 0.87 mmol), 2-bromo-4-methylpentanoic acid **S6** (204 mg, 1.04 mmol) and EDC·HCl (200 mg, 1.04 mmol) in DCM (20 mL). The reaction was stirred at room temperature overnight. The crude product was purified using column chromatography (Hex/EtOAc=8/2 to 7/3). The product was obtained as white solid (144 mg, 53%). ^{1}H NMR (500 MHz, $CDCl_3$) δ ppm: 8.17 (br s, 1H), 8.00–7.95 (m, 2H), 7.68–7.65 (m, 2H), 4.47 (dd, *J* = 9.5, 5.5 Hz, 1H), 2.60 (s, 3H), 2.10–1.97 (m, 2H), 1.96–1.89 (m, 1 H), 1.02 (d, *J* = 6.7 Hz, 3H), 0.97 (d, *J* = 6.6 Hz, 3H). ^{13}C NMR (126 MHz, $CDCl_3$) δ ppm: 196.83, 167.25, 141.43, 133.48, 129.74, 119.07, 50.35, 44.41, 26.47, 26.40, 22.64, 21.02. MS (ESI$^+$) m/z 312.03 $[M+H]^+$.

2-Bromo-*N*-(4-fluorophenyl)-4-methylpentanamide (S5h).

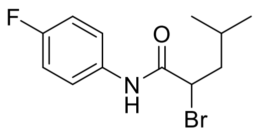

Compound **S5h** was synthesized according to general procedure A-1, using 4-fluoroaniline (125 mg, 1.12 mmol), 2-bromo-4-methylpentanoic acid **S6** (263 mg, 1.35 mmol) and EDC·HCl (258 mg, 1.35 mmol) in DCM (20 mL). The reaction was stirred at room temperature overnight. The crude product was purified using column chromatography (Hex/EtOAc=8/2). The product was obtained as beige solid (282 mg, 87%). ^{1}H NMR (500 MHz, $CDCl_3$) δ ppm: 8.03 (br s, 1H), 7.53–7.47 (m, 2H), 7.09–7.01 (m, 2H),

4.45 (dd, J = 9.5, 5.4 Hz, 1H), 2.09–1.96 (m, 2H), 1.96–1.87 (m, 1H), 1.01 (d, J = 6.6 Hz, 3H), 0.96 (d, J = 6.6 Hz, 3H). ^{13}C NMR (126 MHz, $CDCl_3$) δ ppm: 167.11, 159.74 (d, $J_{C\text{-}F}$ = 245 Hz), 133.15 (d, $J_{C\text{-}F}$ = 3.7 Hz), 121.87 (d, $J_{C\text{-}F}$ = 8.3 Hz), 115.77 (d, $J_{C\text{-}F}$ = 22.1 Hz), 50.54, 44.53, 26.41, 22.65, 21.02. ^{19}F NMR (470 MHz, $CDCl_3$) δ ppm: -117.02. MS (ESI$^+$) m/z 287.93 [M+H]$^+$.

2-Bromo-N-(4-chlorophenyl)-4-methylpentanamide (S5i).

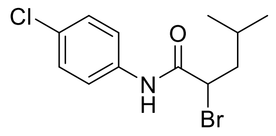

Compound **S5i** was synthesized according to general procedure A-1, using 4-chloroaniline (65 mg, 0.50 mmol), 2-bromo-4-methylpentanoic acid **S6** (195 mg, 1 mmol) and EDC·HCl (191 mg, 1 mmol) in DCM (20 mL). The reaction was stirred at room temperature for 24 h. The crude product was purified using column chromatography DCM to DCM/MeOH 1%. The product was obtained as white solid (95 mg, 62%). ^{1}H NMR (500 MHz, Acetone) δ ppm: 9.80 (br., s, 1H), 7.84 – 7.57 (m, 2H), 7.43 – 7.13 (m, 2H), 4.57 (dd, J = 8.5, 6.3 Hz, 1H), 1.98 – 1.85 (m, 2H), 1.85 – 1.76 (m, 1H), 0.96 (d, J = 6.6 Hz, 3H), 0.92 (d, J = 6.6 Hz, 3H). ^{13}C NMR (126 MHz, Acetone) δ ppm: 167.46, 137.51, 128.75, 128.56, 121.29, 58.08, 43.25, 25.20, 22.16, 21.13. MS (ESI$^+$) m/z 304.03 [M+H]$^+$.

2-Bromo-*N*-(4-bromophenyl)-4-methylpentanamide (S5j).

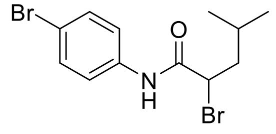

Compound **S5j** was synthesized according to general procedure A-1, using 4-bromoaniline (153 mg, 0.89 mmol), 2-bromo-4-methylpentanoic acid **S6** (210 mg, 1.07 mmol) and EDC·HCl (206 mg, 1.07 mmol) in DCM (20 mL). The reaction was stirred at room temperature for 5 h. The crude product was purified using column chromatography (Hex/EtOAc=9/1). The product was obtained as white solid (160 mg, 51%). ^{1}H NMR (500 MHz, $CDCl_3$) δ ppm: 8.02 (br s, 1H), 7.50–7.41 (m, 4H), 4.44 (dd, J = 9.6, 5.3 Hz, 1H), 2.08–1.95 (m, 2H), 1.95–1.86 (m, 1H), 1.01 (d, J = 6.7 Hz, 3H), 0.96 (d, J = 6.6 Hz, 3H). ^{13}C NMR (126 MHz, $CDCl_3$) δ ppm: 167.07, 136.22, 132.05, 121.42, 117.61, 50.54, 44.43, 26.37, 22.66, 20.97. MS (ESI$^+$) m/z 348.00 [M+H]$^+$.

2-Bromo-*N*-(4-cyanophenyl)-4-methylpentanamide (S5k).

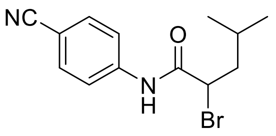

Compound **S5k** was synthesized according to general procedure A-1, using 4-aminobenzonitrile (131 mg, 1.11 mmol), 2-bromo-4-methylpentanoic **S6** acid (260 mg, 1.33 mmol) and EDC·HCl (256 mg, 1.33 mmol) in DCM (20 mL). The reaction was stirred at room temperature overnight. The crude product was purified using column chromatography (Hex/EtOAc=7/3). The product was obtained as white solid (195 mg, 60%). ^{1}H NMR (500 MHz, $CDCl_3$) δ ppm: 8.18 (br s, 1H), 7.78–7.69 (m, 2H), 7.67–7.56 (m, 2H),

4.46 (dd, J = 9.4, 5.6 Hz, 1H), 2.08–1.97 (m, 2H), 1.96–1.87 (m, 1H), 1.02 (d, J = 6.7 Hz, 3H), 0.97 (d, J = 6.6 Hz, 3H). ^{13}C NMR (126 MHz, $CDCl_3$) δ ppm: 167.39, 141.18, 133.33, 119.70, 118.58, 107.95, 50.10, 44.30, 26.39, 22.62, 21.01. MS (ESI$^-$) m/z 293.06 [M-H]$^-$.

2-Bromo-4-methyl-*N*-(4-((trifluoromethyl)sulfonyl)phenyl)pentanamide (S5l).

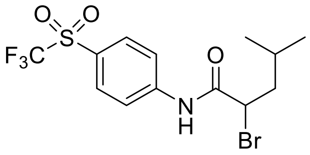

Compound **S5l** was synthesized according to general procedure A-1, using 4-((trifluoromethyl)sulfonyl)aniline (236 mg, 1.05 mmol), 2-bromo-4-methylpentanoic acid **S6** (246 mg, 1.26 mmol) and EDC·HCl (242 mg, 1.26 mmol) in DCM (20 mL). The reaction was stirred at room temperature for 48 h. The crude product was purified using column chromatography (Hex/EtOAc=7/3). The product was obtained as white solid (156 mg, 37%). ^{1}H NMR (500 MHz, $CDCl_3$) δ ppm: 8.32 (br s, 1H), 8.03 (d, J = 8.7 Hz, 2H), 7.89 (d, J = 8.7 Hz, 2H), 4.48 (dd, J = 9.2, 5.7 Hz, 1H), 2.09–1.97 (m, 2H), 1.95–1.87 (m, 1H), 1.03 (d, J = 6.7 Hz, 3H), 0.97 (d, J = 6.6 Hz, 3H). ^{13}C NMR (126 MHz, $CDCl_3$) δ ppm: 167.65, 144.70, 132.44, 125.99, 119.84, 119.80 (q, $J_{C\text{-}F}$ = 326 Hz), 49.86, 44.23, 26.42, 22.61, 21.04. MS (ESI$^-$) m/z 400.04 [M-H]$^-$.

2-Bromo-4-methyl-*N*-(naphthalen-1-yl)pentanamide (S5m).

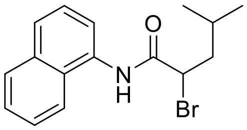

Compound **S5m** was synthesized according to general procedure A-1, using naphthalen-1-amine (122 mg, 0.85 mmol), 2-bromo-4-methylpentanoic acid **S6** (200 mg, 1.02 mmol) and EDC·HCl (197 mg, 1.02 mmol) in DCM (20 mL). The reaction was stirred at room temperature for 4 h. The crude product was purified using column chromatography (Hex/EtOAc=9/1). The product was obtained as pale pink solid (198 mg, 72%). ^{1}H NMR (500 MHz, DMSO-d_6) δ ppm: 10.37 (s, 1H), 8.05 (d, J = 8.4 Hz, 1H), 7.97 (d, J = 7.9 Hz, 1H), 7.82 (d, J = 8.2 Hz, 1H), 7.67 (d, J = 7.0 Hz, 1H), 7.62–7.55 (m, 2H), 7.52 (t, J = 7.9 Hz, 1H), 4.93 (t, J = 7.6 Hz, 1H), 2.04–1.97 (m, 1H), 1.95–1.88 (m, 1H), 1.79–1.68 (m, 1H), 1.01 (d, J = 6.6 Hz, 3H), 0.94 (d, J = 6.6 Hz, 3H). ^{13}C NMR (126 MHz, DMSO-d_6) δ ppm: 168.04, 133.75, 132.61, 128.35, 127.74, 126.28, 126.23, 125.95, 125.65, 122.23, 121.89, 48.12, 42.95, 26.32, 22.21, 21.82. MS (ESI$^+$) m/z 319.99 [M+H]$^+$.

2-Bromo-4-methyl-*N*-(naphthalen-2-yl)pentanamide (S5n).

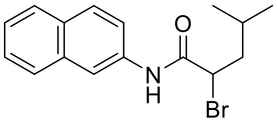

Compound **S5n** was synthesized according to general procedure A-1, using naphthalen-2-amine (150 mg, 1.05 mmol), 2-bromo-4-methylpentanoic acid **S6** (245 mg, 1.26 mmol) and EDC·HCl (241 mg, 1.26 mmol) in DCM (20 mL). The reaction was stirred at room temperature overnight. The crude

product was purified using column chromatography (Hex/EtOAc=9/1). The product was obtained as red solid (238 mg, 71%). ^{1}H NMR (500 MHz, $CDCl_3$) δ ppm: 8.24 (br s, 1H), 8.20 (br s, 1H), 7.85–7.78 (m, 3H), 7.53–7.42 (m, 3H), 4.52 (dd, J = 9.6, 5.3 Hz, 1H), 2.16–2.01 (m, 2H), 2.00–1.89 (m, 1H), 1.03 (d, J = 6.6 Hz, 3H), 0.98 (d, J = 6.6 Hz, 3H). ^{13}C NMR (126 MHz, $CDCl_3$) δ ppm: 167.22, 134.54, 133.64, 130.86, 128.88, 127.73, 127.56, 126.65, 125.34, 119.59, 116.91, 50.71, 44.58, 26.43, 22.67, 21.07. MS (ESI$^+$) m/z 320.01 $[M+H]^+$.

2-Bromo-*N*-(2,3-dihydro-1*H*-inden-5-yl)-4-methylpentanamide (S5o).

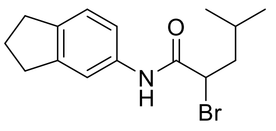

Compound **S5o** was synthesized according to general procedure A-1, using 2,3-dihydro-1*H*-inden-5-amine (144 mg, 1.08 mmol), 2-bromo-4-methylpentanoic acid **S6** (254 mg, 1.30 mmol) and EDC·HCl (250 mg, 1.30 mmol) in DCM (20 mL). The reaction was stirred at room temperature overnight. The crude product was purified using column chromatography (Hex/EtOAc=85/15). The product was obtained as beige solid (290 mg, 86%). ^{1}H NMR (500 MHz, $CDCl_3$) δ ppm: 8.24 (br s, 1H), 7.48 (s, 1H), 7.24–7.16 (m, 2H), 4.45 (dd, J = 9.5, 5.5 Hz, 1H), 2.93–2.85 (m, 4H), 2.11–1.88 (m, 5H), 1.00 (d, J = 6.7 Hz, 3H), 0.96 (d, J = 6.6 Hz, 3H). ^{13}C NMR (126 MHz, $CDCl_3$) δ ppm: 166.96, 145.30, 141.03, 135.22, 124.52, 118.12, 116.46, 50.79, 44.58, 32.95, 32.34, 26.41, 25.59, 22.64, 21.08. MS (ESI$^+$) m/z 310.01 $[M+H]^+$.

2-Bromo-N-(5-chloropyridin-2-yl)-4-methylpentanamide (S5p).

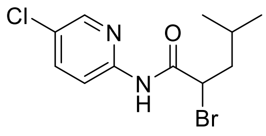

Compound **S5p** was synthesized according to general procedure A-1, using 5-chloropyridin-2-amine (65 mg, 0.50 mmol), 2-bromo-4-methylpentanoic acid **S6** (195 mg, 1 mmol) and EDC·HCl (191 mg, 1 mmol) in DCM (20 mL). The reaction was stirred at room temperature for 24 h. The crude product was purified using column chromatography DCM to DCM/MeOH 1%. The product was obtained as white solid (46 mg, 30%). ^{1}H NMR (500 MHz, Acetone) δ ppm: 9.88 (br., s, 1H), 8.28 (d, J = 2.6 Hz, 1H), 8.24 (d, J = 8.9 Hz, 1H), 7.85 (dd, J = 8.9, 2.6 Hz, 1H), 4.79 (dd, J = 8.5, 6.2 Hz, 1H), 2.01–1.76 (m, 3H), 0.98 (d, J = 6.6 Hz, 3H), 0.95 (d, J = 6.6 Hz, 3H). ^{13}C NMR (126 MHz, Acetone) δ ppm: 167.84, 150.20, 146.60, 137.87, 126.46, 114.68, 57.62, 42.96, 25.18, 25.16, 22.05, 20.96, 20.94. MS (ESI$^+$) m/z 304.97 $[M+H]^+$.

2-Bromo-N-(6-chloropyridin-3-yl)-4-methylpentanamide (S5q).

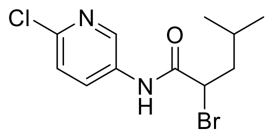

Compound **S5q** was synthesized according to general procedure A-1, using 6-chloropyridin-3-amine (65 mg, 0.50 mmol), 2-bromo-4-methylpentanoic acid **S6** (195 mg, 1 mmol) and EDC·HCl (191 mg, 1 mmol) in DCM (20 mL). The reaction was stirred at room temperature for 24 h. The crude product was purified using column chromatography DCM to DCM/MeOH 1%. The product was obtained as white solid (69 mg, 63%). ^{1}H NMR (500 MHz, $CDCl_3$) δ ppm: 8.47 (br., s, 1H), 8.38 (d, *J* = 2.8 Hz, 1H), 8.10 (dd, *J* = 8.7, 2.8 Hz, 1H), 7.26 (d, *J* = 8.7 Hz, 1H), 4.39 (dd, *J* = 9.0, 6.0 Hz, 1H), 1.97–1.85 (m, 2H), 1.79 (ddt, *J* = 19.7, 13.2, 6.5 Hz, 1H), 0.92 (d, *J* = 6.5 Hz, 3H), 0.86 (d, *J* = 6.6 Hz, 3H). ^{13}C NMR (126 MHz, $CDCl_3$) δ ppm: 166.94, 145.37, 139.76, 132.53, 129.42, 123.44, 48.24, 43.03, 25.35, 21.56, 20.12. MS (ESI^+) m/z 304.95 $[M+H]^+$.

5-(2-Bromo-4-methylpentanamido)picolinamide (S5r).

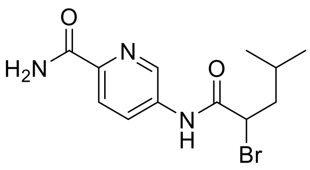

Compound **S5r** was synthesized according to general procedure A-2, using 5-aminopicolinamide (69 mg, 0.50 mmol), 2-bromo-4-methylpentanoic acid **S6** (195 mg, 1 mmol) and thionyl chloride (725 µL, 10 mmol) in THF (20 mL). The crude product was purified using column chromatography DCM to DCM/MeOH 1%. The product was obtained as white solid (31 mg, 20%). ^{1}H NMR (500 MHz, $CDCl_3$) δ ppm: 9.44 (s, 1H), 8.77 (d, *J* = 2.4 Hz, 1H), 8.35 (dd, *J* = 8.6, 2.5 Hz, 1H), 7.65 (d, *J* = 8.6 Hz, 1H), 4.44 (t, *J* = 7.6 Hz, 1H), 1.92 (t, *J* = 7.3 Hz, 1H), 1.95–1.87 (m, 2H), 1.74 (dt, *J* = 13.6, 6.7 Hz, 1H), 0.90 (d, *J* = 6.7 Hz, 3H), 0.84 (d, *J* = 6.6 Hz, 3H). MS (ESI^+) m/z 314.90 $[M+H]^+$.

Methyl 5-(2-bromo-4-methylpentanamido)picolinate (S5s).

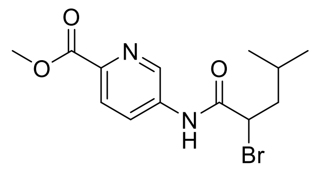

Compound **S5s** was synthesized according to general procedure A-2, using methyl 5-aminopicolinate (76 mg, 0.50 mmol), 2-bromo-4-methylpentanoic acid **S6** (195 mg, 1 mmol) and thionyl chloride (725 µL, 10 mmol) in THF (20 mL). The crude product was purified using column chromatography DCM to DCM/MeOH 1%. The product was obtained as white solid (38 mg, 23%). ^{1}H NMR (500 MHz, $CDCl_3$) δ ppm: 10.45 (s, 1H), 8.78 (d, *J* = 2.5 Hz, 1H), 8.39 (dd, *J* = 8.6, 2.5 Hz, 1H), 8.03 (d, *J* = 8.6 Hz, 1H), 4.48 (dd, *J* = 8.4, 6.4 Hz, 1H), 3.88 (s, 3H), 1.94–1.65 (m, 3H), 0.87 (d, *J* = 6.6 Hz, 3H), 0.82 (d, *J* = 6.5 Hz, 3H). MS (ESI^+) m/z 328.97 $[M+H]^+$.

2-Bromo-N-(2-chloropyrimidin-5-yl)-4-methylpentanamide (S5t).

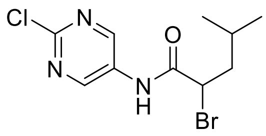

Compound **S5t** was synthesized according to general procedure A-1, using 2-chloropyrimidin-5-amine (65 mg, 0.50 mmol), 2-bromo-4-methylpentanoic acid **S6** (195 mg, 1 mmol) and EDC·HCl (191 mg, 1 mmol) in DCM (20 mL). The reaction was stirred at room temperature for 24 h. The crude product was purified using column chromatography DCM to DCM/MeOH 1%. The product was obtained as white solid (97 mg, 63%). ^{1}H NMR (500 MHz, $CDCl_3$) δ ppm: 8.90 (s, 2H), 4.41 (dd, *J* = 9.2, 5.8 Hz, 1H), 1.98–1.92 (m, 1H), 1.80–1.71 (m, 2H), 0.94 (d, *J* = 6.7 Hz, 3H), 0.89 (d, *J* = 6.6 Hz, 3H). ^{13}C NMR (126 MHz, $CDCl_3$) δ ppm: 174.67, 155.94, 150.46, 131.89, 53.48, 43.26, 26.32, 22.36, 21.56. MS (ESI$^+$) m/z 305.91 [M+H]$^+$.

2-Bromo-N-(6-chloropyridazin-3-yl)-4-methylpentanamide (S5u).

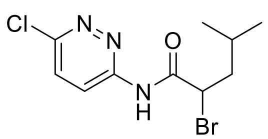

Compound **S5u** was synthesized according to general procedure A-2, using 6-chloropyridazin-3-amine (65 mg, 0.50 mmol), 2-bromo-4-methylpentanoic acid **S6** (195 mg, 1 mmol) and thionyl chloride (725 µL, 10 mmol) in THF (20 mL). The crude product was purified using column chromatography DCM to DCM/MeOH 1%. The product was obtained as white solid (31 mg, 20%). ^{1}H NMR (500 MHz, $CDCl_3$) δ ppm: 8.50 (d, *J* = 9.3 Hz, 1H), 7.53 (d, *J* = 9.4 Hz, 1H), 4.72 (t, *J* = 7.3 Hz, 1H), 1.90 – 1.80 (m, 2H), 1.79 – 1.66 (m, 1H), 0.89 (d, *J* = 6.6 Hz, 3H), 0.85 (d, *J* = 6.6 Hz, 3H). MS (ESI$^+$) m/z 305.91 [M+H]$^+$.

2-Chloro-3-phenyl-*N*-(*p*-tolyl)propanamide (S10a).

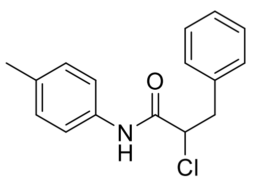

Compound **S10a** was synthesized according to general procedure A-1, using *p*-toluidine (116 mg, 1.08 mmol), 2-chloro-3-phenylpropanoic acid **S9a** (300 mg, 1.62 mmol) and EDC·HCl (311 mg, 1.62 mmol) in DCM (20 mL). The reaction was stirred at room temperature overnight. The crude product was purified using column chromatography (Hex/EtOAc=9/1). The product was obtained as beige solid (136 mg, 31%). ^{1}H NMR (500 MHz, $CDCl_3$) δ ppm: 8.02 (br s, 1H), 7.37–7.27 (m, 7H), 7.15 (d, *J* = 8.2 Hz, 2H), 4.68 (dd, *J* = 7.8, 4.4 Hz, 1H), 3.54 (dd, *J* = 14.3, 4.4 Hz, 1H), 3.31 (dd, *J* = 14.3, 7.8 Hz, 1H), 2.34 (s, 3H). ^{13}C NMR (126 MHz, $CDCl_3$) δ ppm: 166.05, 135.94, 134.92, 134.11, 129.72, 129.56, 128.44, 127.29, 120.34, 61.87, 41.39, 20.90. MS (ESI$^+$) m/z 273.96 [M+H]$^+$.

2-Chloro-3-phenyl-*N*-(4-(trifluoromethyl)phenyl)propanamide (S10b).

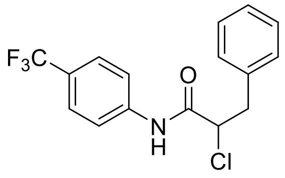

Compound **S10b** was synthesized according to general procedure A-1, using 4-(trifluoromethyl)aniline (160 μL, 1.30 mmol), 2-chloro-3-phenylpropanoic acid **S9a** (360 mg, 1.95 mmol) and EDC·HCl (374 mg, 1.95 mmol) in DCM (20 mL). The reaction was stirred at room temperature for 48 h. The crude product was purified using column chromatography (Hex/EtOAc=8/2). The product was obtained as pale yellow solid (218 mg, 51%). ^{1}H NMR (500 MHz, $CDCl_3$) δ ppm: 8.18 (br s, 1H), 7.61 (s, 4H), 7.35–7.27 (m, 5H), 4.71 (dd, J = 7.6, 4.5 Hz, 1H), 3.53 (dd, J = 14.3, 4.4 Hz, 1H), 3.34 (dd, J = 14.3, 7.6 Hz, 1H). ^{13}C NMR (126 MHz, $CDCl_3$) δ ppm: 166.47, 139.69, 135.55, 129.68, 128.53, 127.46, 126.96 (q, $J_{C\text{-}F}$ = 33 Hz), 126.40–126.25 (m), 123.91 (q, $J_{C\text{-}F}$ = 272 Hz), 118.81, 61.67, 41.29. MS (ESI$^+$) m/z 328.04 [M+H]$^+$.

2-Chloro-3-cyclohexyl-*N*-(*p*-tolyl)propanamide (S10c).

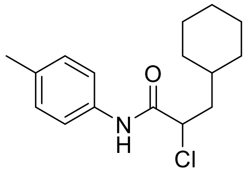

Compound **S10c** was synthesized according to general procedure A-1, using *p*-toluidine (126 mg, 1.18 mmol), 2-chloro-3-cyclohexylpropanoic acid **S9b** (404 mg, 2.12 mmol) and EDC·HCl (406 mg, 2.12 mmol) in DCM (20 mL). The reaction was stirred at room temperature overnight. The crude product was purified using column chromatography (Hex/EtOAc=9/1 to 8/2). The product was obtained as brown oil (127 mg, 38%). ^{1}H NMR (500 MHz, $CDCl_3$) δ ppm: 8.20 (br s, 1H), 7.43 (d, J = 8.4 Hz, 2H), 7.16 (d, J = 8.2 Hz, 2H), 4.50 (dd, J = 10.3, 4.2 Hz, 1H), 2.34 (s, 3H), 2.12–2.06 (m, 1H), 1.90–1.60 (m, 7H), 1.33–1.15 (m, 3H), 1.08–0.91 (m, 2H). ^{13}C NMR (126 MHz, $CDCl_3$) δ ppm: 167.36, 134.67, 134.46, 129.56, 120.05, 59.57, 43.11, 34.48, 33.56, 31.52, 26.36, 26.13, 25.90, 20.88. MS (ESI$^+$) m/z 280.03 [M+H]$^+$.

2-Chloro-3-cyclohexyl-*N*-(4-(trifluoromethyl)phenyl)propanamide (S10d).

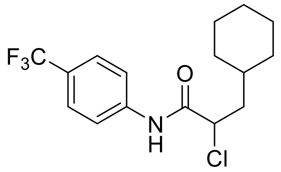

Compound **S10d** was synthesized according to general procedure A-1, using 4-(trifluoromethyl)aniline (216 mg, 1.34 mmol), 2-chloro-3-cyclohexylpropanoic acid **S9b** (460 mg, 2.41 mmol) and EDC·HCl (462 mg, 2.41 mmol) in DCM (20 mL). The reaction was stirred at room temperature for 48 h. The crude product was purified using column chromatography (Hex/EtOAc=9/1 to 8/2). The product was obtained as yellow solid (180 mg, 40%). ^{1}H NMR (500 MHz, $CDCl_3$) δ ppm: 8.39 (br s, 1H), 7.70 (d, J = 8.5 Hz, 2H), 7.62 (d, J = 8.7 Hz, 2H), 4.53 (dd, J = 10.3, 4.2 Hz, 1H), 2.12–2.06 (m, 1H), 1.91–1.62 (m, 7H), 1.35–1.15 (m, 3H), 1.10–0.90 (m,

2H). ^{13}C NMR (126 MHz, $CDCl_3$) δ ppm: 167.77, 140.04, 126.78 (q, $J_{C\text{-}F}$ = 33 Hz), 126.45–126.25 (m), 123.94 (q, $J_{C\text{-}F}$ = 273 Hz), 119.58, 59.40, 42.99, 34.45, 33.53, 31.49, 26.32, 26.10, 25.86. ^{19}F NMR (470 MHz, $CDCl_3$) δ ppm: -62.22. MS (ESI^-) m/z 332.17 $[M\text{-}H]^-$.

2-Bromo-*N*-(4'-methoxyphenyl)pentanamide (S13a).

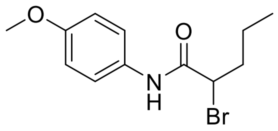

Compound **S13a** was synthesized according to general procedure A-1, using 4-methoxyaniline (1.73 g, 14.1 mmol), 2-bromopentanoic acid **S12** (2.20 mL, 16.8 mmol) and EDC·HCl (3.21 g, 16.7 mmol) in DCM (10 mL). The reaction was stirred for 18 h at rt. After extraction, **S13a** was obtained as white solid without further purification (4.48 g, quant.). ^{1}H NMR (300 MHz, $CDCl_3$) δ 7.99 (s, 1H), 7.43 (d, *J* = 9.0 Hz, 2H), 6.88 (d, *J* = 9.0 Hz, 2H), 4.43 (dd, *J* = 5.2, 8.2 Hz, 1H), 3.80 (s, 3H), 2.25–1.97 (m, 2H), 1.65–1.45 (m, 2H), 0.98 (t, *J* = 7.3 Hz, 3H). ^{13}C NMR (126 MHz, $CDCl_3$) δ 166.84, 157.16, 130.36, 122.08, 114.41, 55.67, 52.40, 38.15, 20.74, 13.42. HRMS (ESI^+) *m/z* calculated for $C_{12}H_{17}BrNO_2$ 286.0438 $[M+H]^+$, found 286.0427.

2-Bromo-*N*-(3',4'-dichlorophenyl)pentanamide (S13b).

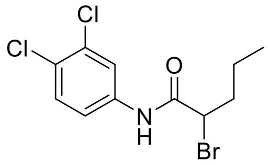

Compound **S13b** was synthesized according to general procedure A-1, using 3,4-dichloroaniline (1.60 g, 9.90 mmol), 2-bromopentanoic acid **S12** (1.50 mL, 11.4 mmol) and EDC·HCl (2.20 g, 11.5 mmol) in DCM (10 mL). The reaction was stirred for 19 h at rt. After extraction, **S13b** was obtained as white solid without further purification (3.06 g, 95%). ^{1}H NMR (300 Hz, $CDCl_3$) δ 8.08 (s, 1H), 7.77 (d, *J* = 2.2 Hz, 1H), 7.40 (d, *J* = 8.7 Hz, 1H), 7.35 (dd, *J* = 2.3, 8.7 Hz), 4.43 (dd, *J* = 5.3, 8.2 Hz, 1H), 2.24–2.00 (m, 2H), 1.64–1.44 (m, 2H), 0.98 (t, *J* = 7.4 Hz, 3H). ^{13}C NMR (75 MHz, $CDCl_3$) δ 167.14, 136.72, 133.15, 130.78, 128.52, 121.87, 119.36, 51.78, 37.92, 20.72, 13.38. HRMS (ESI^+) *m/z* calculated for $C_{11}H_{13}BrCl_2NO$ 323.9552 $[M+H]^+$, found 323.9552.

2-Bromo-*N*-(4'-acetylphenyl)pentanamide (S13c).

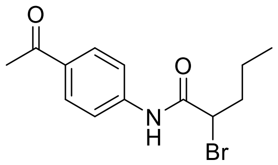

Compound **S13c** was synthesized according to general procedure A-1, using 4-aminoacetophenone (300 mg, 2.22 mmol), 2-bromopentanoic acid **S12** (350 µL, 2.55 mmol) and EDC·HCl (515 mg, 2.69 mmol) in DCM (10 mL). The reaction was stirred overnight at room temperature. After extraction, **S13c** was obtained as yellow oil without further purification (676 mg, quant.). ^{1}H NMR (300 MHz, $CDCl_3$) δ 8.24 (*br* s, 1H), 7.97 (d, *J* = 8.7 Hz, 2H), 7.66 (d, *J* = 8.7 Hz, 2H), 4.46 (dd, *J* = 5.4, 8.1 Hz, 1H), 2.59 (s, 3H), 2.26–2.02 (m, 2H), 1.66–1.45 (m, 1H), 0.98 (t, *J* = 7.4 Hz, 3H).

^{13}C NMR (75 MHz, $CDCl_3$) δ 197.01, 167.16, 141.53, 133.64, 129.88, 119.25, 51.83, 37.87, 26.63, 20.71, 13.36. HRMS (ESI) *m/z* calculated for $C_{13}H_{17}BrNO_2$ 298.0437 $[M+H]^+$, found 298.0422.

Diethyl (4-methyl-1-oxo-1-(*p*-tolylamino)pentan-2-yl)phosphonate (S7a).

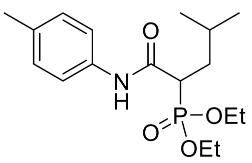

Compound **S7a** was synthesized according to general procedure C, using **S5a** (242 mg, 0.85 mmol) and triethyl phosphite (1.5 mL, 8.5 mmol). The crude product was purified using column chromatography (Hex/EtOAc = 1/1). The product was obtained as white solid (114 mg, 39%). ^1H NMR (500 MHz, $CDCl_3$) δ ppm: 8.41 (s, 1H), 7.39 (d, *J* = 8.4 Hz, 2H), 7.08 (d, *J* = 8.4 Hz, 2H), 4.21–4.08 (m, 4H), 2.98 (ddd, *J* = 22.6, 11.2, 3.5 Hz, 1H), 2.28 (s, 3H), 2.09–1.99 (m, 1H), 1.77–1.68 (m, 1H), 1.61–1.52 (m, 1H), 1.32 (q, *J* = 7.1 Hz, 6H), 0.97–0.91 (m, 6H). ^{13}C NMR (126 MHz, $CDCl_3$) δ ppm: 165.56 (d, J_{C-P} = 1.8 Hz), 135.32, 133.85, 129.38, 119.78, 63.01 (d, J_{C-P} = 7.4 Hz), 62.84 (d, J_{C-P} = 6.4 Hz), 45.22 (d, J_{C-P} = 129 Hz), 35.82 (d, J_{C-P} = 4.6 Hz), 26.60 (d, J_{C-P} = 13.8 Hz), 23.20, 21.21, 20.84, 16.42 (d, J_{C-P} = 1.8 Hz), 16.37 (d, J_{C-P} = 2.8 Hz). MS (ESI^+) m/z 342.2 $[M+H]^+$.

Diethyl (4-methyl-1-oxo-1-((4-(trifluoromethyl)phenyl)amino)pentan-2-yl)phosphonate (S7b).

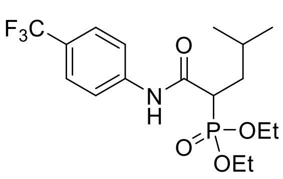

Compound **S7b** was synthesized according to general procedure C, using **S5b** (190 mg, 0.56 mmol) and triethyl phosphite (0.96 mL, 5.6 mmol). The crude product was purified using column chromatography (Hex/EtOAc = 1/1). The product was obtained as white solid (182 mg, 82%). ^1H NMR (500 MHz, $CDCl_3$) δ ppm: 9.36 (s, 1H), 7.60 (d, *J* = 8.5 Hz, 2H), 7.44 (d, *J* = 8.5 Hz, 2H), 4.30–4.09 (m, 4H), 3.14 (ddd, *J* = 22.8, 11.2, 3.3 Hz, 1H), 2.15–2.07 (m, 1H), 1.73–1.65 (m, 1H), 1.60–1.52 (m, 1H), 1.38 (t, *J* = 7.1 Hz, 3H), 1.36 (t, *J* = 7.1 Hz, 3H), 0.96 (d, *J* = 6.6 Hz, 3H), 0.94 (d, *J* = 6.6 Hz, 3H). ^{13}C NMR (126 MHz, $CDCl_3$) δ ppm: 166.34 (d, J_{C-P} = 1.8 Hz), 141.12, 126.03–125.89 (m), 125.66 (q, J_{C-F} = 33 Hz), 124.04 (q, J_{C-F} = 272 Hz), 119.11, 63.58 (d, J_{C-P} = 7.4 Hz), 62.56 (d, J_{C-P} = 7.4 Hz), 45.34 (d, J_{C-P} = 129 Hz), 35.76 (d, J_{C-P} = 5.5 Hz), 26.62 (d, J_{C-P} = 13.8 Hz), 23.18, 21.20, 16.42 (d, J_{C-P} = 6.4 Hz), 16.36 (d, J_{C-P} = 6.4 Hz). ^{31}P NMR (202 MHz, $CDCl_3$) δ ppm: 25.99. MS (ESI^+) m/z 396.10 $[M+H]^+$.

Diethyl (1-((3,4-dichlorophenyl)amino)-4-methyl-1-oxopentan-2-yl)phosphonate (S7c).

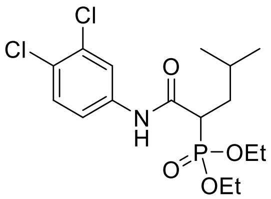

Compound **S7c** was synthesized according to general procedure C, using **S5c** (285 mg, 0.84 mmol) and triethyl phosphite (1.44 mL, 8.4 mmol). The crude product was purified using column chromatography (Hex/EtOAc = 7/3 to 1/1). The product was obtained as colorless film (274 mg, 82%). ^{1}H NMR (500 MHz, $CDCl_3$) δ ppm: 9.91 (s, 1H), 7.63 (d, J = 2.4 Hz, 1H), 7.32 (dd, J = 8.8, 2.4 Hz, 1H), 7.12 (d, J = 8.7 Hz, 1H), 4.36–4.13 (m, 4H), 3.28–3.20 (m, 1H), 2.19–2.09 (m, 1H), 1.72–1.56 (m, 1H), 1.55–1.46 (m, 1H), 1.42 (t, J = 7.1 Hz, 3H), 1.38 (t, J = 7.1 Hz, 3H), 0.94 (d, J = 6.7 Hz, 3H), 0.94 (d, J = 6.6 Hz, 3H). ^{13}C NMR (126 MHz, $CDCl_3$) δ ppm: 166.25 (d, $J_{C\text{-}P}$ = 2.8 Hz), 137.89, 132.16, 129.87, 126.65, 120.77, 118.28, 64.08 (d, $J_{C\text{-}P}$ = 6.4 Hz), 62.06 (d, $J_{C\text{-}P}$ = 6.4 Hz), 45.31 (d, $J_{C\text{-}P}$ = 130 Hz), 35.82 (d, $J_{C\text{-}P}$ = 6.4 Hz), 26.53 (d, $J_{C\text{-}P}$ = 14.7 Hz), 23.23, 21.16, 16.46 (d, $J_{C\text{-}P}$ = 5.5 Hz), 16.36 (d, $J_{C\text{-}P}$ = 6.4 Hz). ^{31}P NMR (202 MHz, $CDCl_3$) δ ppm: 25.87. MS (ESI$^+$) m/z 396.06 [M+H]$^+$.

Diethyl (1-((2-fluoro-4-methylphenyl)amino)-4-methyl-1-oxopentan-2-yl)phosphonate (S7d).

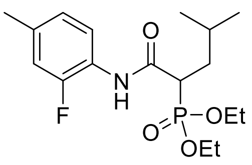

Compound **S7d** was synthesized according to general procedure C, using **S5d** (233 mg, 0.77 mmol) and triethyl phosphite (1.32 mL, 7.7 mmol). The crude product was purified using column chromatography (Hex/EtOAc = 7/3 to 1/1). The product was obtained as transparent oil (208 mg, 75%). ^{1}H NMR (500 MHz, $CDCl_3$) δ ppm: 8.34 (br s, 1H), 8.07 (t, J = 8.4 Hz, 1H), 6.93 (s, 1H), 6.91 (d, J = 4.7 Hz, 1H), 4.22–4.13 (m, 4H), 2.99 (ddd, J = 22.9, 11.1, 3.5 Hz, 1H), 2.32 (s, 3H), 2.10–2.01 (m, 1H), 1.79–1.71 (m, 1H), 1.70–1.61 (m, 1H), 1.34 (td, J = 7.0, 1.8 Hz, 6H), 0.96 (t, J = 6.5 Hz, 6H). ^{13}C NMR (126 MHz, $CDCl_3$) δ ppm: 165.80 (d, $J_{C\text{-}P}$ = 2.8 Hz), 152.62 (d, $J_{C\text{-}F}$ = 245 Hz), 134.99 (d, $J_{C\text{-}F}$ = 7.4 Hz), 124.82 (d, $J_{C\text{-}F}$ = 2.8 Hz), 123.48 (d, $J_{C\text{-}F}$ = 10.1 Hz), 121.81, 115.44 (d, $J_{C\text{-}F}$ = 18.4 Hz), 62.98 (d, $J_{C\text{-}P}$ = 4.6 Hz), 62.92 (d, $J_{C\text{-}P}$ = 4.6 Hz), 45.58 (d, $J_{C\text{-}P}$ = 129 Hz), 35.74 (d, $J_{C\text{-}P}$ = 4.6 Hz), 26.67 (d, $J_{C\text{-}P}$ = 13.8 Hz), 23.13, 21.22, 20.85, 16.36 (d, $J_{C\text{-}P}$ = 1.8 Hz), 16.32 (d, $J_{C\text{-}P}$ = 1.8 Hz). ^{31}P NMR (202 MHz, $CDCl_3$) δ ppm: 25.55. MS (ESI$^+$) m/z 360.16 [M+H]$^+$.

Diethyl (1-((2,4-dimethylphenyl)amino)-4-methyl-1-oxopentan-2-yl)phosphonate (S7e).

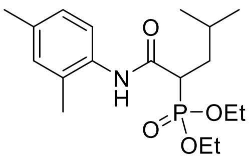

Compound **S7e** was synthesized according to general procedure C, using **S5e** (210 mg, 0.70 mmol) and triethyl phosphite (1.2 mL, 7.0 mmol). The crude product was purified using column chromatography (Hex/EtOAc = 7/3 to

3/7). The product was obtained as white solid (159 mg, 64%). ^{1}H NMR (500 MHz, $CDCl_3$) δ ppm: 8.14 (br s, 1H), 7.70 (d, *J* = 8.7 Hz, 1H), 7.02–6.99 (m, 2H), 4.24–4.09 (m, 4H), 3.08–2.96 (m, 1H), 2.29 (s, 3H), 2.28 (s, 3H), 2.09–1.99 (m, 1H), 1.80–1.74 (m, 1H), 1.69–1.54 (m, 1H), 1.37–1.31 (m, 6H), 0.99–0.96 (m, 6H). ^{13}C NMR (126 MHz, $CDCl_3$) δ ppm: 165.72, 134.60, 133.18, 131.11, 129.00, 127.06, 122.57, 62.90–62.85 (m), 45.09 (d, J_{C-P} = 129 Hz), 36.21 (d, J_{C-P} = 4.6 Hz), 26.62 (d, J_{C-P} = 14.7 Hz), 23.24, 21.17, 20.82, 17.75, 16.45–16.30 (m). ^{31}P NMR (202 MHz, $CDCl_3$) δ ppm: 26.77. MS (ESI^+) m/z 356.16 $[M+H]^+$.

Diethyl (1-((4-methoxyphenyl)amino)-4-methyl-1-oxopentan-2-yl)phosphonate (S7f).

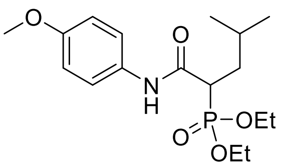

Compound **S7f** was synthesized according to general procedure C, using **S5f** (330 mg, 1.10 mmol) and triethyl phosphite (1.9 mL, 11.0 mmol). The crude product was purified using column chromatography (Hex/EtOAc = 7/3 to 1/1). The product was obtained as beige solid (148 mg, 38%). ^{1}H NMR (500 MHz, $CDCl_3$) δ ppm: 8.36 (s, 1H), 7.46–7.41 (m, 2H), 6.86–6.82 (m, 2H), 4.22–4.11 (m, 4H), 3.79 (s, 3H), 2.97 (ddd, *J* = 22.6, 11.1, 3.6 Hz, 1H), 2.10–2.01 (m, 1H), 1.80–1.71 (m, 1H), 1.63–1.55 (m, 1H), 1.34 (q, *J* = 6.8 Hz, 6H), 0.98–0.94 (m, 6H). ^{13}C NMR (126 MHz, $CDCl_3$) δ ppm: 165.51 (d, J_{C-P} = 2.8 Hz), 156.34, 131.08, 121.50, 114.05, 63.01 (d, J_{C-P} = 7.4 Hz), 62.84 (d, J_{C-P} = 6.4 Hz), 55.46, 45.14 (d, J_{C-P} = 129 Hz), 35.86 (d, J_{C-P} = 5.5 Hz), 26.63 (d, J_{C-P} = 13.8 Hz), 23.18, 21.25, 16.42 (d, J_{C-P} = 2.8 Hz), 16.37 (d, J_{C-P} = 1.8 Hz). ^{31}P NMR (202 MHz, $CDCl_3$) δ ppm: 26.46. MS (ESI^+) m/z 358.17 $[M+H]^+$.

Diethyl (1-((4-acetylphenyl)amino)-4-methyl-1-oxopentan-2-yl)phosphonate (S7g).

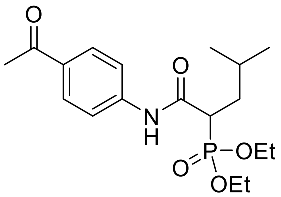

Compound **S7g** was synthesized according to general procedure C, using **S5g** (141 mg, 0.45 mmol) and triethyl phosphite (0.8 mL, 4.5 mmol). The crude product was purified using column chromatography (EtOAc/MeOH = 95/5). The product was obtained as white solid (102 mg, 61%). ^{1}H NMR (500 MHz, $CDCl_3$) δ ppm: 9.46 (br s, 1H), 7.78–7.74 (m, 2H), 7.56 (d, *J* = 8.4 Hz, 2H), 4.28–4.11 (m, 4H), 3.24–3.13 (m, 1H), 2.51 (s, 3H), 2.19–2.07 (m, 1H), 1.74–1.66 (m, 1H), 1.60–1.51 (m, 1H), 1.38 (t, *J* =7.0 Hz, 3H), 1.36 (t, *J* = 7.0 Hz, 3H), 0.96 (d, *J* = 6.7 Hz, 3H), 0.94 (d, *J* = 6.4 Hz, 3H). ^{13}C NMR (126 MHz, $CDCl_3$) δ ppm: 196.90, 166.39 (d, J_{C-P} = 2.8 Hz), 142.50, 132.47, 129.40, 118.72, 63.66 (d, J_{C-P} = 6.4 Hz), 62.48 (d, J_{C-P} = 7.4 Hz), 45.39 (d, J_{C-P} = 129 Hz), 35.72 (d, J_{C-P} = 4.6 Hz), 26.60 (d, J_{C-P} = 13.8 Hz), 26.32, 23.19, 21.21, 16.42 (d, J_{C-P} = 6.4 Hz), 16.36 (d, J_{C-P} = 5.5 Hz). ^{31}P NMR (202 MHz, $CDCl_3$) δ ppm: 25.84. MS (ESI^+) m/z 370.14 $[M+H]^+$.

Diethyl (1-((4-fluorophenyl)amino)-4-methyl-1-oxopentan-2-yl)phosphonate (S7h).

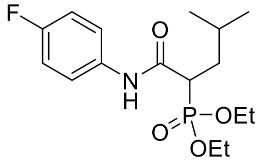

Compound **S7h** was synthesized according to general procedure C, using **S5h** (250 mg, 0.87 mmol) and triethyl phosphite (1.5 mL, 8.7 mmol). The crude product was purified using column chromatography (Hex/EtOAc = 7/3 to 3/7). The product was obtained as beige solid (223 mg, 74%). ^{1}H NMR (500 MHz, $CDCl_3$) δ ppm: 8.81 (br s, 1H), 7.49–7.44 (m, 2H), 6.97–6.91 (m, 2H), 4.26–4.08 (m, 4H), 3.10–3.00 (m, 1H), 2.13–2.03 (m, 1H), 1.78–1.68 (m, 1H), 1.62–1.52 (m, 1H), 1.35 (q, J =7.2 Hz, 6H), 0.96 (d, J = 6.6 Hz, 3H), 0.94 (d, J = 6.6 Hz, 3H). ^{13}C NMR (126 MHz, $CDCl_3$) δ ppm: 165.81 (d, $J_{C\text{-}P}$ = 1.8 Hz), 159.21 (d, $J_{C\text{-}F}$ = 244 Hz), 134.05 (d, $J_{C\text{-}F}$ = 1.8 Hz), 121.37 (d, $J_{C\text{-}F}$ = 7.4 Hz), 115.39 (d, $J_{C\text{-}F}$ = 23.0 Hz), 63.26 (d, $J_{C\text{-}P}$ = 6.4 Hz), 62.67 (d, $J_{C\text{-}P}$ = 7.4 Hz), 45.13 (d, $J_{C\text{-}P}$ = 128.7 Hz), 35.79 (d, $J_{C\text{-}P}$ = 4.6 Hz), 26.62 (d, $J_{C\text{-}P}$ = 13.8 Hz), 23.19, 21.22, 16.45–16.32 (m). ^{31}P NMR (202 MHz, $CDCl_3$) δ ppm: 26.26. ^{19}F NMR (470 MHz, $CDCl_3$) δ ppm: -118.45. MS (ESI^+) m/z 346.09 $[M+H]^+$.

Diethyl (1-((4-chlorophenyl)amino)-4-methyl-1-oxopentan-2-yl)phosphonate (S7i).

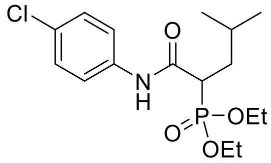

Compound **S7i** was synthesized according to general procedure C, using **S5i** (90 mg, 0.29 mmol) and triethyl phosphite (0.53 mL, 2.9 mmol). The residue was purified by automated column chromatography (Hex/EtOAc = 1/1) to give the desired product (76 mg, 71%). MS (ESI^+) m/z 362.17 $[M+H]^+$.

Diethyl (1-((4-bromophenyl)amino)-4-methyl-1-oxopentan-2-yl)phosphonate (S7j).

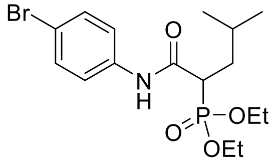

Compound **S7j** was synthesized according to general procedure C, using **S5j** (155 mg, 0.44 mmol) and triethyl phosphite (0.8 mL, 4.4 mmol). The crude product was purified using column chromatography (Hex/EtOAc = 7/3 to 1/1). The product was obtained as colorless crystals (120 mg, 66%). ^{1}H NMR (500 MHz, $CDCl_3$) δ ppm: 9.00 (br s, 1H), 7.42–7.37 (m, 2H), 7.36–7.30 (m, 2H), 4.26–4.10 (m, 4H), 3.07 (ddd, J = 22.7, 11.2, 3.3 Hz, 1H), 2.14–2.03 (m, 1H), 1.76–1.65 (m, 1H), 1.60–1.50 (m, 1H), 1.35 (dt, J = 9.4, 7.1 Hz, 6H), 0.95 (dd, J = 9.3, 6.6 Hz, 6H). ^{13}C NMR (126 MHz, $CDCl_3$) δ ppm: 165.92 (d, $J_{C\text{-}P}$ = 1.8 Hz), 137.14, 131.68, 121.12, 116.60, 63.39 (d, $J_{C\text{-}P}$ = 7.4 Hz), 62.59 (d, $J_{C\text{-}P}$ = 7.4 Hz), 45.24 (d, $J_{C\text{-}P}$ = 129 Hz), 35.75 (d, $J_{C\text{-}P}$ = 5.5 Hz), 26.60 (d, $J_{C\text{-}P}$ = 13.8 Hz), 23.18, 21.21, 16.46–16.31 (m). ^{31}P NMR (202 MHz, $CDCl_3$) δ ppm: 26.15. MS (ESI^+) m/z 406.04 $[M+H]^+$.

Diethyl (1-((4-cyanophenyl)amino)-4-methyl-1-oxopentan-2-yl)phosphonate (S7k).

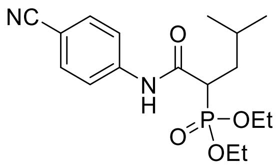

Compound **S7k** was synthesized according to general procedure C, using **S5k** (193 mg, 0.65 mmol) and triethyl phosphite (1.1 mL, 6.5 mmol). The crude product was purified using column chromatography (Hex/EtOAc = 7/3 to 3/7). The product was obtained as colorless crystals (150 mg, 65%). ^{1}H NMR (500 MHz, $CDCl_3$) δ ppm: 9.84 (br s, 1H), 7.56 (d, J = 8.9 Hz, 2H), 7.41 (d, J = 8.7 Hz, 2H), 4.31–4.12 (m, 4H), 3.22 (ddd, J = 22.8, 11.3, 3.1 Hz, 1H), 2.18–2.08 (m, 1H), 1.67–1.62 (m, 1H), 1.57–1.48 (m, 1H), 1.40 (t, J =7.2 Hz, 3H), 1.37 (t, J = 7.2 Hz, 3H), 0.95 (d, J = 6.6 Hz, 3H), 0.92 (d, J = 6.6 Hz, 3H). ^{13}C NMR (126 MHz, $CDCl_3$) δ ppm: 166.72 (d, J_{C-P} = 2.8 Hz), 142.20, 132.80, 119.27, 118.75, 106.61, 63.87 (d, J_{C-P} = 6.4 Hz), 62.40 (d, J_{C-P} = 7.4 Hz), 45.43 (d, J_{C-P} = 130 Hz), 35.79 (d, J_{C-P} = 5.5 Hz), 26.58 (d, J_{C-P} = 14.7 Hz), 23.16, 21.16, 16.41 (d, J_{C-P} = 5.5 Hz), 16.34 (d, J_{C-P} = 5.5 Hz). ^{31}P NMR (202 MHz, $CDCl_3$) δ ppm: 25.57. MS (ESI$^-$) m/z 351.20 [M-H]$^-$.

Diethyl (4-methyl-1-oxo-1-((4-((trifluoromethyl)sulfonyl)phenyl)amino)pentan-2-yl)phosphonate (S7l).

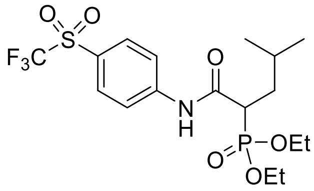

Compound **S7l** was synthesized according to general procedure C, using **S5l** (151 mg, 0.38 mmol) and triethyl phosphite (0.7 mL, 3.8 mmol). The crude product was purified using column chromatography (Hex/EtOAc = 7/3 to 3/7). The product was obtained as white crystals (113 mg, 66%). ^{1}H NMR (500 MHz, $CDCl_3$) δ ppm: 10.01 (br s, 1H), 7.83 (d, J = 8.0 Hz, 2H), 7.78 (d, J = 9.0 Hz, 2H), 4.32–4.10 (m, 4H), 3.21 (ddd, J = 22.9, 11.2, 3.1 Hz, 1H), 2.19–2.08 (m, 1H), 1.70–1.62 (m, 1H), 1.60–1.51 (m, 1H), 1.40 (t, J =6.8 Hz, 3H), 1.38 (t, J = 6.8 Hz, 3H), 0.96 (d, J = 6.4 Hz, 3H), 0.93 (d, J = 6.6 Hz, 3H). ^{13}C NMR (126 MHz, $CDCl_3$) δ ppm: 167.04 (d, J_{C-P} = 2.8 Hz), 145.64, 132.04, 124.68, 119.43, 119.77 (q, J_{C-F} = 326 Hz), 63.93 (d, J_{C-P} = 6.4 Hz), 62.69 (d, J_{C-P} = 6.4 Hz), 45.51 (d, J_{C-P} = 130 Hz), 35.69 (d, J_{C-P} = 5.5 Hz), 26.62 (d, J_{C-P} = 14.7 Hz), 23.13, 21.13, 16.50–16.30 (m). ^{31}P NMR (202 MHz, $CDCl_3$) δ ppm: 25.31. ^{19}F NMR (470 MHz, $CDCl_3$) δ ppm: -78.65. MS (ESI$^+$) m/z 460.05 [M+H]$^+$.

Diethyl (4-methyl-1-(naphthalen-1-ylamino)-1-oxopentan-2-yl)phosphonate (S7m).

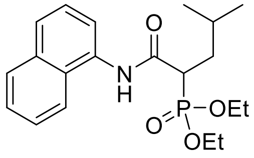

Compound **S7m** was synthesized according to general procedure C, using **S5m** (195 mg, 0.61 mmol) and triethyl phosphite (1.0 mL, 6.1 mmol). The crude product was purified using column chromatography (Hex/EtOAc = 7/3

to 3/7). The product was obtained as colorless crystals (161 mg, 70%). ^{1}H NMR (500 MHz, $CDCl_3$) δ ppm: 9.01 (s, 1H), 8.16 (d, *J* = 8.8 Hz, 1H), 8.05 (d, *J* = 7.5 Hz, 1H), 7.87 (d, *J* = 7.9 Hz, 1H), 7.68 (d, *J* = 8.2 Hz, 1H), 7.60–7.55 (m, 1H), 7.53–7.45 (m, 2H), 4.28–4.17 (m, 4H), 3.13 (ddd, *J* = 22.4, 11.2, 3.7 Hz, 1H), 2.22–2.09 (m, 1H), 1.90–1.81 (m, 1H), 1.74–1.67 (m, 1H), 1.37 (t, *J* =7.2 Hz, 3H), 1.34 (t, *J* = 7.2 Hz, 3H), 1.04–0.99 (m, 6H). ^{13}C NMR (126 MHz, $CDCl_3$) δ ppm: 166.13 (d, $J_{C\text{-}P}$ = 1.8 Hz), 134.00, 132.48, 128.58, 126.60, 126.46, 125.94, 125.65, 125.23, 120.77, 119.46, 63.10 (d, $J_{C\text{-}P}$ = 3.7 Hz), 63.04 (d, $J_{C\text{-}P}$ = 3.7 Hz), 45.16 (d, $J_{C\text{-}P}$ = 129 Hz), 36.16 (d, $J_{C\text{-}P}$ = 4.6 Hz), 26.72 (d, $J_{C\text{-}P}$ = 13.8 Hz), 23.24, 21.23, 16.47–16.33 (m). ^{31}P NMR (202 MHz, $CDCl_3$) δ ppm: 27.12. MS (ESI$^+$) m/z 378.12 [M+H]$^+$.

Diethyl (4-methyl-1-(naphthalen-2-ylamino)-1-oxopentan-2-yl)phosphonate (S7n).

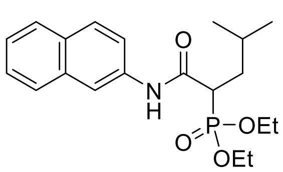

Compound **S7n** was synthesized according to general procedure C, using **S5n** (233 mg, 0.73 mmol) and triethyl phosphite (1.3 mL, 7.3 mmol). The crude product was purified using column chromatography (Hex/EtOAc = 3/7 to EtOAc). The product was obtained as pale orange crystals (246 mg, 90%). ^{1}H NMR (500 MHz, $CDCl_3$) δ ppm: 9.03 (br s, 1H), 8.21 (s, 1H), 7.70 (d, *J* = 8.1 Hz, 1H), 7.65 (t, *J* = 9.3 Hz, 2H), 7.45–7.38 (m, 2H), 7.36–7.32 (m, 1H), 4.29–4.14 (m, 4H), 3.14 (ddd, *J* = 22.7, 11.2, 3.2 Hz, 1H), 2.21–2.09 (m, 1H), 1.81–1.72 (m, 1H), 1.65–1.57 (m, 1H), 1.40–1.33 (m, 6H), 1.00–0.96 (m, 6H). ^{13}C NMR (126 MHz, $CDCl_3$) δ ppm: 165.98, 135.43, 133.64, 130.43, 128.44, 127.56, 127.37, 126.22, 124.74, 119.61, 116.28, 63.28 (d, $J_{C\text{-}P}$ = 7.4 Hz), 62.70 (d, $J_{C\text{-}P}$ = 6.4 Hz), 45.29 (d, $J_{C\text{-}P}$ = 129 Hz), 35.82 (d, $J_{C\text{-}P}$ = 4.6 Hz), 26.62 (d, $J_{C\text{-}P}$ = 14.7 Hz), 23.26, 21.21, 16.50–16.35 (m). ^{31}P NMR (202 MHz, $CDCl_3$) δ ppm: 26.39. MS (ESI$^+$) m/z 378.12 [M+H]$^+$.

Diethyl (1-((2,3-dihydro-1*H*-inden-5-yl)amino)-4-methyl-1-oxopentan-2-yl)phosphonate (S7o).

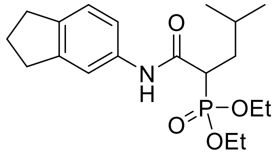

Compound **S7o** was synthesized according to general procedure C, using **S5o** (285 mg, 0.92 mmol) and triethyl phosphite (1.6 mL, 9.2 mmol). The crude product was purified using column chromatography (Hex/EtOAc = 7/3 to 3/7). The product was obtained as colorless oil (271 mg, 80%). ^{1}H NMR (500 MHz, $CDCl_3$) δ ppm: 8.59 (br s, 1H), 7.47 (br s, 1H), 7.22–7.18 (m, 1H), 7.10 (d, *J* = 8.1 Hz, 1H), 4.23–4.09 (m, 4H), 3.02 (ddd, *J* = 22.6, 11.3, 3.4 Hz, 1H), 2.89–2.80 (m, 4H), 2.12–2.01 (m, 3H), 1.78–1.69 (m, 1H), 1.62–1.53 (m, 1H), 1.37–1.31 (m, 6H), 0.97–0.93 (m, 6H). ^{13}C NMR (126 MHz, $CDCl_3$) δ ppm: 165.56 (d, $J_{C\text{-}P}$ = 1.8 Hz), 144.95, 140.04, 136.08, 124.27, 117.78,

116.14, 63.07 (d, J_{C-P} = 6.4 Hz), 62.69 (d, J_{C-P} = 6.4 Hz), 45.23 (d, J_{C-P} = 129 Hz), 35.87 (d, J_{C-P} = 5.5 Hz), 32.92, 32.30, 26.57 (d, J_{C-P} = 14.7 Hz), 25.58, 23.23, 21.17, 16.43 (d, J_{C-P} = 2.8 Hz), 16.38 (d, J_{C-P} = 3.7 Hz). ^{31}P NMR (202 MHz, $CDCl_3$) δ ppm: 26.45. MS (ESI^+) m/z 368.19 $[M+H]^+$.

Diethyl (1-((5-chloropyridin-2-yl)amino)-4-methyl-1-oxopentan-2-yl)phosphonate (S7p).

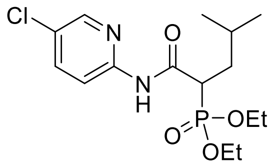

Compound **S7p** was synthesized according to general procedure C, using **S5p** (40 mg, 0.13 mmol) and triethyl phosphite (0.24 mL, 1.3 mmol). The residue was purified by automated column chromatography (Hex/EtOAc = 1/1) to give the desired product (26 mg, 55%). MS (ESI^+) m/z 363.20 $[M+H]^+$.

Diethyl (1-((6-chloropyridin-3-yl)amino)-4-methyl-1-oxopentan-2-yl)phosphonate (S7q).

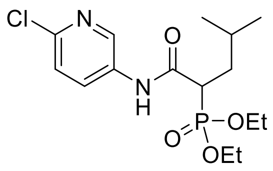

Compound **S7q** was synthesized according to general procedure C, using **S5q** (60 mg, 0.19 mmol) and triethyl phosphite (0.34 mL, 1.9 mmol). The residue was purified by automated column chromatography (Hex/EtOAc = 1/1) to give the desired product (35 mg, 49%). MS (ESI^+) m/z 363.03 $[M+H]^+$.

Diethyl (1-((6-carbamoylpyridin-3-yl)amino)-4-methyl-1-oxopentan-2-yl)phosphonate (S7r).

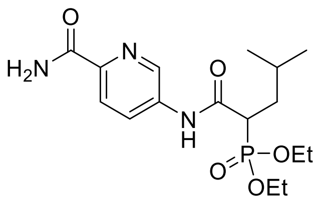

Compound **S7r** was synthesized according to general procedure C, using **S5r** (30 mg, 0.10 mmol) and triethyl phosphite (0.18 mL, 1 mmol). The residue was purified by automated column chromatography (Hex/EtOAc = 1/1) to give the desired product (14 mg, 41%). MS (ESI^+) m/z 372.10 $[M+H]^+$.

Methyl 5-(2-(diethoxyphosphoryl)-4-methylpentanamido)picolinate (S7s).

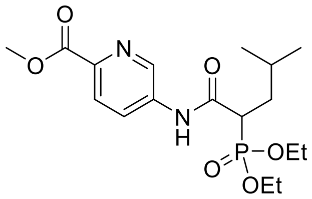

Compound **S7s** was synthesized according to general procedure C, using **S5s** (30 mg, 0.10 mmol) and triethyl phosphite (0.18 mL, 1 mmol). The residue was purified by automated column chromatography (Hex/EtOAc = 1/1) to give the desired product (17 mg, 49%). MS (ESI^+) m/z 387.07 $[M+H]^+$.

Diethyl (1-((2-chloropyrimidin-5-yl)amino)-4-methyl-1-oxopentan-2-yl)phosphonate (S7t).

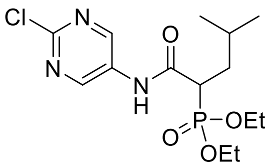

Compound **S7t** was synthesized according to general procedure C, using **S5t** (90 mg, 0.29 mmol) and triethyl phosphite (0.53 mL, 2.9 mmol). The residue was purified by automated column chromatography (Hex/EtOAc = 1/1) to give the desired product (77 mg, 72%). MS (ESI$^+$) m/z 364.10 $[M+H]^+$.

Diethyl (1-((6-chloropyridazin-3-yl)amino)-4-methyl-1-oxopentan-2-yl)phosphonate (S7u).

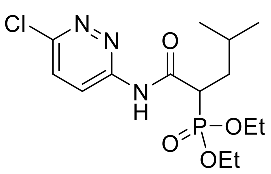

Compound **S7u** was synthesized according to general procedure C, using **S5u** (25 mg, 0.08 mmol) and triethyl phosphite (0.15 mL, 0.8 mmol). The residue was purified by automated column chromatography (Hex/EtOAc = 1/1) to give the desired product (17 mg, 57%). MS (ESI$^+$) m/z 364.11 $[M+H]^+$.

Diethyl (1-oxo-3-phenyl-1-(*p*-tolylamino)propan-2-yl)phosphonate (S11a).

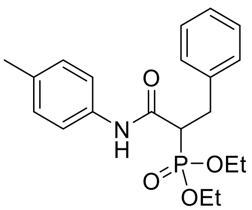

Compound **S11a** was synthesized according to general procedure C, using **S10a** (135 mg, 0.49 mmol) and triethyl phosphite (0.85 mL, 4.9 mmol). The reaction mixture was stirred for 2 weeks at 150 °C. The crude product was purified using column chromatography (Hex/EtOAc = 1/1 to 3/7). The product was obtained as colorless crystals (109 mg, 59%). ^{1}H NMR (500 MHz, $CDCl_3$) δ ppm: 8.34 (s, 1H), 7.33 (d, J = 8.4 Hz, 2H), 7.30–7.25 (m, 5H), 7.08 (d, J = 8.2 Hz, 2H), 4.24–4.07 (m, 4H), 3.44 (dt, J = 14.5, 10.1 Hz, 1H), 3.24–3.12 (m, 2H), 2.30 (s, 3H), 1.35–1.30 (m, 6H). ^{13}C NMR (126 MHz, $CDCl_3$) δ ppm: 164.69 (d, $J_{C\text{-}P}$ = 1.8 Hz), 138.97 (d, $J_{C\text{-}P}$ = 13.8 Hz), 135.17, 133.94, 129.33, 128.76, 128.51, 126.65, 119.99, 63.19 (d, $J_{C\text{-}P}$ = 6.4 Hz), 62.86 (d, $J_{C\text{-}P}$ = 6.4 Hz), 48.94 (d, $J_{C\text{-}P}$ = 128 Hz), 32.55 (d, $J_{C\text{-}P}$ = 4.6 Hz), 20.82, 16.38 (d, $J_{C\text{-}P}$ = 3.7 Hz), 16.34 (d, $J_{C\text{-}P}$ = 3.7 Hz). ^{31}P NMR (202 MHz, $CDCl_3$) δ ppm: 24.62.

Diethyl (1-oxo-3-phenyl-1-((4-(trifluoromethyl)phenyl)amino)propan-2-yl)phosphonate (S11b).

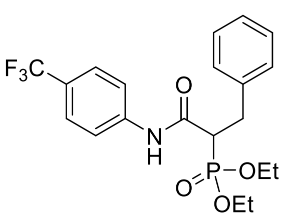

Compound **S11b** was synthesized according to general procedure C, using **S10b** (192 mg, 0.58 mmol) and triethyl phosphite (1.0 mL, 5.8 mmol). The reaction mixture was stirred for 1 week at 150 °C. The crude product was purified using column chromatography (Hex/EtOAc = 1/1). The product was obtained as colorless crystals (184 mg, 73%). ^{1}H NMR (500

MHz, $CDCl_3$) δ ppm: 9.28 (s, 1H), 7.52 (d, *J* = 8.4 Hz, 2H), 7.40 (d, *J* = 8.7 Hz, 2H), 7.30–7.18 (m, 5H), 4.28–4.05 (m, 4H), 3.44 (dt, *J* = 14.0, 9.8 Hz, 1H), 3.38–3.30 (m, 1H), 3.17–3.10 (m, 1H), 1.39 (t, *J* = 7.1 Hz, 3H), 1.32 (t, *J* = 7.1 Hz, 3H). ^{13}C NMR (126 MHz, $CDCl_3$) δ ppm: 165.47 (d, $J_{C\text{-}P}$ = 1.8 Hz), 140.93, 138.75 (d, $J_{C\text{-}P}$ = 13.8 Hz), 128.63, 128.57, 126.74, 125.95–125.80 (m), 125.70 (q, $J_{C\text{-}F}$ = 33 Hz), 124.00 (q, $J_{C\text{-}F}$ = 273 Hz), 119.19, 63.74 (d, $J_{C\text{-}P}$ = 6.4 Hz), 62.67 (d, $J_{C\text{-}P}$ = 7.4 Hz), 48.97 (d, $J_{C\text{-}P}$ = 128 Hz), 32.55 (d, $J_{C\text{-}P}$ = 4.6 Hz), 16.41 (d, $J_{C\text{-}P}$ = 5.5 Hz), 16.33 (d, $J_{C\text{-}P}$ = 5.5 Hz). ^{31}P NMR (202 MHz, $CDCl_3$) δ ppm: 24.22. ^{19}F NMR (470 MHz, $CDCl_3$) δ ppm: -62.25. MS (ESI$^+$) m/z 430.03 [M+H]$^+$.

Diethyl (3-cyclohexyl-1-oxo-1-(*p*-tolylamino)propan-2-yl)phosphonate (S11c).

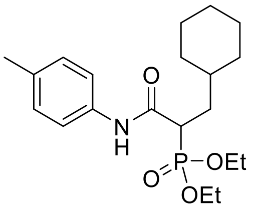

Compound **S11c** was synthesized according to general procedure C, using **S10c** (124 mg, 0.44 mmol) and triethyl phosphite (0.75 mL, 4.4 mmol). The reaction mixture was stirred for 10 days at 150 °C. The crude product was purified using column chromatography (Hex/EtOAc = 1/1 to 3/7). The product was obtained as colorless crystals (81 mg, 48%). ^{1}H NMR (500 MHz, $CDCl_3$) δ ppm: 8.39 (s, 1H), 7.42 (d, *J* = 8.4 Hz, 2H), 7.11 (d, *J* = 8.2 Hz, 2H), 4.20–4.10 (m, 4H), 3.07–2.97 (m, 1H), 2.31 (s, 3H), 2.06–1.97 (m, 1H), 1.86 (br d, *J* = 12.7 Hz, 1H), 1.75–1.65 (m, 6H), 1.36–1.30 (m, 6H), 1.26–1.08 (m, 3H), 1.02–0.80 (m, 2H). ^{13}C NMR (126 MHz, $CDCl_3$) δ ppm: 165.59 (d, $J_{C\text{-}P}$ = 1.8 Hz), 135.36, 133.81, 129.36, 119.78, 63.00 (d, $J_{C\text{-}P}$ = 6.4 Hz), 62.81 (d, $J_{C\text{-}P}$ = 7.4 Hz), 44.37 (d, $J_{C\text{-}P}$ = 128 Hz), 35.80 (d, $J_{C\text{-}P}$ = 12.9 Hz), 34.31 (d, $J_{C\text{-}P}$ = 4.6 Hz), 33.77, 31.97, 26.40, 26.07, 25.91, 20.84, 16.46–16.34 (m). ^{31}P NMR (202 MHz, $CDCl_3$) δ ppm: 26.55. MS (ESI$^+$) m/z 382.14 [M+H]$^+$.

Diethyl (3-cyclohexyl-1-oxo-1-((4-(trifluoromethyl)phenyl)amino)propan-2-yl)phosphonate (S11d).

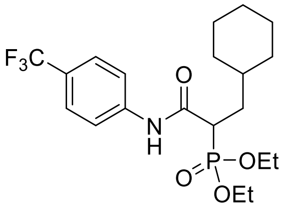

Compound **S11d** was synthesized according to general procedure C, using **S10d** (177 mg, 0.53 mmol) and triethyl phosphite (0.9 mL, 5.3 mmol). The reaction mixture was stirred for 8 days at 150 °C. The crude product was purified using column chromatography (Hex/EtOAc = 1/1 to 4/6). The product was obtained as pale-yellow solid (155 mg, 67%). ^{1}H NMR (500 MHz, $CDCl_3$) δ ppm: 9.65 (br s, 1H), 7.57 (d, *J* = 8.5 Hz, 2H), 7.37 (d, *J* = 8.4 Hz, 2H), 4.31–4.08 (m, 4H), 3.29–3.20 (m, 1H), 2.13–2.04 (m, 1H), 1.84 (br d, *J* = 12.4 Hz, 1H), 1.73–1.55 (m, 6H), 1.42–1.34 (m, 6H), 1.24–1.19 (m, 3H), 1.00–0.78 (m, 2H). ^{13}C NMR (126 MHz, $CDCl_3$) δ

ppm: 166.47 (d, J_{C-P} = 2.8 Hz), 141.22, 125.86–125.73 (m), 125.40 (q, J_{C-F} = 33 Hz), 124.02 (q, J_{C-F} = 273 Hz), 118.97, 63.73 (d, J_{C-P} = 7.4 Hz), 62.32 (d, J_{C-P} = 6.4 Hz), 44.46 (d, J_{C-P} = 129 Hz), 35.72 (d, J_{C-P} = 13.8 Hz), 34.29 (d, J_{C-P} = 5.5 Hz), 33.77, 31.90, 26.35, 26.00, 25.91, 16.49–16.25 (m). ^{31}P NMR (202 MHz, $CDCl_3$) δ ppm: 26.05. ^{19}F NMR (470 MHz, $CDCl_3$) δ ppm: -62.23. MS (ESI$^+$) m/z 436.07 [M+H]$^+$.

Diethyl (1-((4-methoxyphenyl)amino)-1-oxopentan-2-yl)phosphonate (S14a).

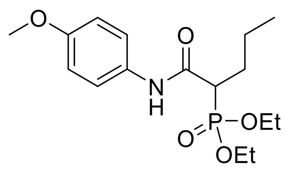

Compound **S14a** was synthesized according to general procedure C, using **S13a** (1.25 g, 4.38 mmol) and triethylphosphite (7.0 mL, 40.4 mmol). The reaction was stirred for 3 days at 150 °C. The crude product was purified using column chromatography (petroleum ether/EtOAc=9/1 to EtOAc). The product was obtained as white solid (0.94 g, 62%). ^{1}H NMR (500 Hz, DMSO-d_6) δ 9.96 (s, 1H), 7.47 (d, J = 9.0 Hz, 2H), 6.88 (d, J = 9.0 Hz, 2H), 4.10–3.95 (m, 4H), 3.71 (s, 3H), 3.05 (ddd, J = 3.5, 11.1, 21.6 Hz, 1H), 1.97–1.87 (m, 1H), 1.63–1.55 (m, 1H), 1.36–1.23 (m, 2H), 1.22 (t, J = 7.1 Hz, 3H), 1.20 (t, J = 7.1 Hz, 3H), 0.87 (t, J = 7.3 Hz, 3H). ^{13}C NMR (126 MHz, DMSO-d_6) δ 165.90 (d, J_{C-P} = 4.4 Hz), 155.34, 132.13, 120.67, 113.89, 61.91 (d, J_{C-P} = 6.2 Hz), 61.70 (d, J_{C-P} = 6.4 Hz), 55.18, 45.90 (d, J_{C-P} = 131 Hz), 28.78 (d, J_{C-P} = 5.1 Hz), 20.92 (d, J_{C-P} = 16.1 Hz), 16.32 (d, J_{C-P} = 5.6 Hz), 13.56. ^{31}P NMR (203 MHz, DMSO-d_6) δ 24.76. HRMS (ESI$^+$) m/z calculated for $C_{16}H_{27}NO_5P$ 344.1621 [M+H]$^+$, found 344.1617.

Diethyl (1-((3,4-dichlorophenyl)amino)-1-oxopentan-2-yl)phosphonate (S14b).

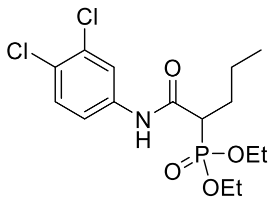

Compound **S14b** was synthesized according to general procedure C, using **S13b** (1.22 g, 3.75 mmol) and triethylphosphite (6.0 mL, 34.7 mmol). The reaction was stirred for 3 days at 150 °C. The crude product was purified using column chromatography (petroleum ether/EtOAc=9/1 to EtOAc). The product was obtained as yellow wax-like solid (1.05 g, 73%). ^{1}H NMR (500 Hz, $CDCl_3$) δ 9.83 (s, 1H), 7.63 (d, J = 2.3 Hz, 1H), 7.31 (dd, J = 2.5, 8.8 Hz, 1H), 7.12 (d, J = 8.8 Hz, 1H), 4.34–4.08 (m, 4H), 3.11 (ddd, J = 3.4, 10.9, 22.1 Hz, 1H), 2.17–2.02 (m, 1H), 1.80–1.65 (m, 1H), 1.53–1.21 (m, 2H), 1.40 (t, J = 7.1 Hz, 3H), 1.37 (t, J = 7.1 Hz, 3H), 0.93 (t, J = 7.3 Hz, 3H). ^{13}C NMR (75 MHz, $CDCl_3$) δ 166.36 (d, J_{C-P} = 4.4 Hz), 138.02, 132.35, 130.05, 126.86, 120.98, 118.50, 64.16 (d, J_{C-P} = 6.2 Hz), 62.32 (d, J_{C-P} = 6.9 Hz), 47.14 (d, J_{C-P} = 130 Hz), 29.28 (d, J_{C-}

$_{P}$ = 5.4 Hz), 21.58 (d, J_{C-P} = 15.1 Hz), 16.56 (t, J_{C-P} = 5.7 Hz), 13.76. ^{31}P NMR (203 MHz, $CDCl_3$) δ 25.48. HRMS (ESI$^+$) *m/z* calculated for $C_{15}H_{23}Cl_2NO_4P$ 382.0736 [M+H]$^+$, found 382.0734.

Diethyl (1-((4-acetylphenyl)amino)-1-oxopentan-2-yl)phosphonate (S14c).

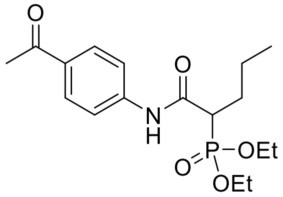

Compound **S14c** was synthesized according to general procedure C, using **S13c** (210 mg, 0.704 mmol) and triethylphosphite (2.50 mL, 14.4 mmol). The reaction was stirred for 22 h at 150 °C. The crude product was purified using column chromatography (petroleum ether/EtOAc=2/8). The product was obtained as white solid (104 mg, 42%). ^{1}H NMR (500 Hz, $CDCl_3$) δ 9.55 (s, 1H), 7.73 (d, J = 8.8 Hz, 2H), 7.55 (d, J = 8.8 Hz, 2H), 4.29–4.13 (m, 4H), 3.10 (ddd, J = 3.7, 10.7, 22.3 Hz, 1H), 2.49 (s, 3H), 2.14–2.04 (m, 1H), 1.82–1.73 (m, 1H), 1.56–1.46 (m, 1H), 1.39–1.34 (m, 7H), 0.94 (t, J = 7.3 Hz, 3H). ^{13}C NMR (126 MHz, $CDCl_3$) δ 197.03, 166.54 (d, J_{C-P} = 2.1 Hz), 142.67, 132.60, 129.52, 118.86, 63.86 (d, J_{C-P} = 6.5 Hz), 62.58 (d, J_{C-P} = 6.8 Hz), 47.25 (d, J_{C-P} = 129 Hz), 29.26 (d, J_{C-P} = 5.2 Hz), 26.43, 21.65 (d, J_{C-P} = 14.8 Hz), 16.56 (d, J_{C-P} = 6.2 Hz), 16.50 (d, J_{C-P} = 6.2 Hz), 13.81. ^{31}P NMR (203 MHz, $CDCl_3$) δ 25.50. HRMS (ESI$^+$) *m/z* calculated for $C_{17}H_{27}NO_5P$ 356.1621 [M+H]$^+$, found 356.1600.

(4-Methyl-1-oxo-1-(*p*-tolylamino)pentan-2-yl)phosphonic acid (4a).

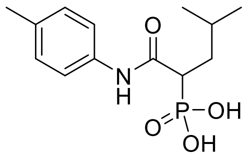

Compound **4a** was synthesized according to general procedure D, using **S7a** (110 mg, 0.32 mmol), bromotrimethylsilane (213 µL, 1.61 mmol) and DCM (6 mL). The reaction was stirred at room temperature overnight. The crude product was purified using preparative HPLC (CH_3CN (HCOOH 0.05 %)/H_2O (HCOOH 0.05 %) = 1/9 to 10/0). The product was obtained as white solid (56 mg, 71%). ^{1}H NMR (500 MHz, DMSO-d_6) δ ppm: 9.85 (s, 1H), 7.48 (d, J = 8.4 Hz, 2H), 7.08 (d, J = 8.2 Hz, 2H), 2.95 (ddd, J = 22.4, 11.4, 2.8 Hz, 1H), 2.23 (s, 3H), 1.99–1.89 (m, 1H), 1.51–1.34 (m, 2H), 0.87–0.83 (m, 6H). ^{13}C NMR (126 MHz, DMSO-d_6) δ ppm: 167.62 (d, J_{C-P} = 4.6 Hz), 137.05, 131.83, 129.01, 119.03, 45.98 (d, J_{C-P} = 127 Hz), 35.82 (d, J_{C-P} = 3.7 Hz), 26.48 (d, J_{C-P} = 14.7 Hz), 23.30, 21.41, 20.51. ^{31}P NMR (202 MHz, DMSO-d_6) δ ppm: 20.12. HRMS (ESI$^-$) calculated for $C_{13}H_{19}NO_4P$ [M-H]$^-$ 284.1057, found 284.1058.

(4-Methyl-1-oxo-1-((4-(trifluoromethyl)phenyl)amino)pentan-2-yl)phosphonic acid (4b).

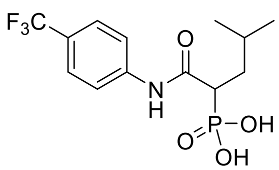

Compound **4b** was synthesized according to general procedure D, using **S7b** (169 mg, 0.43 mmol), bromotrimethylsilane (400 µL, 3.00 mmol) and DCM (15 mL). The reaction was stirred at room temperature overnight.

Next day, 5 equiv. of TMSBr were added. The crude product was purified using preparative HPLC (CH_3CN (HCOOH 0.05 %)/H_2O (HCOOH 0.05 %) = 1/9 to 10/0). The product was obtained as white solid (65 mg, 45%). ^1H NMR (500 MHz, DMSO-d_6) δ ppm: 10.36 (s, 1H), 7.81 (d, J = 8.4 Hz, 2H), 7.64 (d, J = 8.4 Hz, 2H), 3.13–2.94 (m, 1H), 2.09–1.86 (m, 1H), 1.55–1.37 (m, 2H), 0.86 (d, J = 6.0 Hz, 6H). ^{13}C NMR (126 MHz, DMSO-d_6) δ ppm: 168.74, 142.95, 126.10–125.95 (m), 124.46 (q, $J_{C\text{-}F}$ = 272 Hz), 122.06 (q, $J_{C\text{-}F}$ = 32 Hz), 118.94, 46.46 (d, $J_{C\text{-}P}$ = 121 Hz), 35.69, 26.58 (d, $J_{C\text{-}P}$ = 10.1 Hz), 23.18, 21.41m. ^{31}P NMR (202 MHz, DMSO-d_6) δ ppm: 19.33. ^{19}F NMR (470 MHz, DMSO-d_6) δ ppm: -60.26. HRMS (ESI$^-$) calculated for $C_{13}H_{16}F_3NO_4P$ [M-H]$^-$ 338.0774, found 338.0773.

(1-((3,4-Dichlorophenyl)amino)-4-methyl-1-oxopentan-2-yl)phosphonic acid (4c).

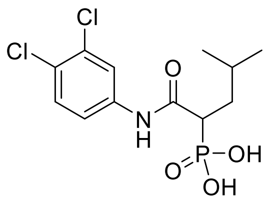

Compound **4c** was synthesized according to general procedure D, using **S7c** (255 mg, 0.64 mmol), bromotrimethylsilane (590 µL, 4.48 mmol) and DCM (15 mL). The reaction was stirred at room temperature overnight. Next day, 5 equiv. of TMSBr were added. The crude product was purified using preparative HPLC (CH_3CN (HCOOH 0.05 %)/H_2O (HCOOH 0.05 %) = 1/9 to 10/0). The product was obtained as white solid (89 mg, 41%). ^1H NMR (500 MHz, DMSO-d_6) δ ppm: 10.28 (bs, 1H), 8.03 (d, J = 2.3 Hz, 1H), 7.56–7.52 (m, 1H), 7.46 (dd, J = 8.8, 2.4 Hz, 1H), 3.00–2.90 (m, 1H), 2.01–1.90 (m, 1H), 1.52–1.38 (m, 2H), 0.85 (d, J = 5.5 Hz, 6H). ^{13}C NMR (126 MHz, DMSO-d_6) δ ppm: 168.48 (d, $J_{C\text{-}P}$ = 4.6 Hz), 139.42, 130.90, 130.60, 124.35, 120.18, 119.06, 46.29 (d, $J_{C\text{-}P}$ = 126 Hz), 35.59 (d, $J_{C\text{-}P}$ = 4.6 Hz), 26.48 (d, $J_{C\text{-}P}$ = 14.7 Hz), 23.14, 21.34. ^{31}P NMR (202 MHz, DMSO-d_6) δ ppm: 19.09. HRMS (ESI$^-$) calculated for $C_{12}H_{15}Cl_2NO_4P$ [M-H]$^-$ 338.0121, found 338.0124.

(1-((2-Fluoro-4-methylphenyl)amino)-4-methyl-1-oxopentan-2-yl)phosphonic acid (4d).

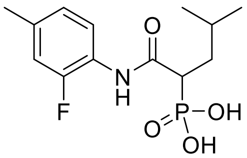

Compound **4d** was synthesized according to general procedure D, using **S7d** (203 mg, 0.56 mmol), bromotrimethylsilane (520 µL, 3.95 mmol) and DCM (15 mL). The reaction was stirred at room temperature overnight. Next day, 5 equiv. of TMSBr were added. The crude product was purified using preparative HPLC (CH_3CN (HCOOH 0.05 %)/H_2O (HCOOH 0.05 %) = 1/9 to 10/0). The product was obtained as white solid (122 mg, 72%). ^1H NMR (500 MHz, DMSO-d_6) δ ppm: 9.57 (s, 1H), 7.78 (t, J = 8.3 Hz, 1H), 7.05 (d, J = 11.9 Hz, 1H), 6.94 (d, J = 8.1 Hz, 1H), 3.13 (ddd, J = 22.5, 11.2, 2.9 Hz, 1H), 2.27 (s, 3H), 1.96–1.87 (m, 1H), 1.58–1.48 (m, 1H), 1.47–1.38 (m, 1H), 0.92–0.81 (m, 6H). ^{13}C NMR (126

MHz, DMSO-d_6) δ ppm: 168.27–168.13 (m), 153.15 (d, $J_{C\text{-}F}$ = 245 Hz), 134.71 (d, $J_{C\text{-}F}$ = 7.4 Hz), 124.55 (d, $J_{C\text{-}F}$ = 2.8 Hz), 123.75 (d, $J_{C\text{-}F}$ = 12.0 Hz), 123.55, 115.63 (d, $J_{C\text{-}F}$ = 18.4 Hz), 45.36 (d, $J_{C\text{-}P}$ = 126 Hz), 35.91 (d, $J_{C\text{-}P}$ = 4.6 Hz), 26.48 (d, $J_{C\text{-}P}$ = 14.7 Hz), 23.20, 21.40, 20.33. ^{31}P NMR (202 MHz, DMSO-d_6) δ ppm: 20.09. HRMS (ESI$^-$) calculated for $C_{13}H_{18}FNO_4P$ [M-H]$^-$ 302.0963, found 302.0962.

(1-((2,4-Dimethylphenyl)amino)-4-methyl-1-oxopentan-2-yl)phosphonic acid (4e).

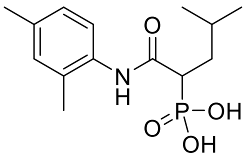

Compound **4e** was synthesized according to general procedure D, using **S7e** (150 mg, 0.42 mmol), bromotrimethylsilane (400 µL, 2.95 mmol) and DCM (5 mL). The crude product was purified using preparative HPLC (CH_3CN (HCOOH 0.05 %)/H_2O (HCOOH 0.05 %) = 1/9 to 10/0). The product was obtained as white solid (57 mg, 45%). ^{1}H NMR (500 MHz, DMSO-d_6) δ ppm: 9.21 (br s, 1H), 7.26 (d, J = 8.1 Hz, 1H), 6.99 (br s, 1 H), 6.94 (d, J = 11.9 Hz, 1H), 3.06–2.95 (m, 1H), 2.23 (s, 3H), 2.16 (s, 3H), 2.00–1.87 (m, 1H), 1.61–1.50 (m, 1H), 1.46–1.34 (m, 1H), 0.92–0.87 (m, 6H). ^{13}C NMR (126 MHz, DMSO-d_6) δ ppm: 167.74, 134.00, 133.90, 131.42, 130.70, 126.30, 124.93, 45.33 (d, $J_{C\text{-}P}$ = 127 Hz), 35.85, 26.49 (d, $J_{C\text{-}P}$ = 14.7 Hz), 23.40, 21.35, 20.56, 17.75. ^{31}P NMR (202 MHz, DMSO-d_6) δ ppm: 20.56. HRMS (ESI$^-$) calculated for $C_{14}H_{21}NO_4P$ [M-H]$^-$ 298.1214, found 298.1216.

(1-((4-Methoxyphenyl)amino)-4-methyl-1-oxopentan-2-yl)phosphonic acid (4f).

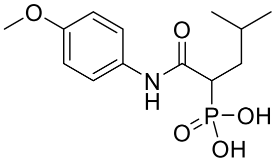

Compound **4f** was synthesized according to general procedure D, using **S7f** (145 mg, 0.41 mmol), bromotrimethylsilane (375 µL, 2.84 mmol) and DCM (10 mL). The reaction was stirred at room temperature overnight. The crude product was purified using preparative HPLC (CH_3CN (HCOOH 0.05 %)/H_2O (HCOOH 0.05 %) = 1/9 to 10/0). The product was obtained as white solid (35 mg, 29%). ^{1}H NMR (500 MHz, DMSO-d_6) δ ppm: 9.77 (s, 1H), 7.52–7.45 (m, 2H), 6.89–6.81 (m, 2H), 3.70 (s, 3H), 2.92 (ddd, J = 22.3, 11.3, 2.8 Hz, 1H), 1.99–1.90 (m, 1H), 1.52–1.35 (m, 2H), 0.89–0.82 (m, 6H). ^{13}C NMR (126 MHz, DMSO-d_6) δ ppm: 167.37 (d, $J_{C\text{-}P}$ = 4.6 Hz), 155.07, 132.72, 120.61, 113.76, 55.21, 45.92 (d, $J_{C\text{-}P}$ = 128 Hz), 35.81 (d, $J_{C\text{-}P}$ = 3.7 Hz), 26.49 (d, $J_{C\text{-}P}$ = 14.7 Hz), 23.28, 21.42. ^{31}P NMR (202 MHz, DMSO-d_6) δ ppm: 20.19. HRMS (ESI$^-$) calculated for $C_{13}H_{19}NO_5P$ [M-H]$^-$ 300.1006, found 300.1006.

(1-((4-Acetylphenyl)amino)-4-methyl-1-oxopentan-2-yl)phosphonic acid (4g).

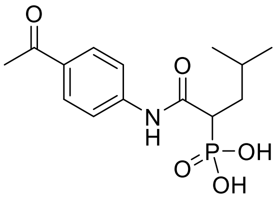

Compound **4g** was synthesized according to general procedure D, using **S7g** (99 mg, 0.27 mmol), bromotrimethylsilane (250 µL, 1.87 mmol) and DCM (10 mL). The reaction was stirred at room temperature overnight. Next day, 5 equiv. of TMSBr were added. The crude product was purified using preparative HPLC (CH_3CN (HCOOH 0.05 %)/H_2O (HCOOH 0.05 %) = 1/9 to 10/0). The product was obtained as white solid (51 mg, 61%). ^{1}H NMR (500 MHz, DMSO-*d*$_6$) δ ppm: 10.35 (s, 1H), 7.95–7.86 (m, 2H), 7.78–7.69 (m, 2H), 3.13–2.95 (m, 1H), 2.51 (s, 3H), 2.06–1.89 (m, 1H), 1.56–1.39 (m, 2H), 0.90–0.82 (m, 6H). ^{13}C NMR (126 MHz, DMSO-*d*$_6$) δ ppm: 196.51, 168.53, 143.72, 131.50, 129.42, 118.25, 35.69, 26.57, 26.43, 23.17, 21.38. ^{31}P NMR (202 MHz, DMSO-*d*$_6$) δ ppm: 19.34. HRMS (ESI$^–$) calculated for $C_{14}H_{19}NO_5P$ [M-H]$^-$ 312.1006, found 312.1005.

(1-((4-Fluorophenyl)amino)-4-methyl-1-oxopentan-2-yl)phosphonic acid (4h).

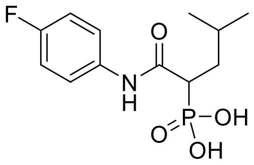

Compound **4h** was synthesized according to general procedure D, using **S7h** (199 mg, 0.58 mmol), bromotrimethylsilane (530 µL, 4.04 mmol) and DCM (15 mL). The reaction was stirred at room temperature overnight. Next day, 5 equiv. of TMSBr were added. The crude product was purified using preparative HPLC (CH_3CN (HCOOH 0.05 %)/H_2O (HCOOH 0.05 %) = 1/9 to 10/0). The product was obtained as white solid (80 mg, 48%). ^{1}H NMR (500 MHz, DMSO-*d*$_6$) δ ppm: 10.00 (br s, 1H), 7.64–7.58 (m, 2H), 7.15–7.08 (m, 2H), 3.00–2.89 (m, 1H), 2.01–1.91 (m, 1H), 1.53–1.37 (m, 2H), 0.86 (d, J = 6.0 Hz, 6H). ^{13}C NMR (126 MHz, DMSO-*d*$_6$) δ ppm: 167.78, 157.80 (d, $J_{C\text{-}F}$ = 240 Hz), 135.87 (d, $J_{C\text{-}F}$ = 2.8 Hz), 120.69 (d, $J_{C\text{-}F}$ = 8.3 Hz), 115.14 (d, $J_{C\text{-}F}$ = 22.1 Hz), 46.00 (d, $J_{C\text{-}P}$ = 127 Hz), 35.70 (d, $J_{C\text{-}P}$ = 1.8 Hz), 26.46 (d, $J_{C\text{-}P}$ = 14.7 Hz), 23.20, 21.36. ^{31}P NMR (202 MHz, DMSO-*d*$_6$) δ ppm: 19.82. ^{19}F NMR (470 MHz, DMSO-*d*$_6$) δ ppm: -119.76. HRMS (ESI$^–$) calculated for $C_{12}H_{16}FNO_4P$ [M-H]$^-$ 288.0806, found 288.0805.

(1-((4-Chlorophenyl)amino)-4-methyl-1-oxopentan-2-yl)phosphonic acid (4i).

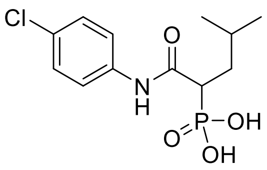

Compound **4i** was synthesized according to general procedure D, using **S7i** (70 mg, 0.19 mmol), bromotrimethylsilane (250 µL, 1.9 mmol) and DCM (15 mL). The reaction was stirred at room temperature overnight. The crude product was purified using preparative HPLC (CH_3CN (HCOOH 0.05 %)/H_2O (HCOOH 0.05 %) = 1/9 to 10/0). The product was obtained as white solid (40 mg, 69%).

^{1}H NMR (500 MHz, DMSO) δ ppm: 10.12 (br s, 1H), 7.66–7.59 (m, 2H), 7.36–7.31 (m, 2H), 2.98 (ddd, *J* = 22.5, 11.3, 2.6 Hz, 1H), 2.02–1.90 (m, 1H), 1.45 (qdd, *J* = 12.6, 9.5, 4.8 Hz, 2H), 0.86 (d, *J* = 6.4 Hz, 6H). ^{13}C NMR (126 MHz, DMSO) δ ppm: 168.54 (d, $J_{C\text{-}P}$ = 4.8 Hz), 138.86, 128.96, 126.89, 120.99, 46.60 (d, $J_{C\text{-}P}$ = 126.5 Hz), 36.13 (d, $J_{C\text{-}P}$ = 4.1 Hz), 26.93 (d, $J_{C\text{-}P}$ = 14.7 Hz), 23.64, 21.82. ^{31}P NMR (202 MHz, DMSO) δ ppm: 19.64. HRMS (ESI$^-$) calculated for $C_{12}H_{16}ClNO_4P$ [M-H]$^-$ 304.0511, found 304.0510.

(1-((4-Bromophenyl)amino)-4-methyl-1-oxopentan-2-yl)phosphonic acid (4j).

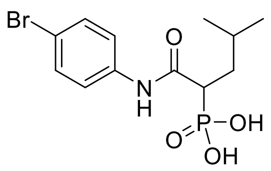

Compound **4j** was synthesized according to general procedure D, using **S7j** (117 mg, 0.29 mmol), bromotrimethylsilane (190 µL, 1.44 mmol) and DCM (5 mL). The reaction was stirred at room temperature overnight. The crude product was purified using preparative HPLC (CH_3CN (HCOOH 0.05 %)/H_2O (HCOOH 0.05 %) = 1/9 to 10/0). The product was obtained as white solid (79 mg, 78%). ^{1}H NMR (500 MHz, DMSO-d_6) δ ppm: 10.09 (s, 1H), 7.58 (d, *J* = 8.7 Hz, 2H), 7.46 (d, *J* = 8.7 Hz, 2H), 3.02–2.90 (m, 1H), 2.02–1.89 (m, 1H), 1.53–1.36 (m, 2H), 0.86 (d, *J* = 6.2 Hz, 6H). ^{13}C NMR (126 MHz, DMSO-d_6) δ ppm: 168.13, 138.77, 131.42, 120.94, 114.46, 46.18 (d, $J_{C\text{-}P}$ = 130 Hz), 35.66, 26.48 (d, $J_{C\text{-}P}$ = 13.8 Hz), 23.17, 21.36. ^{31}P NMR (202 MHz, DMSO-d_6) δ ppm: 19.56. HRMS (ESI$^-$) calculated for $C_{12}H_{16}BrNO_4P$ [M-H]$^-$ 348.0006, found 348.0006.

(1-((4-Cyanophenyl)amino)-4-methyl-1-oxopentan-2-yl)phosphonic acid (4k).

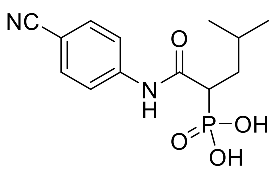

Compound **4k** was synthesized according to general procedure D, using **S7k** (138 mg, 0.39 mmol), bromotrimethylsilane (360 µL, 2.73 mmol) and DCM (10 mL). The reaction was stirred at room temperature overnight. Next day, 5 equiv. of TMSBr were added. The crude product was purified using preparative HPLC (CH_3CN (HCOOH 0.05 %)/H_2O (HCOOH 0.05 %) = 1/9 to 10/0). The product was obtained as white solid (40 mg, 35%). ^{1}H NMR (500 MHz, DMSO-d_6) δ ppm: 10.43 (br s, 1H), 7.81–7.72 (m, 4H), 3.02 (dd, *J* = 22.4, 10.4 Hz, 1H), 2.01–1.92 (m, 1H), 1.52–1.39 (m, 2H), 0.85 (d, *J* = 6.1 Hz, 6H). ^{13}C NMR (126 MHz, DMSO-d_6) δ ppm: 168.87, 143.54, 133.24, 119.15, 119.00, 104.67, 46.42 (d, $J_{C\text{-}P}$ = 122 Hz), 35.62, 26.52 (d, $J_{C\text{-}P}$ = 14.7 Hz), 23.13, 21.36. ^{31}P NMR (202 MHz, DMSO-d_6) δ ppm: 18.99. HRMS (ESI$^-$) calculated for $C_{13}H_{16}N_2O_4P$ [M-H]$^-$ 295.0853, found 295.0852.

(4-Methyl-1-oxo-1-((4-((trifluoromethyl)sulfonyl)phenyl)amino)pentan-2-yl)phosphonic acid (4l).

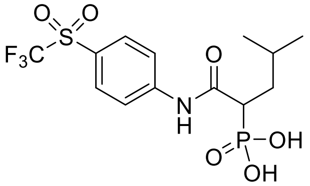

Compound **4l** was synthesized according to general procedure D, using **S7l** (110 mg, 0.24 mmol), bromotrimethylsilane (220 µL, 1.68 mmol) and DCM (10 mL). The reaction was stirred at room temperature overnight. The crude was purified using preparative HPLC (CH_3CN (HCOOH 0.05 %)/H_2O (HCOOH 0.05 %) = 1/9 to 10/0), to give the product **4l** as white solid (51 mg, 53%). ^1H NMR (500 MHz, DMSO-d_6) δ ppm: 10.77 (s, 1H), 8.07–8.04 (m, 2H), 8.03–8.00 (m, 2H), 3.08 (ddd, J = 22.5, 11.1, 2.1 Hz, 1H), 2.04–1.94 (m, 1H), 1.54–1.41 (m, 2H), 0.86 (d, J = 5.5 Hz, 6H). ^{13}C NMR (126 MHz, DMSO-d_6) δ ppm: 169.39 (d, $J_{C\text{-}P}$ = 4.6 Hz), 147.40, 132.44, 121.57, 119.91 (q, $J_{C\text{-}F}$ = 326 Hz), 119.54, 46.53 (d, $J_{C\text{-}P}$ = 125 Hz), 35.34 (d, $J_{C\text{-}P}$ = 3.7 Hz), 26.53 (d, $J_{C\text{-}P}$ = 14.7 Hz), 23.09, 21.33. ^{31}P NMR (202 MHz, DMSO-d_6) δ ppm: 18.55. ^{19}F NMR (470 MHz, DMSO-d_6) δ ppm: -78.83. HRMS (ESI$^-$) calculated for $C_{13}H_{16}F_3NO_6PS$ [M-H]$^-$ 402.0394, found 402.0393.

(4-Methyl-1-(naphthalen-1-ylamino)-1-oxopentan-2-yl)phosphonic acid (4m).

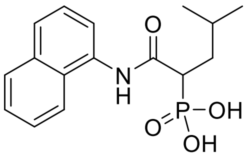

Compound **4m** was synthesized according to general procedure D, using **S7m** (155 mg, 0.41 mmol), bromotrimethylsilane (380 µL, 2.87 mmol) and DCM (5 mL). The reaction was stirred at room temperature overnight. The crude product was purified using preparative HPLC (CH_3CN (HCOOH 0.05 %)/H_2O (HCOOH 0.05 %) = 1/9 to 10/0). The product was obtained as white solid (96 mg, 73%). ^1H NMR (500 MHz, DMSO-d_6) δ ppm: 9.97 (s, 1H), 8.23–8.17 (m, 1H), 7.95–7.90 (m, 1H), 7.74 (d, J = 8.2 Hz, 1H), 7.66 (d, J = 7.2 Hz, 1H), 7.55–7.51 (m, 2H), 7.48 (t, J = 7.8 Hz, 1H), 3.23 (ddd, J = 22.6, 11.4, 2.7 Hz, 1H), 2.04–1.95 (m, 1H), 1.68–1.56 (m, 1H), 1.53–1.43 (m, 1H), 0.96 (d, J = 6.4 Hz, 3H), 0.92 (d, J = 6.6 Hz, 3H). ^{13}C NMR (126 MHz, DMSO-d_6) δ ppm: 168.55 (d, $J_{C\text{-}P}$ = 4.6 Hz), 133.82, 133.72, 128.04, 127.91, 126.06, 125.79, 125.59, 125.10, 123.06, 121.52, 45.54 (d, $J_{C\text{-}P}$ = 127 Hz), 35.86 (d, $J_{C\text{-}P}$ = 3.7 Hz), 26.65 (d, $J_{C\text{-}P}$ = 14.7 Hz), 23.36, 21.48. ^{31}P NMR (202 MHz, DMSO-d_6) δ ppm: 20.46. HRMS (ESI$^+$) calculated for $C_{16}H_{21}NO_4P$ [M+H]$^+$ 322.1203, found 322.1204.

(4-Methyl-1-(naphthalen-2-ylamino)-1-oxopentan-2-yl)phosphonic acid (4n).

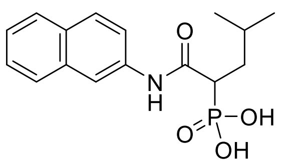

Compound **4n** was synthesized according to general procedure D, using **S7n** (245 mg, 0.65 mmol), bromotrimethylsilane (600 µL, 4.54 mmol) and DCM (10 mL). The reaction was stirred at room temperature overnight. The crude product was purified using preparative HPLC (CH_3CN (HCOOH 0.05 %)/H_2O (HCOOH 0.05 %) = 1/9 to 10/0). The product was obtained as white solid (138 mg, 66%). ^1H NMR (500 MHz, DMSO-d_6) δ ppm: 10.18 (s, 1H), 8.35–8.32 (m, 1H), 7.87–7.75 (m, 3H), 7.57 (dd, J = 8.85, 1.98 Hz, 1H), 7.48–7.43 (m, 1H), 7.41–7.36 (m, 1H), 3.11–3.00 (m, 1H), 2.06–1.95 (m, 1H), 1.57–1.39 (m, 2H), 0.95–0.82 (m, 6H). ^{13}C NMR (126 MHz, DMSO-d_6) δ ppm: 168.24 (d, $J_{C\text{-}P}$ = 3.7 Hz), 137.06, 133.48, 129.68, 128.28, 127.50, 127.30, 126.46, 124.52, 120.03, 114.99, 46.16 (d, $J_{C\text{-}P}$ = 127 Hz), 35.84 (d, $J_{C\text{-}P}$ = 2.8 Hz), 26.57 (d, $J_{C\text{-}P}$ = 14.7 Hz), 23.33, 21.43. ^{31}P NMR (202 MHz, DMSO-d_6) δ ppm: 19.86. HRMS (ESI$^+$) calculated for $C_{16}H_{21}NO_4P$ $[M+H]^+$ 322.1203, found 322.1201.

(1-((2,3-Dihydro-1*H*-inden-5-yl)amino)-4-methyl-1-oxopentan-2-yl)phosphonic acid (4o).

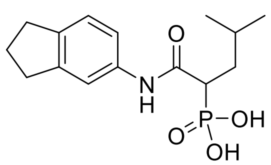

Compound **4o** was synthesized according to general procedure D, using **S7o** (270 mg, 0.73 mmol), bromotrimethylsilane (680 µL, 5.14 mmol) and DCM (10 mL). The reaction was stirred at room temperature overnight. The crude was purified using preparative HPLC (CH_3CN (HCOOH 0.05 %)/H_2O (HCOOH 0.05 %) = 1/9 to 10/0) to give the product **4o** as white solid (127 mg, 56%). ^1H NMR (500 MHz, DMSO-d_6) δ ppm: 9.81 (br s, 1H), 7.53 (s, 1H), 7.28 (d, J = 7.9 Hz, 1H), 7.10 (d, J = 8.1 Hz, 1H), 2.95 (ddd, J = 22.2, 11.3, 2.2 Hz, 1H), 2.79 (dt, J = 14.3, 7.3 Hz, 4H), 2.03–1.89 (m, 3H), 1.52–1.35 (m, 2H), 0.87–0.84 (m, 6H). ^{13}C NMR (126 MHz, DMSO-d_6) δ ppm: 168.00 (d, $J_{C\text{-}P}$ = 4.6 Hz), 144.42, 138.51, 138.15, 124.46, 117.62, 115.70, 46.39 (d, $J_{C\text{-}P}$ = 127 Hz), 36.24 (d, $J_{C\text{-}P}$ = 3.7 Hz), 32.95, 32.23, 26.91 (d, $J_{C\text{-}P}$ = 15.6 Hz), 25.66, 23.71, 21.82. ^{31}P NMR (202 MHz, DMSO-d_6) δ ppm: 20.23. HRMS (ESI$^+$) calculated for $C_{15}H_{23}NO_4P$ $[M+H]^+$ 312.1359, found 312.1359.

(1-((5-Chloropyridin-2-yl)amino)-4-methyl-1-oxopentan-2-yl)phosphonic acid (4p).

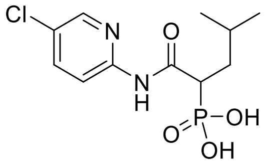

Compound **4p** was synthesized according to general procedure D, using **S7p** (20 mg, 0.05 mmol), bromotrimethylsilane (66 µL, 0.5 mmol) and DCM (15 mL). The reaction was stirred at room temperature overnight. The crude product was purified using preparative HPLC (CH_3CN

(HCOOH 0.05 %)/H_2O (HCOOH 0.05 %) = 1/9 to 10/0). The product was obtained as white solid (7.6 mg, 45%). ^{1}H NMR (500 MHz, DMSO) δ ppm: 10.58 (s, 1H), 8.35 (d, *J* = 2.5 Hz, 1H), 8.15 (d, *J* = 9.0 Hz, 1H), 7.89 (dd, *J* = 9.0, 2.7 Hz, 1H), 3.24 (ddd, *J* = 22.3, 11.0, 2.0 Hz, 1H), 2.05–1.82 (m, 1H), 1.58–1.31 (m, 2H), 0.87 (d, *J* = 4.5 Hz, 3H), 0.86 (d, *J* = 4.5 Hz, 3H). ^{13}C NMR (126 MHz, DMSO) δ ppm: 169.56 (d, J_{C-P} = 4.8 Hz), 151.19, 146.70, 138.27, 125.27, 114.85, 46.11 (d, J_{C-P} = 125.6 Hz), 36.26 (d, J_{C-P} = 4.3 Hz), 26.98 (d, J_{C-P} = 14.6 Hz), 23.66, 21.84. ^{31}P NMR (202 MHz, DMSO) δ ppm: 19.53. HRMS (ESI$^-$) calculated for $C_{11}H_{15}ClN_2O_4P$ [M-H]$^-$ 305.0463, found 305.0462.

(1-((6-Chloropyridin-3-yl)amino)-4-methyl-1-oxopentan-2-yl)phosphonic acid (4q).

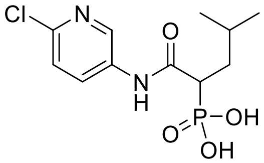

Compound **4q** was synthesized according to general procedure D, using **S7q** (30 mg, 0.08 mmol), bromotrimethylsilane (105 µL, 0.8 mmol) and DCM (15 mL). The reaction was stirred at room temperature overnight. The crude product was purified using preparative HPLC (CH_3CN (HCOOH 0.05 %)/H_2O (HCOOH 0.05 %) = 1/9 to 10/0). The product was obtained as white solid (13 mg, 52%). ^{1}H NMR (500 MHz, DMSO) δ ppm: 10.68 (br., s, 1H), 8.42 (d, *J* = 2.6 Hz, 1H), 8.21 (d, *J* = 9.0 Hz, 1H), 7.95 (dd, *J* = 9.0, 2.7 Hz, 1H), 3.38–3.19 (m, 1H), 2.07–1.94 (m, 1H), 1.64–1.38 (m, 2H), 0.93 (d, *J* = 4.6 Hz, 3H), 0.92 (d, *J* = 4.6 Hz, 3H). ^{13}C NMR (126 MHz, DMSO) δ ppm: 169.55 (d, J_{C-P} = 4.8 Hz), 151.18, 146.71, 138.29, 125.27, 114.83, 46.06 (d, J_{C-P} = 125.7 Hz), 36.24 (d, J_{C-P} = 4.2 Hz), 26.97 (d, J_{C-P} = 14.7 Hz), 23.69, 21.81. ^{31}P NMR (202 MHz, DMSO) δ ppm: 19.56. HRMS (ESI$^-$) calculated for $C_{11}H_{15}ClN_2O_4P$ [M-H]$^-$ 305.0463, found 305.0462.

(1-((6-Carbamoylpyridin-3-yl)amino)-4-methyl-1-oxopentan-2-yl)phosphonic acid (4r).

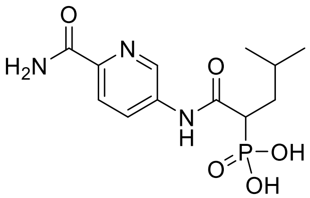

Compound **4r** was synthesized according to general procedure D, using **S7r** (10 mg, 0.026 mmol), bromotrimethylsilane (35 µL, 0.26 mmol) and DCM (15 mL). The reaction was stirred at room temperature overnight. The crude product was purified using preparative HPLC (CH_3CN (HCOOH 0.05 %)/H_2O (HCOOH 0.05 %) = 1/9 to 10/0). The product was obtained as white solid (6.7 mg, 80%). ^{1}H NMR (500 MHz, DMSO) δ ppm: 10.52 (s, 1H), 8.82 (d, *J* = 2.4 Hz, 1H), 8.19 (dd, *J* = 8.6, 2.4 Hz, 1H), 8.02 (d, *J* = 1.8 Hz, 1H), 7.99 (d, *J* = 8.6 Hz, 1H), 7.54 (d, *J* = 1.8 Hz, 1H), 3.04 (ddd, *J* = 22.4, 11.1, 2.2 Hz, 1H), 1.99 (ddd, *J* = 15.3, 10.2, 3.6 Hz, 1H), 1.57–1.38 (m, 2H), 0.87 (d, *J* = 6.3 Hz, 6H). ^{13}C NMR (126 MHz, DMSO) δ ppm: 169.31 (d, J_{C-P} = 4.8 Hz), 166.24, 145.13, 139.21, 138.72, 126.53, 122.96, 46.68 (d, J_{C-P} = 125.7 Hz), 36.05 (d, J_{C-P} =

4.0 Hz), 26.94 (d, J_{C-P} = 14.6 Hz), 23.61, 21.79. ^{31}P NMR (202 MHz, DMSO) δ ppm: 18.98. HRMS (ESI$^-$) calculated for $C_{12}H_{17}N_3O_5P$ [M-H]$^-$ 314.0911, found 314.0911.

(1-((6-(Methoxycarbonyl)pyridin-3-yl)amino)-4-methyl-1-oxopentan-2-yl)phosphonic acid (4s).

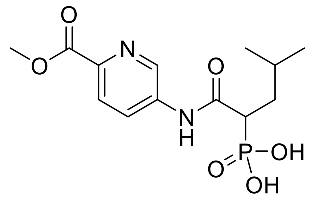

Compound **4s** was synthesized according to general procedure D, using **S7s** (15 mg, 0.04 mmol), bromotrimethylsilane (53 µL, 0.4 mmol) and DCM (15 mL). The reaction was stirred at room temperature overnight. The crude product was purified using preparative HPLC (CH_3CN (HCOOH 0.05 %)/H_2O (HCOOH 0.05 %) = 1/9 to 10/0). The product was obtained as white solid (11 mg, 83%). ^{1}H NMR (500 MHz, DMSO) δ ppm: 10.63 (s, 1H), 8.84 (d, *J* = 2.4 Hz, 1H), 8.26 (dd, *J* = 8.6, 2.4 Hz, 1H), 8.04 (d, *J* = 8.6 Hz, 1H), 3.85 (s, 3H), 3.20–2.91 (m, 1H), 1.99 (ddd, *J* = 15.0, 10.0, 3.4 Hz, 1H), 1.63–1.31 (m, 2H), 0.87 (d, *J* = 6.2 Hz, 6H). ^{13}C NMR (126 MHz, DMSO) δ ppm: 169.58 (d, J_{C-P} = 5.0 Hz), 165.21, 141.68, 140.54, 139.42, 126.18, 125.88, 52.63, 46.73 (d, J_{C-P} = 125.5 Hz), 36.03 (d, J_{C-P} = 4.0 Hz), 26.95 (d, J_{C-P} = 14.6 Hz), 23.61, 21.78. ^{31}P NMR (202 MHz, DMSO) δ ppm: 18.80. HRMS (ESI$^-$) calculated for $C_{13}H_{18}N_2O_6P$ [M-H]$^-$ 329.0908, found 329.0906.

(1-((2-Chloropyrimidin-5-yl)amino)-4-methyl-1-oxopentan-2-yl)phosphonic acid (4t) and (1-((2-bromopyrimidin-5-yl)amino)-4-methyl-1-oxopentan-2-yl)phosphonic acid (4u).

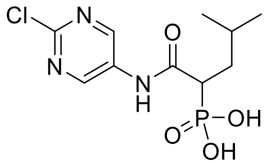

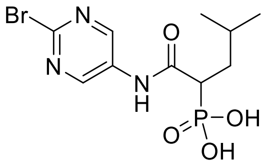

Compounds **4t** and **4u** were synthesized according to general procedure D, using **S7t** (70 mg, 0.19 mmol), bromotrimethylsilane (250 µL, 1.9 mmol) and DCM (15 mL). The reaction was stirred at room temperature overnight. The crude product was purified using preparative HPLC (CH_3CN (HCOOH 0.05 %)/H_2O (HCOOH 0.05 %) = 1/9 to 10/0). Compound **4t** was obtained as white solid (25 mg, 43%). ^{1}H NMR (500 MHz, DMSO) δ ppm: 10.67 (s, 1H), 8.96 (s, 2H), 3.00 (ddd, *J* = 22.4, 11.1, 2.3 Hz, 1H), 2.01–1.89 (m, 1H), 1.52–1.37 (m, 2H), 0.85 (d, *J* = 3.1 Hz, 3H), 0.84 (d, *J* = 3.1 Hz, 3H). ^{13}C NMR (126 MHz, DMSO) δ ppm: 169.52 (d, J_{C-P} = 4.8 Hz), 153.29, 150.11, 134.42, 46.60 (d, J_{C-P} = 125.1 Hz), 35.98 (d, J_{C-P} = 4.0 Hz), 26.87 (d, J_{C-P} = 14.4 Hz), 23.59, 21.75. ^{31}P NMR (202 MHz, DMSO) δ ppm: 18.44. HRMS (ESI$^-$) calculated for $C_{10}H_{14}ClN_3O_4P$ [M-H]$^-$ 306.0416, found 306.0414. Compound **4u** was obtained as white solid (13 mg, 19%). ^{1}H NMR (500 MHz, DMSO) δ ppm: 10.70 (s, 1H), 8.91 (s, 2H), 3.02 (ddd, *J* = 22.4, 11.2, 2.2 Hz, 1H), 1.97

(ddd, *J* = 14.8, 10.6, 3.6 Hz, 1H), 1.52–1.39 (m, 2H), 0.86 (d, *J* = 2.9 Hz, 3H), 0.84 (d, *J* = 2.8 Hz, 3H). ^{13}C NMR (126 MHz, DMSO) δ ppm: 169.49 (d, J_{C-P} = 4.8 Hz), 150.08, 144.48, 134.82, 46.58 (d, J_{C-P} = 125.2 Hz), 35.96 (d, J_{C-P} = 4.0 Hz), 26.87 (d, J_{C-P} = 14.4 Hz), 23.58, 21.74. ^{31}P NMR (202 MHz, DMSO) δ ppm: 18.55. HRMS (ESI$^-$) calculated for $C_{10}H_{14}BrN_3O_4P$ [M-H]$^-$ 349.9911, found 349.9911.

(1-((6-Chloropyridazin-3-yl)amino)-4-methyl-1-oxopentan-2-yl)phosphonic acid (4v) and (1-((6-bromopyridazin-3-yl)amino)-4-methyl-1-oxopentan-2-yl)phosphonic acid (4w).

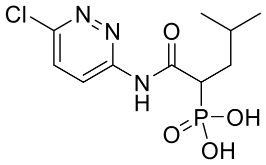

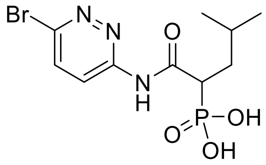

Compounds **4v** and **4w** were synthesized according to general procedure D, using **S7u** (17 mg, 0.04 mmol), bromotrimethylsilane (53 µL, 0.40 mmol) and DCM (15 mL). The reaction was stirred at room temperature overnight. The crude product was purified using preparative HPLC (CH_3CN (HCOOH 0.05 %)/H_2O (HCOOH 0.05 %) = 1/9 to 10/0). Compound **4v** was obtained as white solid (5 mg, 35%). ^{1}H NMR (500 MHz, Acetone) δ ppm: 10.69 (s, 1H), 8.42 (d, *J* = 9.3 Hz, 1H), 7.61 (d, *J* = 9.1 Hz, 1H), 2.07–2.03 (m, 2H), 1.81–1.78 (m, 1H), 1.79 (d, *J* = 6.6 Hz, 3H), 0.77 (d, *J* = 6.6 Hz, 3H). ^{13}C NMR (126 MHz, DMSO) δ ppm: 167.58 (d, J_{C-P} = 5.0 Hz), 155.20, 147.18, 130.44, 129.42, 44.37 (d, J_{C-P} = 125.3 Hz), 35.03 (d, J_{C-P} = 4.0 Hz), 26.15 (d, J_{C-P} = 14.6 Hz), 23.11, 21.75. ^{31}P NMR (202 MHz, Acetone) δ ppm: 20.29. HRMS (ESI$^-$) calculated for $C_{10}H_{14}ClN_3O_4P$ [M-H]$^-$ 306.0416, found 306.0414. Compound **4w** was obtained as white solid (3 mg, 18%). ^{1}H NMR (500 MHz, Acetone) δ ppm: 10.75 (s, 1H), 8.46 (d, *J* = 9.3 Hz, 1H), 7.86 (d, *J* = 9.3 Hz, 1H), 3.40 (dd, *J* = 23.8, 10.4 Hz, 1H), 1.95 – 1.89 (m, 1H), 1.87 – 1.69 (m, 2H), 0.90 (d, *J* = 6.6 Hz, 6H). ^{13}C NMR (126 MHz, DMSO) δ ppm: 170.01 (d, J_{C-P} = 5.0 Hz), 153.78, 149.10, 133.61, 129.24, 44.31 (d, J_{C-P} = 125.2 Hz), 35.30 (d, J_{C-P} = 4.0 Hz), 25.87 (d, J_{C-P} = 14.6 Hz), 23.09, 21.66. ^{31}P NMR (202 MHz, Acetone) δ ppm: 21.04. HRMS (ESI$^-$) calculated for $C_{10}H_{14}BrN_3O_4P$ [M-H]$^-$ 349.9911, found 349.9910.

(1-Oxo-3-phenyl-1-(*p*-tolylamino)propan-2-yl)phosphonic acid (4x).

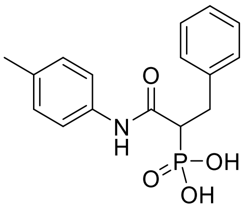

Compound **4x** was synthesized according to general procedure D, using **S11a** (96.6 mg, 0.26 mmol), bromotrimethylsilane (240 µL, 1.80 mmol) and DCM (5 mL). The reaction was stirred at room temperature for 2 days. The crude was purified using preparative HPLC (CH_3CN (HCOOH 0.05 %)/H_2O (HCOOH 0.05 %) = 1/9 to 10/0) to give the product **4x** as white solid (52.5

mg, 64%). ^{1}H NMR (500 MHz, DMSO-d_6) δ ppm: 9.76 (br s, 1H), 7.39 (d, J = 8.2 Hz, 2H), 7.25–7.21 (m, 2H), 7.19–7.11 (m, 3H), 7.03 (d, J = 8.2 Hz, 2H), 3.29–3.15 (m, 2H), 3.01–2.91 (m, 1H), 2.21 (s, 3H). ^{13}C NMR (126 MHz, DMSO-d_6) δ ppm: 166.76, 140.25 (d, J_{C-P} = 16.5 Hz), 136.85, 131.75, 128.86, 128.31, 128.24, 126.04, 118.99, 49.46 (d, J_{C-P} = 124 Hz), 32.48, 20.41. ^{31}P NMR (202 MHz, DMSO-d_6) δ ppm: 18.51. HRMS (ESI$^-$) calculated for $C_{16}H_{17}NO_4P$ [M-H]$^-$ 318.0901, found 318.0901.

(1-Oxo-3-phenyl-1-((4-(trifluoromethyl)phenyl)amino)propan-2-yl)phosphonic acid (4y).

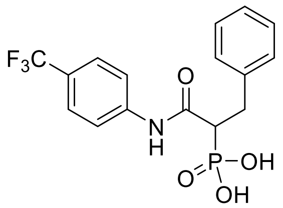

Compound **4y** was synthesized according to general procedure D, using **S11b** (166 mg, 0.39 mmol), bromotrimethylsilane (360 µL, 2.71 mmol) and DCM (10 mL). The reaction was stirred at room temperature for 2 days. The crude was purified using preparative HPLC (CH_3CN (HCOOH 0.05 %)/H_2O (HCOOH 0.05 %) = 1/9 to 10/0) to give the product **4y** as white solid (85.3 mg, 59%). ^{1}H NMR (500 MHz, DMSO-d_6) δ ppm: 10.22 (s, 1H), 7.72 (d, J = 8.5 Hz, 2H), 7.61 (d, J = 8.5 Hz, 2H), 7.26–7.21 (m, 2H), 7.19–7.12 (m, 3H), 3.32–3.21 (m, 2H), 3.04–2.95 (m, 1H). ^{13}C NMR (126 MHz, DMSO-d_6) δ ppm: 167.69, 142.71, 139.96 (d, J_{C-P} = 16.5 Hz), 128.34, 128.28, 126.17, 126.03–125.85 (m), 124.38 (q, J_{C-F} = 272 Hz), 123.00 (q, J_{C-F} = 31.6 Hz), 118.82, 49.77 (d, J_{C-P} = 124 Hz), 32.39. ^{31}P NMR (202 MHz, DMSO-d_6) δ ppm: 17.78. ^{19}F NMR (470 MHz, DMSO-d_6) δ ppm: -60.28. HRMS (ESI$^-$) calculated for $C_{16}H_{14}F_3NO_4P$ [M-H]$^-$ 372.0618, found 372.0620.

(3-Cyclohexyl-1-oxo-1-(*p*-tolylamino)propan-2-yl)phosphonic acid (4z).

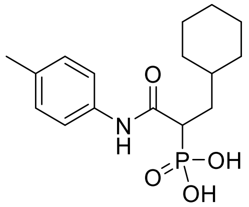

Compound **4z** was synthesized according to general procedure D, using **S11c** (74.7 mg, 0.20 mmol), bromotrimethylsilane (180 µL, 1.37 mmol) and DCM (5 mL). The reaction was stirred at room temperature for 2 days. The crude was purified using preparative HPLC (CH_3CN (HCOOH 0.05 %)/H_2O (HCOOH 0.05 %) = 1/9 to 10/0) to give the product **4z** as white solid (37.6 mg, 59%). ^{1}H NMR (500 MHz, DMSO-d_6) δ ppm: 9.83 (s, 1H), 7.48 (d, J = 8.4 Hz, 2H), 7.08 (d, J = 8.2 Hz, 2H), 2.97 (dd, J = 21.8, 10.6 Hz, 1H), 2.24 (s, 3H), 1.96–1.86 (m, 1H), 1.80 (br d, J = 12.4 Hz, 1H), 1.67–1.53 (m, 4H), 1.52–1.37 (m, 1H), 1.19–1.04 (m, 4H), 0.94–0.84 (m, 1H), 0.83–0.72 (m, 1H). ^{13}C NMR (126 MHz, DMSO-d_6) δ ppm: 167.69, 137.07, 131.84, 129.02, 119.05, 45.28 (d, J = 126 Hz), 35.84 (d, J = 13.8 Hz), 34.27, 33.54, 31.70, 26.13, 25.75 (d, J = 12.9 Hz),

20.51). ^{31}P NMR (202 MHz, DMSO-d_6) δ ppm: 20.24. HRMS (ESI$^-$) calculated for $C_{16}H_{23}NO_4P$ [M-H]$^-$ 324.1370, found 324.1371.

(3-Cyclohexyl-1-oxo-1-((4-(trifluoromethyl)phenyl)amino)propan-2-yl)phosphonic acid (4aa).

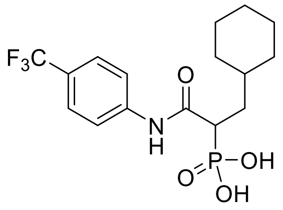

Compound **4aa** was synthesized according to general procedure D, using **S11d** (148.6 mg, 0.34 mmol), bromotrimethylsilane (315 µL, 2.39 mmol) and DCM (5 mL). The reaction was stirred at room temperature for 2 days. The crude was purified using preparative HPLC (CH_3CN (HCOOH 0.05 %)/H_2O (HCOOH 0.05 %) = 1/9 to 10/0) to give the product **4aa** as white solid (56.2 mg, 44%). ^1H NMR (500 MHz, DMSO-d_6) δ ppm: 10.34 (s, 1H), 7.81 (d, J = 8.5 Hz, 2H), 7.65 (d, J = 8.7 Hz, 2H), 3.04 (ddd, J = 22.6, 11.2, 2.1 Hz, 1H), 1.99–1.89 (m, 1H), 1.78 (br d, J = 12.5 Hz, 1H), 1.67–1.53 (m, 4H), 1.52–1.41 (m, 1H), 1.20–1.03 (m, 4H), 0.94–0.74 (m, 2H). ^{13}C NMR (126 MHz, DMSO-d_6) δ ppm: 168.68 (d, $J_{C\text{-}P}$ = 3.7 Hz), 142.99, 126.16–126.00 (m), 124.48 (q, $J_{C\text{-}F}$ = 271 Hz), 122.96 (q, $J_{C\text{-}F}$ = 32.8 Hz), 118.89, 45.60 (d, $J_{C\text{-}P}$ = 126 Hz), 35.91 (d, $J_{C\text{-}P}$ = 13.8 Hz), 34.15, 33.46, 31.71, 26.09, 25.76, 25.67. ^{31}P NMR (202 MHz, DMSO-d_6) δ ppm: 19.43. ^{19}F NMR (470 MHz, DMSO-d_6) δ ppm: -60.22. HRMS (ESI$^-$) calculated for $C_{16}H_{20}F_3NO_4P$ [M-H]$^-$ 378.1088, found 378.1091.

(1-((4-methoxyphenyl)amino)-1-oxopentan-2-yl)phosphonic acid (4ab).

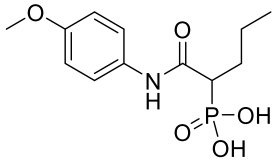

Compound **4ab** was synthesized according to general procedure D, using **S14a** (491 mg, 1.43 mmol)**,** bromotrimethylsilane (940 µL, 7.18 mmol) and DCM (6 mL). The reaction was stirred for 15 h at rt. The crude was purified using preparative HPLC (CH_3CN (HCOOH 0.05 %)/H_2O (HCOOH 0.05 %) = 3/7 to 10/0) to give the product **4ab** as white solid (360 mg, 88%). ^1H NMR (500 Hz, DMSO-d_6) δ 9.75 (s, 1H), 7.50 (d, J = 9.1 Hz, 2H), 6.86 (d, J = 9.1 Hz, 2H), 3.71 (s, 3H), 2.82 (ddd, J = 3.3, 11.2, 21.7 Hz, 1H), 1.96–1.86 (m, 1H), 1.64–1.55 (m, 1H), 1.36–1.16 (m, 2H), 0.86 (t, J = 7.3 Hz, 3H). ^{13}C NMR (126 MHz, DMSO-d_6) δ 167.25 (d, $J_{C\text{-}P}$ = 4.5 Hz), 155.01, 132.66, 120.54, 113.72, 55.16, 47.48 (d, $J_{C\text{-}P}$ = 128 Hz), 29.06 (d, $J_{C\text{-}P}$ = 4.5 Hz), 21.19 (d, $J_{C\text{-}P}$ = 15.7 Hz), 13.73. ^{31}P NMR (203 MHz, DMSO-d_6) δ 19.87. HRMS (ESI$^+$) m/z calculated for $C_{12}H_{19}NO_5P$ 288.0995 [M+H]$^+$, found 288.0992. HRMS (ESI$^-$) m/z calculated for $C_{12}H_{17}NO_5P$ 286.0850 [M-H]$^-$, found 286.0850.

(1-((3,4-Dichlorophenyl)amino)-1-oxopentan-2-yl)phosphonic acid (4ac).

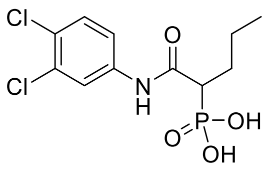

Compound **4ac** was synthesized according to general procedure D, using **S14b** (494 mg, 1.29 mmol), bromotrimethylsilane (850 µL, 6.55 mmol) and DCM (6 mL). The reaction was stirred for 15 h at rt. The crude was purified using preparative HPLC (CH_3CN (HCOOH 0.05 %)/H_2O (HCOOH 0.05 %) = 45/55 to 100/0) to give the product **4ac** as white solid (355 mg, 85%). ^{1}H NMR (500 Hz, DMSO-d_6) δ 10.27 (s, 1H), 8.04 (d, J = 2.4 Hz, 1H), 7.54 (d, J = 8.8 Hz, 1H), 7.46 (dd, J = 2.5, 8.8 Hz, 1H), 2.85 (ddd, J = 3.3, 11.2, 22.0 Hz, 1H), 1.97–1.87 (m, 1H), 1.66–1.57 (m, 1H), 1.35–1.25 (m, 1H), 1.24–1.15 (m, 1H), 0.86 (t, J = 7.4 Hz, 3H). ^{13}C NMR (126 MHz, DMSO-d_6) δ 168.42 (d, $J_{C\text{-}P}$ = 4.6 Hz), 139.39, 130.91, 130.62, 124.39, 120.21, 119.08, 47.89 (d, $J_{C\text{-}P}$ = 127 Hz), 28.88 (d, $J_{C\text{-}P}$ = 4.2 Hz), 21.19 (d, $J_{C\text{-}P}$ = 15.4 Hz), 13.69. ^{31}P NMR (162 MHz, DMSO-d_6) δ 18.81. HRMS (ESI$^+$) m/z calculated for $C_{11}H_{15}Cl_2NO_4P$ 326.0110 [M+H]$^+$, found 326.0108. HRMS (ESI$^-$) m/z calculated for $C_{11}H_{13}Cl_2NO_4P$ 323.9965 [M-H]$^-$, found 323.9967.

(1-((4-Acetylphenyl)amino)-1-oxopentan-2-yl)phosphonic acid (4ad).

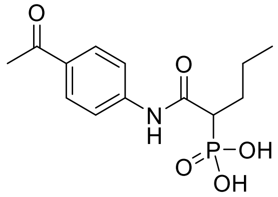

Compound **4ad** was synthesized according to general procedure D, using **S14c** (94 mg, 0.26 mmol), bromotrimethylsilane (190 µL, 1.45 mmol) and DCM (5 mL). The reaction was stirred for 21 h at rt. The crude was purified using preparative HPLC (CH_3CN (HCOOH 0.05 %)/H_2O (HCOOH 0.05 %) = 3/7 to 10/0) to give the product **4ad** as white solid (47 mg, 60%). ^{1}H NMR (500 Hz, DMSO-d_6) δ 10.25 (s, 1H), 7.91 (d, J = 8.8 Hz, 2H), 7.73 (d, J = 8.8 Hz, 2H), 2.93 (ddd, J = 3.3, 11.0, 22.0 Hz, 1H), 2.52 (s, 3H), 2.02–1.86 (m, 1H), 1.70–1.56 (m, 1H), 1.38–1.16 (m, 1H), 0.87 (t, J = 7.3 Hz, 3H). ^{13}C NMR (126 MHz, DMSO-d_6) δ 196.47, 168.45 (d, $J_{C\text{-}P}$ = 5.0 Hz), 143.64, 131.55, 129.41, 118.28, 47.84 (d, $J_{C\text{-}P}$ = 127 Hz), 28.97 (d, $J_{C\text{-}P}$ = 4.1 Hz), 26.40, 21.18 (d, $J_{C\text{-}P}$ = 15.5 Hz), 13.67. ^{31}P NMR (162 MHz, DMSO-d_6) δ 19.04. MS (ESI$^+$) m/z calculated for $C_{13}H_{19}NO_5P$ 300.10 [M+H]$^+$, found 300.04; HRMS (ESI$^-$) calculated for $C_{13}H_{17}NO_5P$ 298.0850 [M-H]$^-$, found 298.0836.

^{1}H NMR of compound 3g

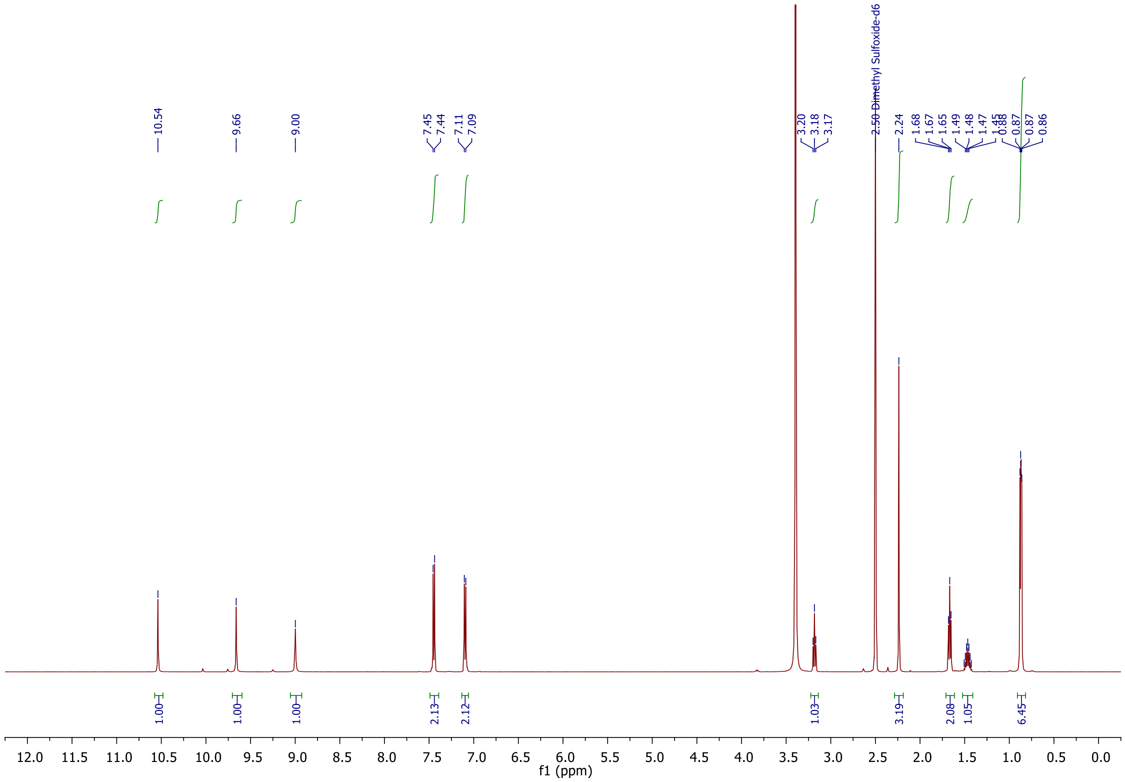

^{13}C NMR of compound 3g

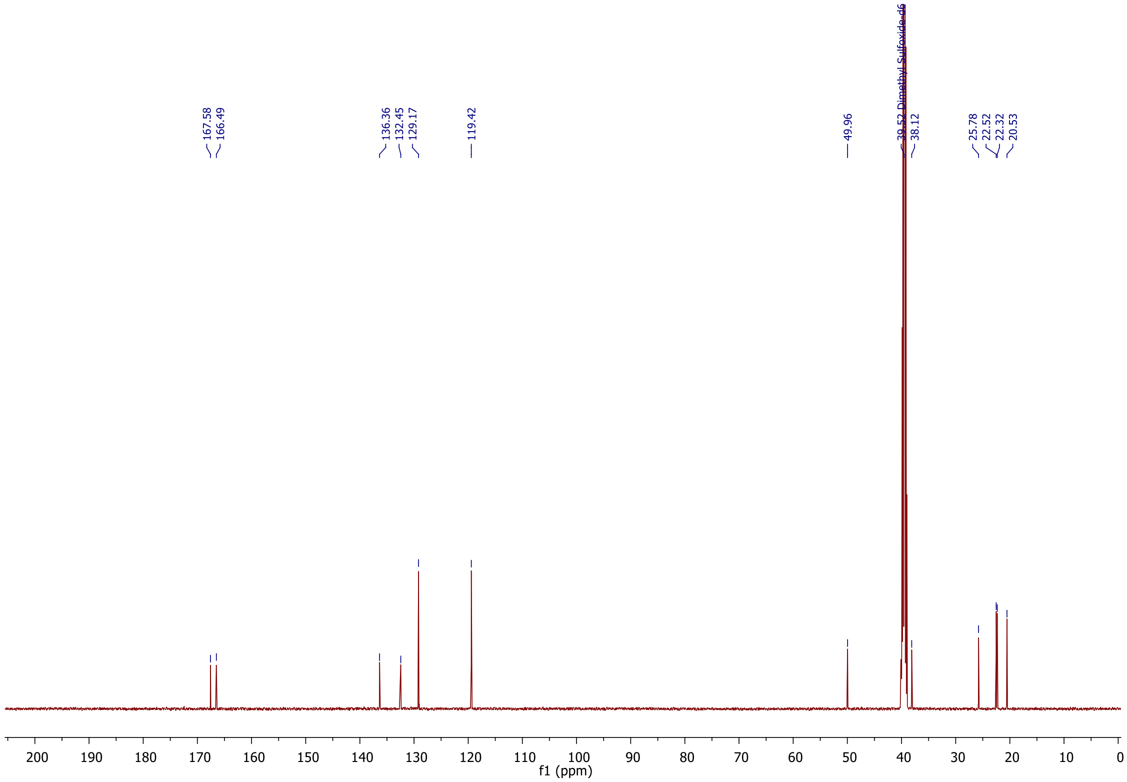

^{1}H NMR of compound 3i

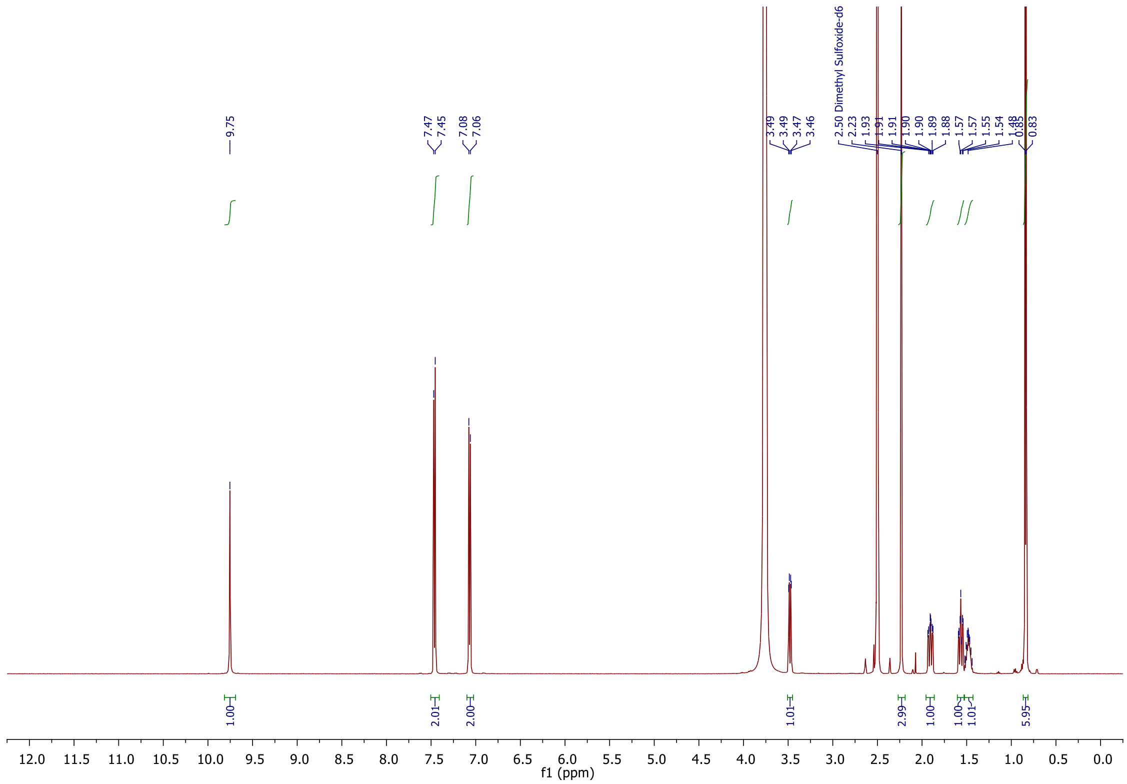

^{13}C NMR of compound 3i

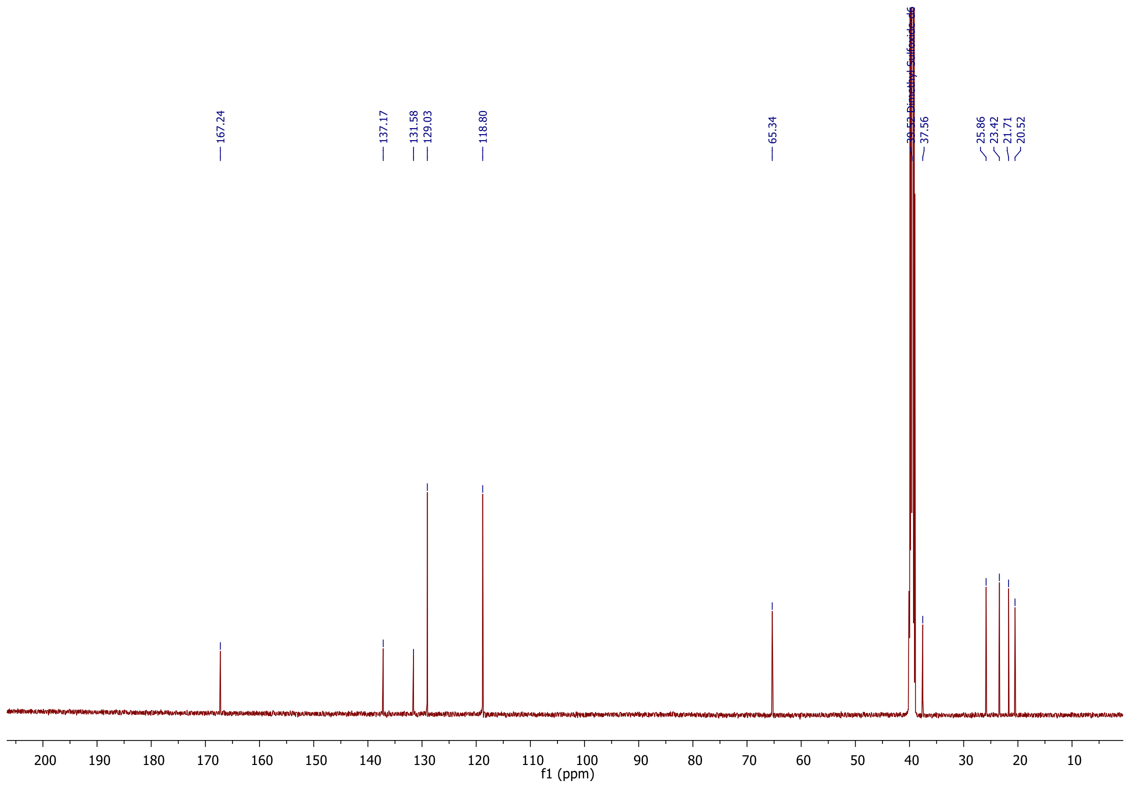

^{1}H NMR of compound 3l

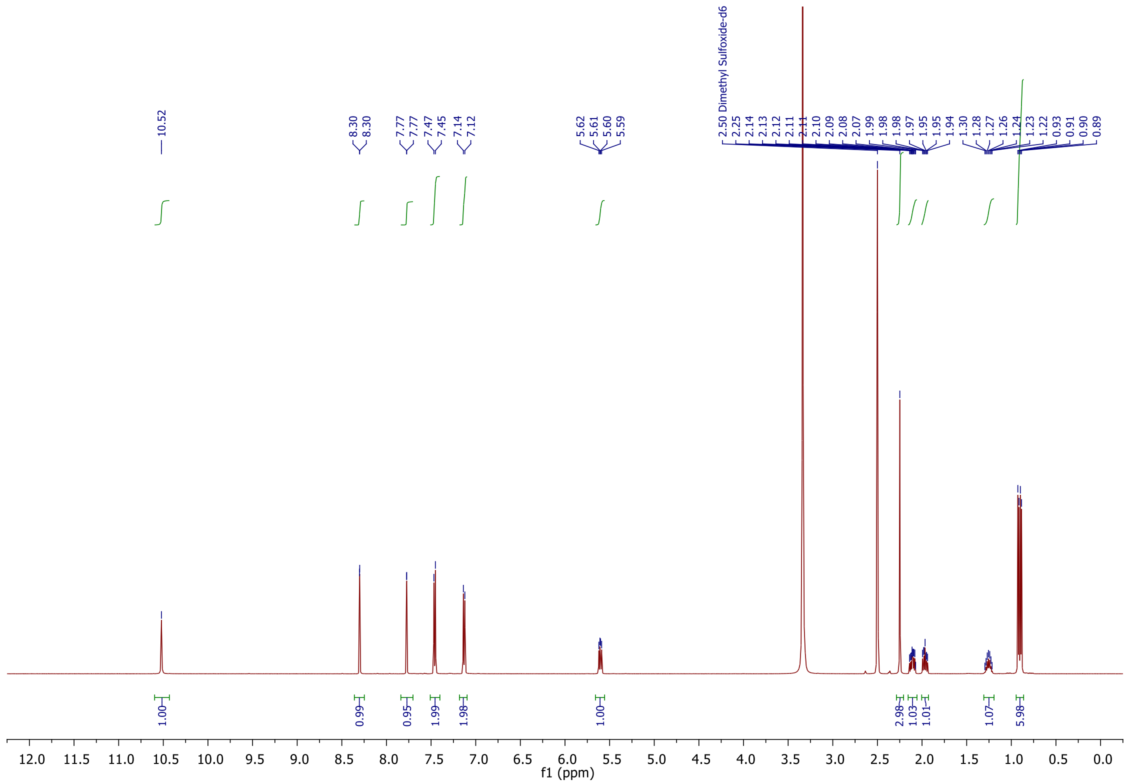

^{13}C NMR of compound 3l

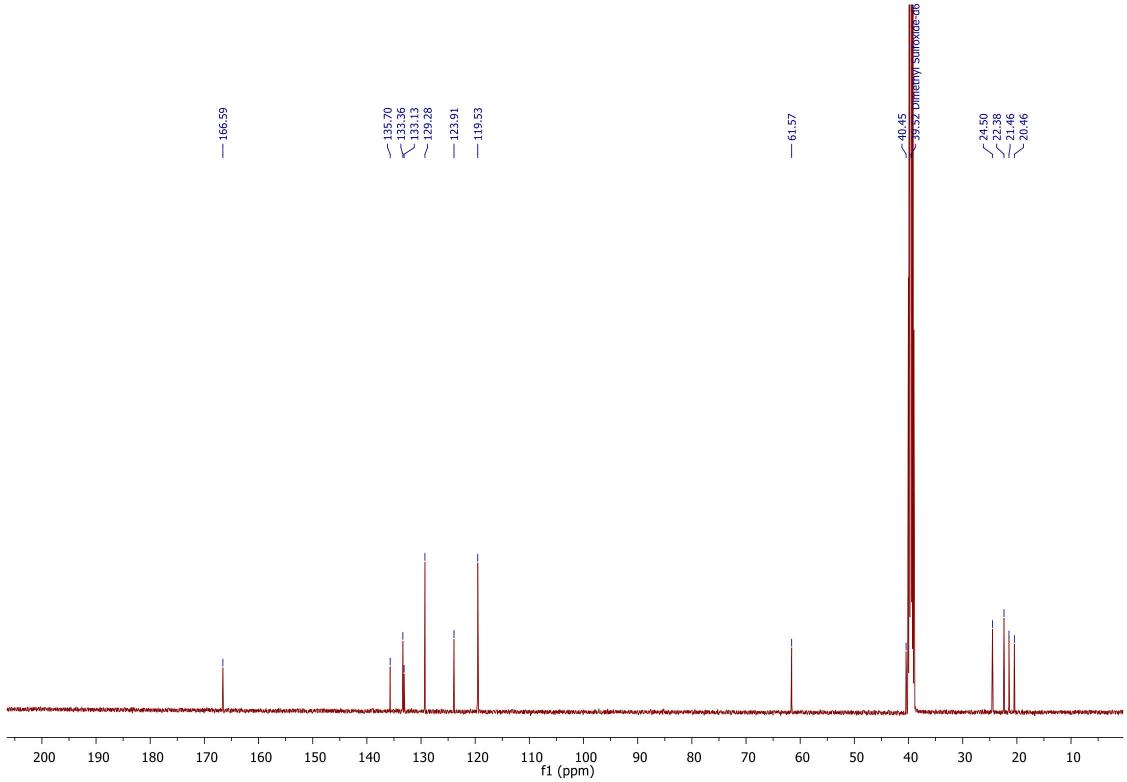

^{1}H NMR of compound 3m

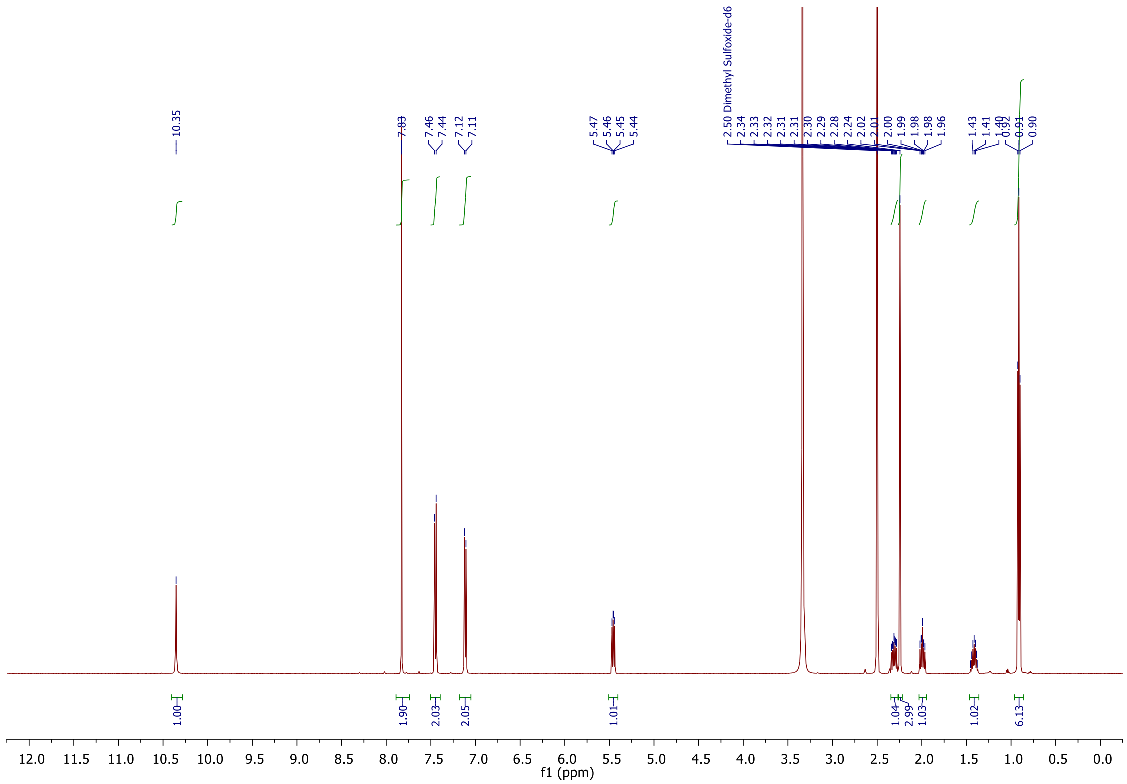

^{13}C NMR of compound 3m

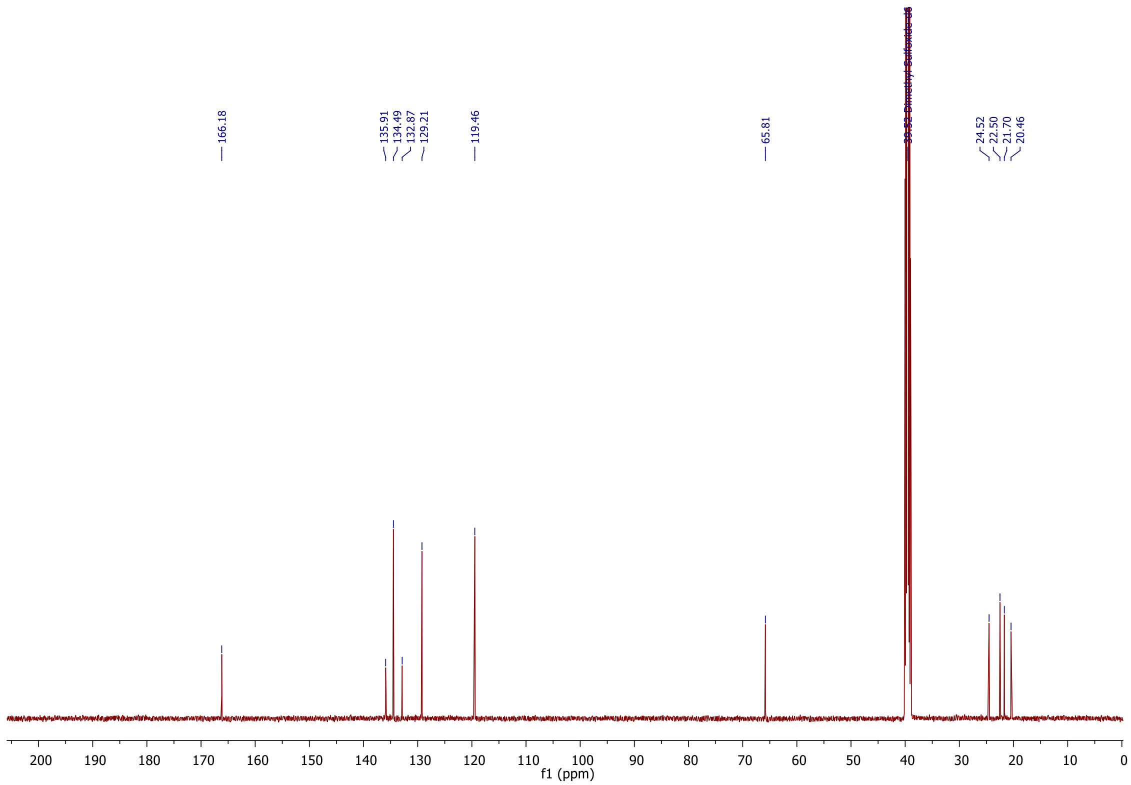

^{1}H NMR of compound 4a

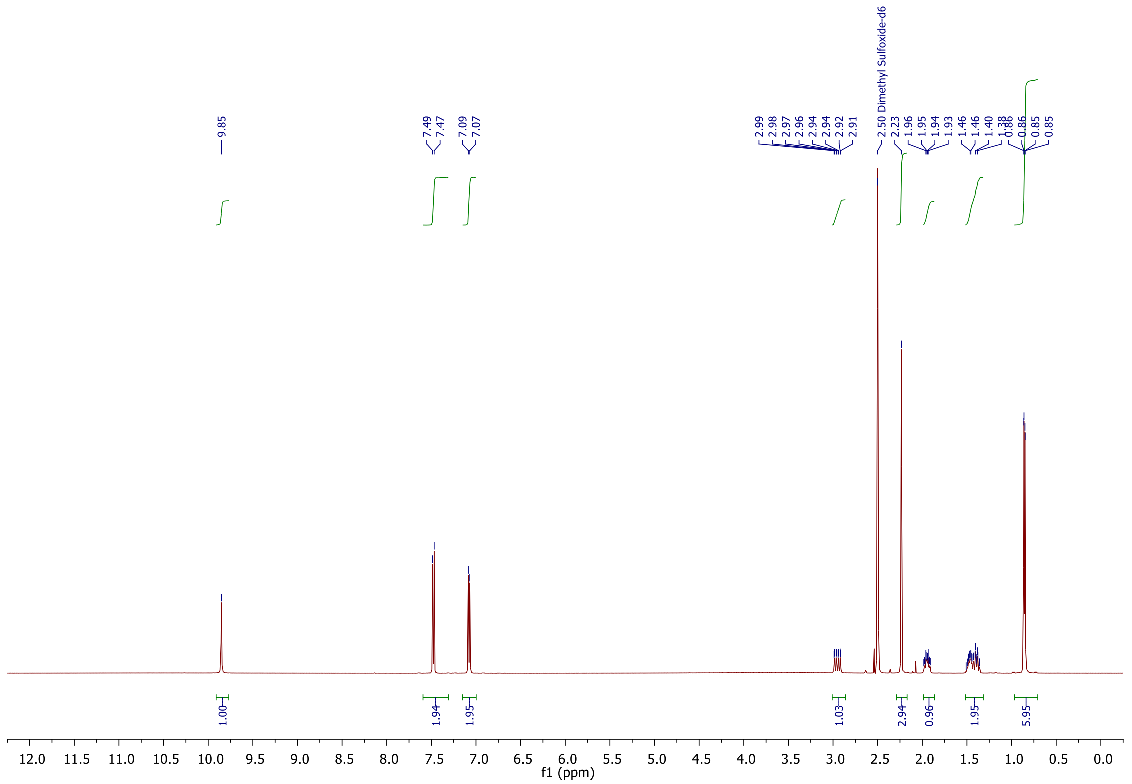

^{13}C NMR of compound 4a

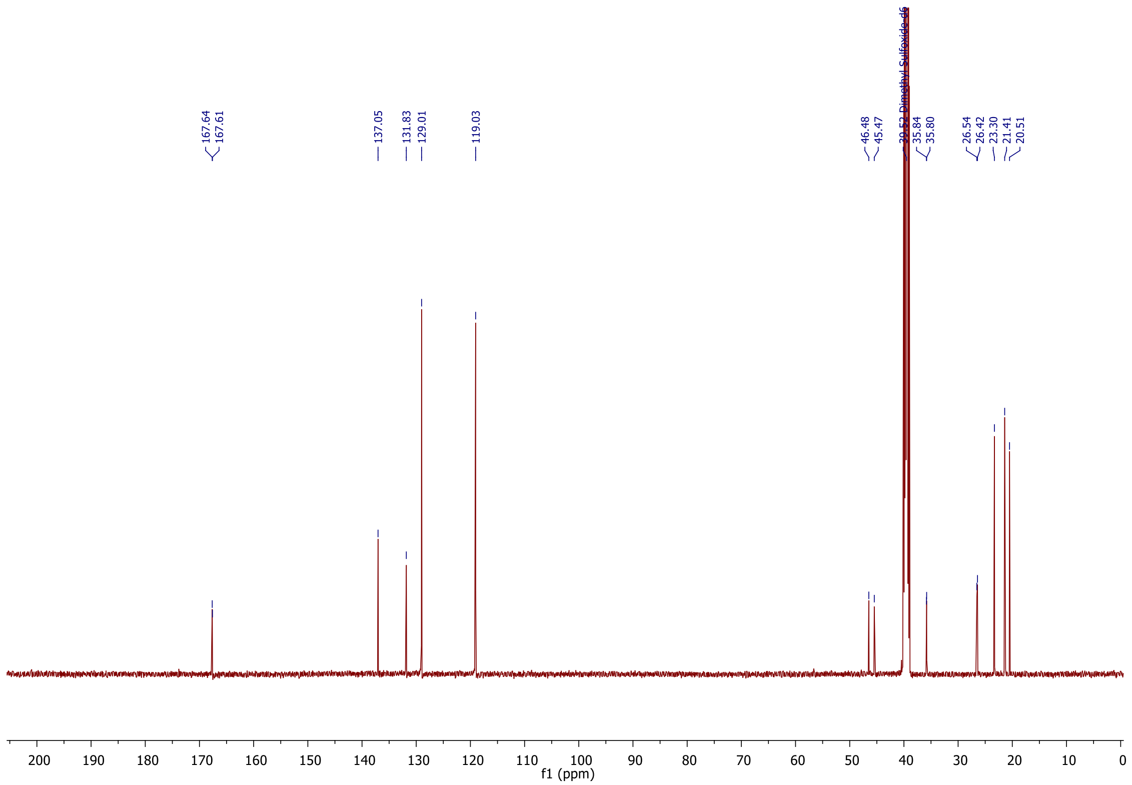

^{1}H NMR of compound 4b

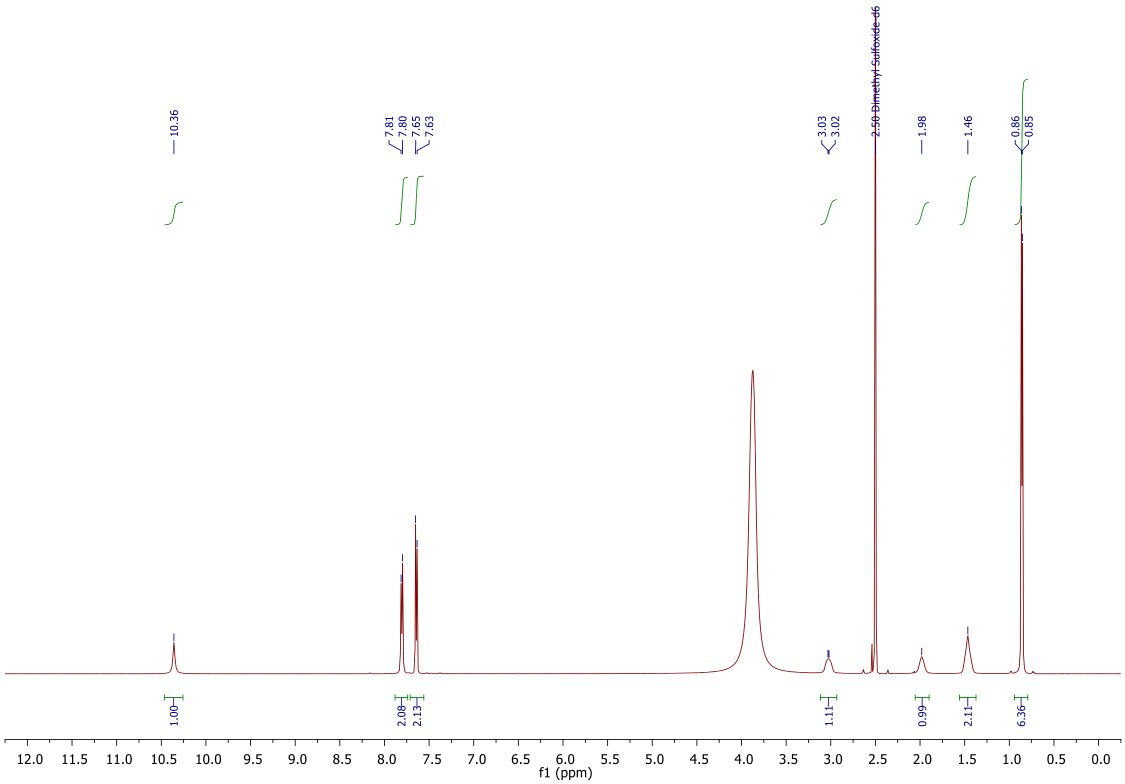

^{13}C NMR of compound 4b

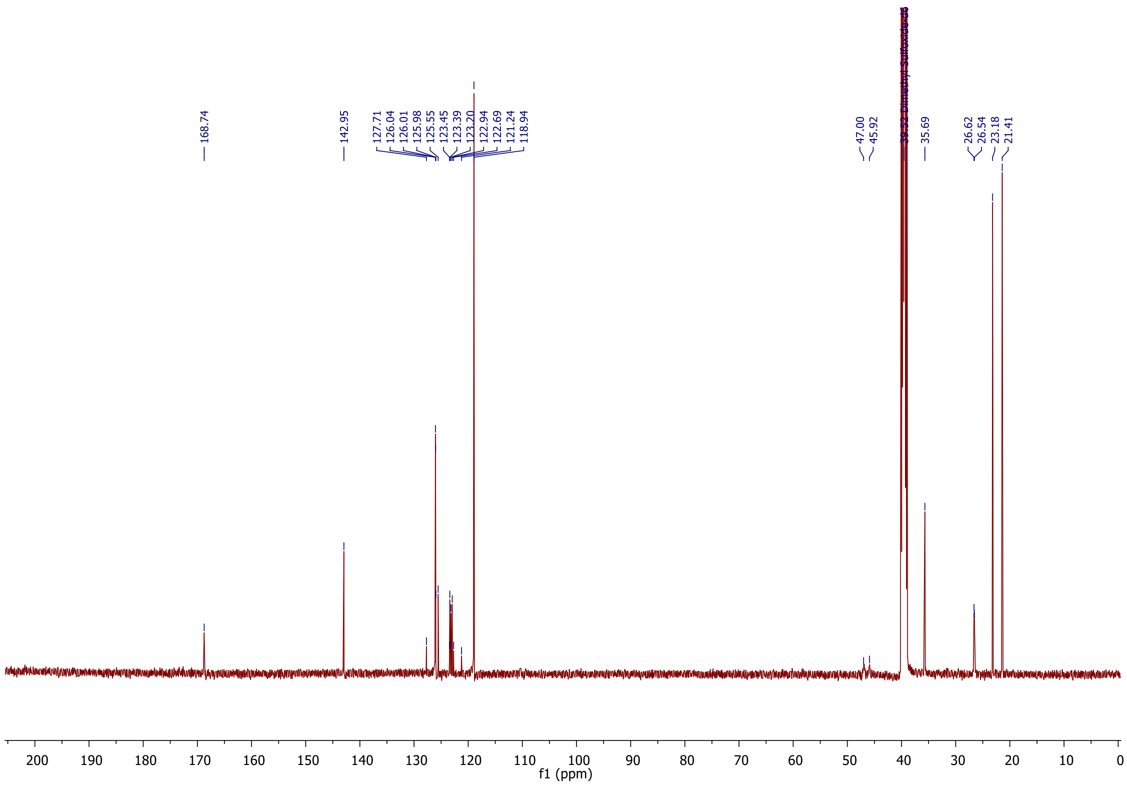

^{1}H NMR of compound 4h

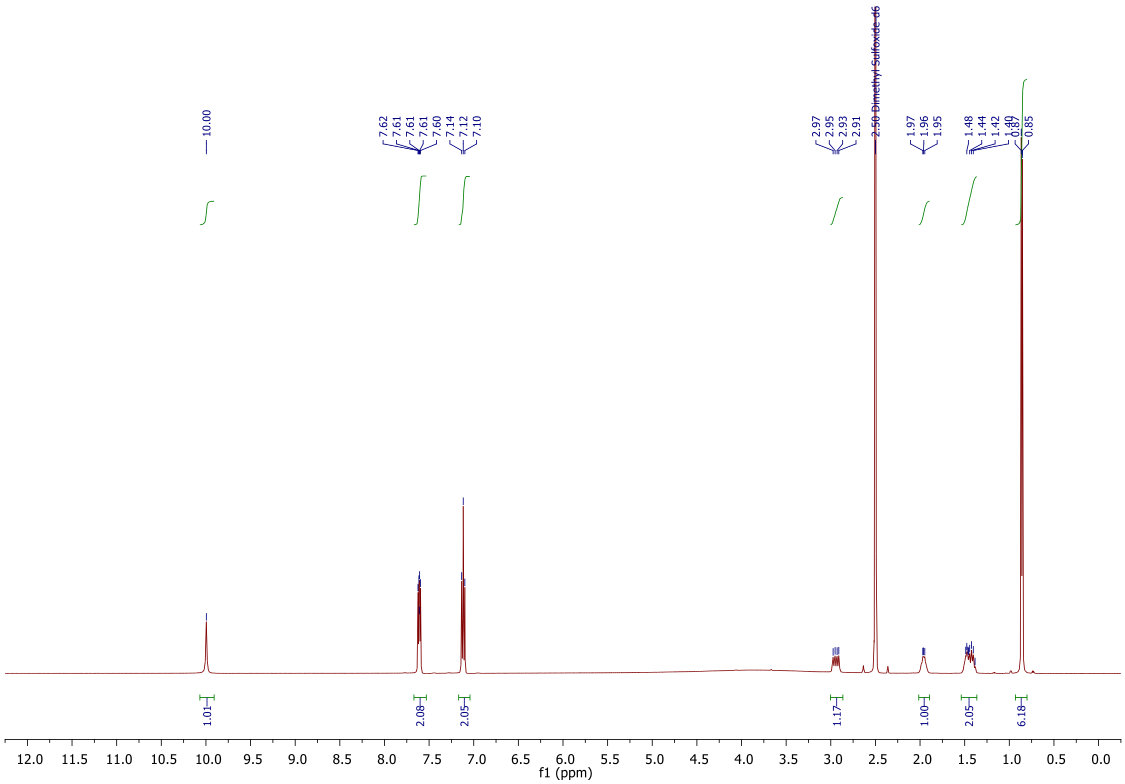

^{13}C NMR of compound 4h

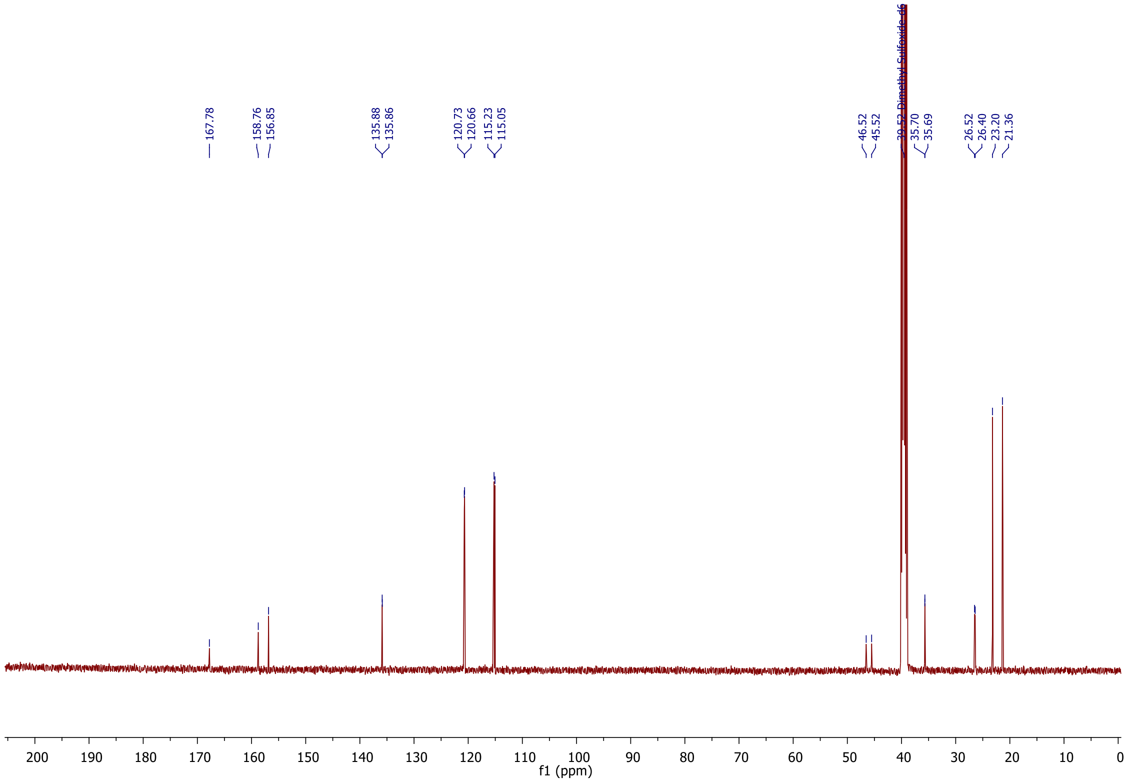

^{1}H NMR of compound 4k

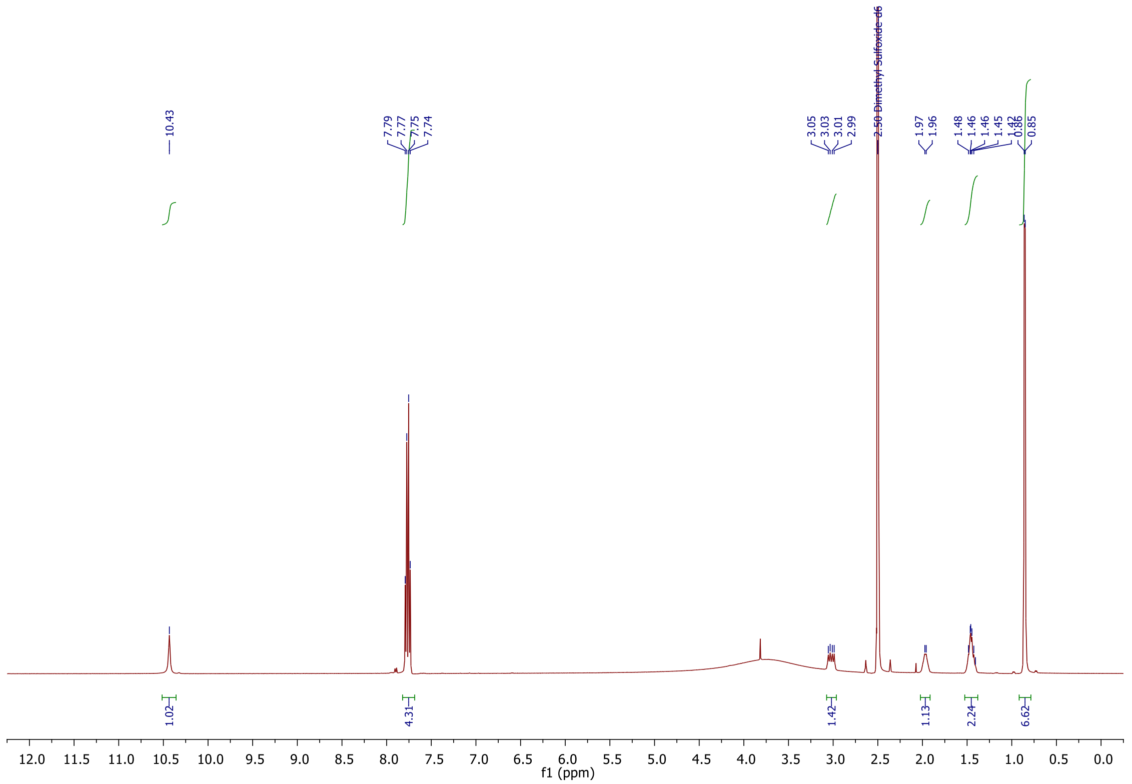

^{13}C NMR of compound 4k

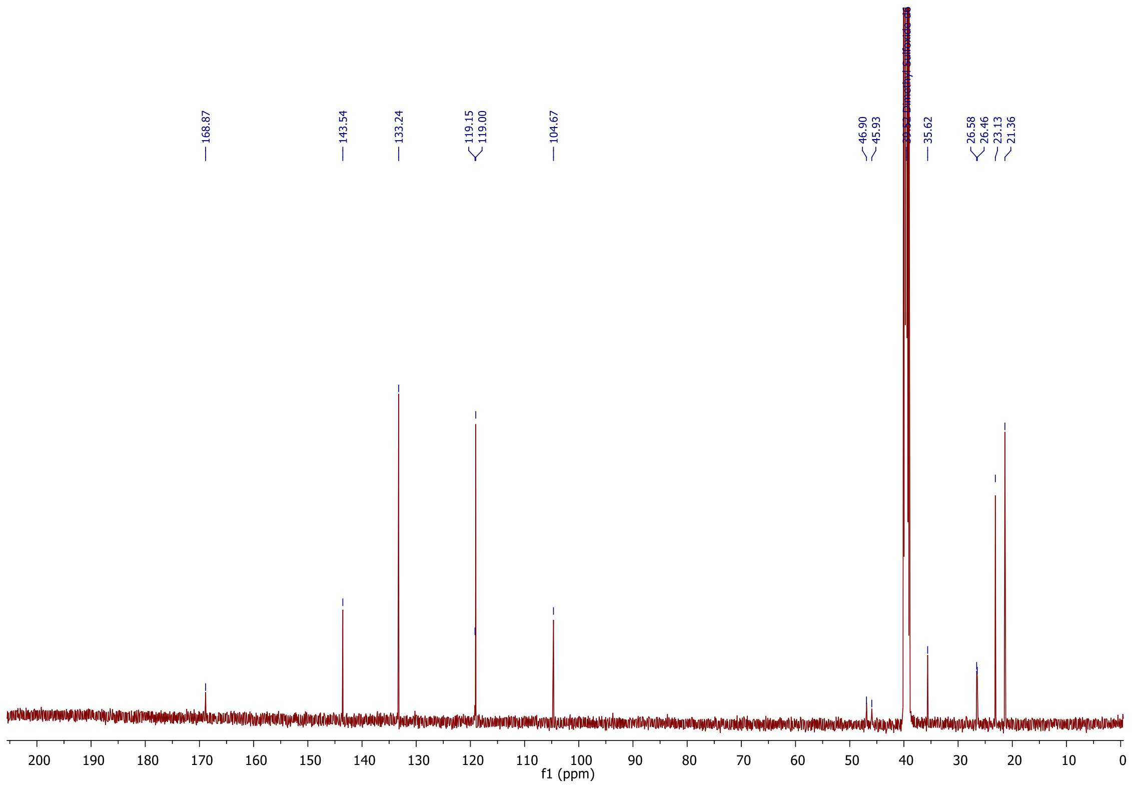

Materials and Methods

Kinetic turbidimetric solubility. The desired compounds were sequentially diluted in DMSO in a 96-well plate. 1.5 µL of each well were transferred into another 96-well plate and mixed with 148.5 µL of PBS. Plates were shaken for 5 min at 600 rpm at room temperature (r.t.), and the absorbance at 620 nm was measured. Absorbance values were normalized by blank subtraction and plotted using GraphPad Prism 8.4.2 (GraphPad Software, San Diego, CA, USA). Solubility (S) was determined based on the First X value of AUC function using a threshold of 0.005.

Lipophilicity determination. $LogD_{7.4}$ was analyzed using an HPLC−MS based method.[50] The retention time of 12 compounds with known $LogD_{7.4}$ was determined and plotted toward their $LogD_{7.4}$. Linear regression was used to determine the $LogD_{7.4}$ of unknown compounds. Analysis was performed using a Dionex Ultimate 3000 HPLC system coupled to a TSQ Quantum Access MAX (Thermo Fisher, Dreieich, Germany) with the following conditions: EC150/2 NUCLEODUR C18 Pyramid column, 5 µM (Macherey Nagel, Düren, Germany); eluent A: 50 mM NH_4OAc pH 7.4, eluent B: acetonitrile, and flow: 0.6 mL/min. The gradient was set to 0−100% B from 0 to 2.5 min, 100% B from 2.5 to 3.0 min, 100−0% B from 3.0 to 3.2 min, and 0% B from 3.2−5.0.

Metabolic stability. For the evaluation of combined phase I and phase II metabolic stability, the compound (1 µM) was incubated with 1 mg/mL pooled mouse liver S9 fraction (Xenotech, Kansas City, USA) or human liver S9 fraction (Corning, USA), 2 mM NADPH, 1 mM UDPGA, 10 mM $MgCl_2$, 5 mM GSH and 0.1 mM PAPS at 37 °C for 120 min. The metabolic stability of testosterone, verapamil and ketoconazole were determined in parallel to confirm the enzymatic activity of mouse S9 fractions, for human S9 testosterone, diclofenac and propranolol were used. The incubation was stopped after defined time points by precipitation of aliquots of S9 enzymes with 2 volumes of cold acetonitrile containing internal standard (150 nM diphenhydramine). Samples were stored on ice until the end of the incubation and precipitated protein was removed by centrifugation (15 min, 4 °C, 4,000 g). Concentration of the remaining test compound at the different time points was analyzed by HPLC-MS/MS (TSQ Quantum Access MAX, Thermo Fisher, Dreieich, Germany) and used to determine half-life ($t_{1/2}$) and intrinsic clearance (Cl_{int}).

Species profiling was conducted as above using 0.5 mg/mL pooled mouse, rat or minipig liver microsomes (Xenotech, Kansas City, USA) with 2 mM NADPH and 10 mM $MgCl_2$ and testosterone, verapamil and ketoconazole as reference compounds.

Plasma stability. To determine stability in plasma, a similar setup as for the determination of metabolic stability was applied using pooled mouse/human/rat or minipig plasma (Neo Biotech, Nanterre, France). Samples were taken by mixing aliquots with 4 volumes of acetonitrile containing internal standard (125 nM diphenhydramine). The plasma stability of procain, propantheline and diltiazem were determined in parallel to confirm the enzymatic activity.

Calu-3 permeability. Compound permeability was assessed *in vitro* with Calu-3 HTB-55 cell line (ATCC). Cells were cultivated in Minimum Essential Medium supplemented with Earle´s salts, L-glutamine, 10% FCS, 1% non-essential amino acids (NEAA) and 1mM sodium pyruvate. Passages between 35 and 55 were used, medium was changed every 2–3 days. For experiments, cells were harvested using trypsin/EDTA and 1x105 cells seeded on Transwell® inserts 3460. Cells were grown in air-liquid interface beginning day 3 and used for transport studies on day 11–13. Transepithelial/transendothelial electrical resistance (TEER) values exceeded 300 Ω*cm^2 before beginning transport studies. For experiments, Krebs-Ringer solution was used and cells were accommodated to the buffer for at least 1 h with no decrease in TEER. 200 µL samples were taken in regular intervals from the apical side (time intervals 0, 15, 30, 60, 120, 180, 300 min) and replenished with fresh buffer. TEER was monitored during the experiment, and epithelial barriers were considered compromised if the TEER fell below 300 Ω*cm^2 during 5 h of experiment duration. A cassette of atenolol, ciprofloxacin and carbamazepine was used as control. Test compounds were applied individually or in cassettes including up to three compounds.

Before analysis, 40 µL of sample was mixed with 80 µL of ice-cold acetonitrile containing internal standard diphenhydramine (150 nM), and the compound concentration was analyzed with HPLC-MS/MS (TSQ Quantum Access MAX, Thermo Fisher, Dreieich, Germany). Samples from the apical compartment taken at the beginning and end of the experiment were diluted 1:10 in KRB + 0.5% DMSO before mixing with acetonitrile.

Culturing of HepG2, A549 and HEK293 cells. The human hepatocellular carcinoma cell line HepG2, the lung adenocarcinoma cell line A549 and Human Embryonic (HEK) 293 cells were cultured in Dulbecco's Modified Eagle Medium (DMEM), containing 10% Fetal Bovine Serum

(FBS) and 1% penicillin-streptomycin mixture. Cells were maintained according to standard cell culture procedures.

Cytotoxicity assay. An MTT-based assay was employed to evaluate the viability of HepG2, HEK293 and A549 cells after challenge with selected LasB inhibitors and performed as described previously.[51]

Expression and purification of LasB and ColH-PD. LasB and ColH-PD were expressed and purified as described previously.[30,52]

***In vitro* inhibition assays (LasB, ColH, MMPs, TACE, HDACs).** All *in vitro* inhibition assays were performed as described previously.[30,53] TACE and HDAC inhibitor screening kits were purchased from Sigma-Aldrich (Saint Louis, MO). MMPs along with the SensoLyte 520 Generic MMP Activity Kit Fluorimetric were purchased from AnaSpec (Fremont, CA, USA), and the fluorometric cyclooxygenase 1 (COX1) inhibitor assay kit was purchased from Abcam (Cambridge, UK). The assays were performed according to the guidelines of the respective manufacturer. Fluorescence signals were measured using a CLARIOstar plate reader (BMG Labtech, Ortenberg, Germany). Pulmonary surfactant (poractant alfa), which is an extract of natural porcine lung surfactant, was purchased from Creative BioMart (Shirley, NY, USA).

PA14 csn production. The csn of wt PA14 and Δ*lasB* PA14 was produced as we previously reported.[31]

Validation of the effect of LasB inhibitors on A549 cells *in vitro*. The experiment was performed as described previously.[32] The Picrosirius red assay was performed by washing the csn-challenged cells (± inhibitor) 3x with PBS followed by an incubation with Bouin solution (Sigma) at RT for 20 min. The cells were incubated with 0.1% Picrosirius red dye (ab150681) at RT for 2 h. Then, they were washed once with 0.01 N HCl, and the matrix was dissolved in 0.01 N NaOH. The absorption was measured at 570 nm using a PHERAstar plate reader (BMG Labtech). By dividing the absorbance of each sample by the absorbance of the healthy sample, the relative collagen quantity was determined. For each condition, the experiment was performed three times. To determine the rescue of A549 cells treated with 5% wt PA14 csn using our compounds, the LasB inhibitors were used at 2, 0.5, 0.1 and 0.05 µM. The compounds were incubated in F12K medium, then deposited on the cells for 24 h. Cell detachment was then evaluated, and a Trypan Blue cell count was determined. Based on these results, IC_{50} values were determined.

To investigate the effect of **4a** and **4b** on the viability of A549 cells after challenge with culture supernatant (csn) of *Pseudomonas aeruginosa* PAO1 (DSM 22644, ATCC 15692), $2.5*10^3$ cells/well were seeded into a flat bottom 96-well plate (Corning™ Costar™) and incubated at 37 °C + 5% CO_2 for 24 h. On the following day, several concentrations of the compounds **4a** and **4b** were tested against 10% (v/v) *P. aeruginosa* PAO1 csn diluted in DMEM starting from 10 µM and 20 µM, respectively. Compound was initially dissolved in DMSO, and a low final assay concentration of 0.5% (v/v) was applied to minimize the proteolytic effect of DMSO on the bacterial culture supernatant. In addition, cells were challenged with 10% (v/v) of Δ*lasB* PAO1 to confirm the cytotoxic effect derived from LasB, and DMEM was included as a control without any treatment. Plates were incubated at 37 °C + 5% CO_2 for 24 h prior to the MTT assay (described above). Finally, data were statistically analyzed and graphically displayed using GraphPad Prism 9.

Comparison of the activity of LasB in culture supernatants of PAO1 and PA14 using a FRET-based proteolytic assay. Bacterial culture supernatant of *P. aeruginosa* strains PAO1 and PA14 was prepared from overnight cultures in lysogeny broth medium (LB). The cultures were centrifuged after approx. 18 hours and filtered using 0.2 µm non-pyrogenic sterile filters. The fluorogenic substrate 2-Aminobenzoyl-Ala-Gly-Leu-Ala-4-Nitrobenzylamide was supplied from Peptides International (Louisville, KY, USA) for use in this study. Fluorescence intensity was measured for 30 min at 37 °C using a CLARIOstar microplate reader (BMG Labtech, Ortenberg, Germany), with excitation and emission wavelengths of 340 ± 15 nm and 415 ± 20 nm, respectively, in black 384-well microtiter plates (Greiner Bio-One, Kremsmünster, Austria). The experiment was carried out in a final volume of 50 µL, containing assay buffer (50 mM Tris, pH 7.2, 2.5 mM CaCl2, 0.075% Pluronic F-127, 5% DMSO), PA14 and PAO1 supernatant at a final dilution factor of 5-5, and the substrate at a final concentration of 150 µM. Each sample was included in the multi-well plate in duplicates, and controls without supernatant were included for blank correction. After blank subtraction, the FRET signal of samples containing supernatant at different time points (every two minutes) was plotted using GraphPad Prism 9 (Graph Pad Software, San Diego, CA, USA).

Lung organoid assay. Primary basal cells were isolated as described previously.[54] The protocol was approved by the institutional review board of the Landesärztekammer of the State of Saarland. Informed consent was obtained from the patients. Basal cells were differentiated to 3D

bronchospheres as described before.[42] In brief, 80 µL of passage 1 basal cells (3 × 104 cells per mL differentiation media) were plated in each well of a 96-well plate containing 40 µL of a 25% Matrigel solution (Corning, USA). 120 µL differentiation media were added to the top every 4 days. At day 21, 120 µL media were removed from the top, and 120 µL differentiation media containing indicated concentrations of inhibitors were added. After 2 h, 120 µL media were removed, and 120 µL differentiation media with indicated concentrations of inhibitors with/without PA14 supernatants were added. After 48 h, 120 µL media were removed, and the MTT assay was performed following the manufacturer's instructions (MTT Cell Viability Assay Kit, biotium USA).

***G. mellonella* infection model.** The infection model was performed as described before with minor modifications (each larva was injected with 4 bacteria).[30] Injections were carried out using an LA120 syringe pump (Landgraf Laborsysteme, Langenhagen, Germany) equipped with 1 mL Injekt-F tuberculin syringes (B. Braun, Melsungen, Germany) and Sterican 0.30 × 12 mm2, 30 G × 1.5 needles (B. Braun). Larvae were incubated at 37 °C for 3 days and inspected twice daily. The total larvae used in all three experiments were 30 larvae per group. When the larvae became black and did not move when simulated with a tweezer, they were deemed dead.

Zebrafish embryo toxicity. The experiment was performed according to a procedure described in the literature[55] with minor modifications using zebrafish embryos of the AB wild-type line at 1 day post fertilization (dpf). A detailed protocol has been given in our recent publication.[33]

Pharmacokinetic (PK) studies in mice.

Mice. For PK experiments, outbred male CD-1 mice (Charles River, Germany), 4 weeks old, were used. The animal studies were conducted in accordance with the recommendations of the European Community (Directive 86/609/EEC, 24 November 1986). All animal procedures were performed in strict accordance with the German regulations of the Society for Laboratory Animal Science (GV- SOLAS) and the European Health Law of the Federation of Laboratory Animal Science Associations (FELASA). Animals were excluded from further analysis, if sacrifice was necessary according to the human endpoints established by the ethical board. All experiments were approved by the ethical board of the Niedersächsisches Landesamt für Verbraucherschutz und Lebensmittelsicherheit, Oldenburg, Germany.

PK studies. Compounds **3g**, **3i**, **3l**, **4a** and **4b** were dissolved in 2 % DMSO, 1 % Tyloxapol and 97 % PBS. The compounds were administered intratracheally (IT) at 0.25 mg/kg per compound as

cassette. Up to 5 compounds were dosed per cassette. Before administration, mice were anesthetized using ketamine 100 mg/kg and xylazine 10 mg/kg intraperitoneally. Mice (n=3 per time point) were euthanized at t= 0.5, 2 and 5 h post administration. **4b** was administered at 10 mg/kg using a Kent Scientific Aeroneb® nebulizer. Mice (n=3 per time point) were euthanized at t= 0.25, 0.5, 1, 2, 4, 8 and 24 h post administration. Blood was collected from the heart. Whole blood was collected into Eppendorf tubes coated with 0.5 M EDTA and immediately spun down at 13,000 rpm for 10 min at 4 °C. The plasma was transferred into a new Eppendorf tube and then stored at –80 °C until analysis. Then, a broncheoalveolar lavage was conducted using isotonic sodium chloride solution. Lung, kidneys, and liver were aseptically removed and homogenized using a Polytron tissue homogenizer (Kinematica) in isotonic sodium chloride solution. Organ samples were aliquoted into Eppendorf tubes and stored at –80 °C until analysis. Moreover, spontaneous urine was also collected. *Bioanalytical sample preparation.* All PK plasma samples were analyzed via HPLC-MS/MS using an Agilent 1290 Infinity II HPLC system and coupled to an AB Sciex QTrap6500+ mass spectrometer. First, a calibration curve was prepared by spiking different concentrations of **3g**, **3i**, **3l**, **4a** and **4b** into the respective matrix (mouse plasma (pooled, from CD-1 mice) for plasma samples, isotonic sodium chloride solution for BALF samples, lung tissue for lung samples, kidney tissue for kidney samples, liver tissue for liver samples and urine for urine samples). Caffeine was used as an internal standard. In addition, quality control samples (QCs) were prepared for **3g**, **3i**, **3l**, **4a** and **4b** with the respective matrix. The following extraction procedure was used: 7.5 µL of a plasma sample (calibration samples, QCs or PK samples) was extracted with 37.5 µL of methanol containing 12.5 ng/mL of caffeine as internal standard for 5 min at 2,000 rpm on an Eppendorf MixMate® vortex mixer. 10 µL of a urine sample (calibration samples, QCs or PK samples) was extracted with 40 µL of methanol containing 12.5 ng/mL of caffeine as internal standard for 5 min at 2000 rpm on an Eppendorf MixMate® vortex mixer. Then samples (plasma and urine) were spun down at 13,000 rpm for 5 min. Supernatants were transferred to standard HPLC-glass vials. 50 µL of a BALF / lung tissue / kidney tissue or liver tissue sample (calibration samples, QCs or PK samples) were extracted with 50 µL of methanol and 1 µL caffeine (concentration 1 µg/mL in methanol) for 5 min at 800 rpm on an Eppendorf MixMate® vortex mixer. Then samples (BALF, liver, kidney, lung) were spun down at 4,000 rpm for 40 min at 4 °C. Supernatants were transferred to 96well V-bottom plates (Greiner). HPLC conditions were as follows: column: Agilent Zorbax Eclipse Plus C18, 50x2.1 mm, 1.8 µm;

temperature: 30 °C; injection volume: 5 µL; flow rate: 700 µL/min; solvent A: water + 0.1% formic acid; solvent B: acetonitrile + 0.1% formic acid; gradient: 99% A at 0 min and until 1 min, 99% – 0% A from 1.0 min to 2.2 min, 0% A until 4 min. Mass spectrometric conditions were as follows: Scan type: MRM, negative and positive mode; Q1 and Q3 masses for caffeine, urea, **3g**, **3i**, **3l**, **4a** and **4b** can be found in table given below. Urea was used to enable calculation of epithelial lining fluid (ELF) concentrations. Peak areas of each sample and of the corresponding internal standard were analyzed using MultiQuant 3.0 software (AB Sciex). Peak areas of the respective sample were normalized to the internal standard peak area. The MS/MS pairs used for quantification are marked with a 'Q' in the table, the other MS/MS pairs for the respective compound were used for qualification. Peaks of PK samples were quantified using the calibration curve. The accuracy of the calibration curve was determined using QCs independently prepared on different days. PK parameters were determined using a non-compartmental analysis with PKSolver.[56] ELF concentrations were calculated using the following formula:[57]

$$(1)\ V_{ELF} = V_{BALF} \times \frac{Urea_{BALF}}{Urea_{Plasma}}$$

$$(2)\ c_{ELF} = c_{BALF} \times \frac{V_{BALF}}{V_{ELF}}$$

Q1 and Q3 masses for caffeine, urea, **3g**, **3i**, **3l**, **4a** and **4b (**MS/MS pairs used for quantification are marked with a 'Q')

ID	Q1 Mass [Da]	Q3 Mass [Da]	time [msec]	DP [volts]	CE [volts]	CXP [volts]
**Urea**	60.915	43.8 (Q)	30.0	56.0	17.0	16.0
		43.1	30.0	56.0	53.0	12.0
		29.1	30.0	56.0	111.0	6.0
**Caffeine**	195.024	138.000 (Q)	30.0	130.0	25.0	14.0
		110.000	30.0	130.0	31.0	18.0
**3g**	262.973	155.9	30.0	–75.0	–18.0	–13.0
		130.0	30.0	–75.0	–18.0	–13.0
**3i**	283.923	79.9 (Q)	30.0	–110.0	–66.0	–35.0
		106.0	30.0	–110.0	–32.0	–11.0
**3l**	270.973	131.0 (Q)	30.0	–65.0	–24.0	–7.0
		68.0	30.0	–65.0	–22.0	–13.0
**4a**	283.92	78.8 (Q)	30.0	–75.0	–22.0	–19.0
		63.0	30.0	–75.0	–130.0	–27.0
**4b**	337.906	78.9 (Q)	30.0	–125.0	–22.0	–9.0
		239.9	30.0	–125.0	–20.0	–11.0

Pharmacodynamic (PD) study with 4b and *P. aeruginosa*. For the therapeutic neutropenic pneumonia model, female CD-1 mice, 8-weeks-old, (Charles River, Germany) were rendered neutropenic by administration of 150 mg/kg and 100 mg/kg cyclophosphamide i.p. on day –4 and –1, respectively. The inoculum was prepared as follows: on day -1 the *P. aeruginosa* strain DSM-1117 (=ATCC 27853) was streaked out onto a blood agar plate and incubated at 37 °C. Then one single colony was inoculated into LB medium (diluted 1:6 in water) containing 0.01% mucin and incubated at 120 rpm and 37 °C. On day 0 bacteria were centrifuged for 15 min at 4,000 rpm and washed twice in 0.9 % NaCl-solution. Then they were adjusted to an OD of 10. For infection with *P. aeruginosa* an inoculum of $1.2x10^9$ cfu/mL was used. At d0 mice were infected by nebulization using an Aeroneb® nebulizer (Kent Scientific) of 20 µL $1.2x10^9$ CFU/ml *P. aeruginosa* DSM-1117. To control bacterial burden after nebulization, two animals were used as inoculum control group. The following groups were used: (1) vehicle control group, (2) levofloxacin group with 25 mg/kg IP QD (t= 2 h p.i.); (3) **4b** group with 10 mg/kg inhaled (t= 2, 6 and 10 h p.i.); (4) **4b** 10 mg/kg inhaled TID (t= 2, 6 and 10 h) combined with levofloxacin at 25 mg/kg IP QD (t= 2 h p.i.). After 24 h p.i., mice were euthanized for terminal analysis. After isolation of blood, lung was removed, weighed, and homogenized in 3 mL 0.9% NaCl. For determination of CFUs, suspensions of homogenized organs were serially diluted, plated on agar plates and incubated overnight at 37 °C. CFUs were determined by manual counting.

LC-MS/MS assay for determination of LasB in whole blood samples. All samples were analyzed via HPLC-MS/MS using an Agilent 1290 Infinity II HPLC system and coupled to an AB Sciex QTrap7500 mass spectrometer. Blood samples from the PD study with **4b** and P. aeruginosa were extracted as follows: 50 µL of a blood sample was extracted with 50 µL of acetonitrile for 5 min at 2,000 rpm on an Eppendorf MixMate® vortex mixer. Then samples were spun down at 13,000 rpm for 5 min. Supernatants were transferred to standard HPLC-glass vials. HPLC conditions were as follows: column: Agilent Zorbax Eclipse Plus C18, 50x2.1 mm, 1.8 µm; temperature: 30 °C; injection volume: 5 µL; flow rate: 700 µL/min; solvent A: water + 0.1% formic acid; solvent B: acetonitrile + 0.1% formic acid; gradient: 99% A at 0 min and until 1 min, 99% – 0% A from 1.0 min to 3 min, 0% A until 4.7 min. Mass spectrometric conditions were as follows: Scan type: MRM, positive mode; Source temperature: 500°C; Spray voltage: 2000 V; Q1 and Q3 masses for LasB can be found in the table below.

Q1 and Q3 masses for LasB protein fragments**;** EP in volts was set to 10 for every transition. Likewise, CXP in volts was set to 17.5. Dwell time was 3 msec for every transition.

ID	Peptide	Q1 mass [Da]	Q3 Mass [Da]	CE [V]
**LasB**	6FZX_1|Chain.C[CAM]EMDDGNVITVDMNSSTDDSK.+3y1.light	778.312	1099.421	35.4
**LasB**	6FZX_1|Chain.C[CAM]EMDDGNVITVDMNSSTDDSK.+3y9.light	778.312	984.394	35.4
**LasB**	6FZX_1|Chain.C[CAM]EMDDGNVITVDMNSSTDDSK.+3y8.light	778.312	853.353	35.4
**LasB**	6FZX_1|Chain.FAC[CAM]PTNTYK.+2y7.light	551.255	883.398	26
**LasB**	6FZX_1|Chain.FAC[CAM]PTNTYK.+2y6.light	551.255	723.367	26
**LasB**	6FZX_1|Chain.FAC[CAM]PTNTYK.+2y5.light	551.255	626.314	26
**LasB**	6FZX_1|Chain.FAC[CAM]PTNTYK.+3y6.light	367.839	723.367	15.7
**LasB**	6FZX_1|Chain.FAC[CAM]PTNTYK.+3y5.light	367.839	626.314	15.7
**LasB**	6FZX_1|Chain.FAC[CAM]PTNTYK.+3y4.light	367.839	525.267	15.7
**LasB**	6FZX_1|Chain.FAC[CAM]PTNTYK.+3y3.light	367.839	411.224	15.7
**LasB**	6FZX_1|Chain.QVNGAYSPLNDAHFFGGVVFK.+3y9.light	756.38	1037.557	34.3
**LasB**	6FZX_1|Chain.QVNGAYSPLNDAHFFGGVVFK.+3y8.light	756.38	900.498	34.3
**LasB**	6FZX_1|Chain.DWFGTSPLTHK.+2y8.light	644.82	840.457	30.6
**LasB**	6FZX_1|Chain.DWFGTSPLTHK.+2y7.light	644.82	783.436	30.6
**LasB**	6FZX_1|Chain.DWFGTSPLTHK.+2y6.light	644.82	682.388	30.6
**LasB**	6FZX_1|Chain.DWFGTSPLTHK.+2y5.light	644.82	595.356	30.6
**LasB**	6FZX_1|Chain.DWFGTSPLTHK.+3y6.light	430.216	682.388	18.7
**LasB**	6FZX_1|Chain.DWFGTSPLTHK.+3y5.light	430.216	595.356	18.7
**LasB**	6FZX_1|Chain.DWFGTSPLTHK.+3y4.light	430.216	498.303	18.7
**LasB**	6FZX_1|Chain.GQSGGMNEAFSDMAGEAAEFYMR.+3y9.light	819.337	1073.472	37.3
**LasB**	6FZX_1|Chain.GQSGGMNEAFSDMAGEAAEFYMR.+3y8.light	819.337	1016.451	37.3
**LasB**	6FZX_1|Chain.GQSGGMNEAFSDMAGEAAEFYMR.+3y7.light	819.337	887.408	37.3
**LasB**	6FZX_1|Chain.NDFLIGYDIK.+2y8.light	599.311	968.545	28.4
**LasB**	6FZX_1|Chain.NDFLIGYDIK.+2y7.light	599.311	821.477	28.4
**LasB**	6FZX_1|Chain.NDFLIGYDIK.+2y6.light	599.311	708.393	28.4
**LasB**	6FZX_1|Chain.NDFLIGYDIK.+3y6.light	399.877	708.393	17.2
**LasB**	6FZX_1|Chain.NDFLIGYDIK.+3y5.light	399.877	595.309	17.2
**LasB**	6FZX_1|Chain.NDFLIGYDIK.+3y4.light	399.877	538.287	17.2
**LasB**	6FZX_1|Chain.YMDQPSR.+2y6.light	448.7	733.33	21
**LasB**	6FZX_1|Chain.YMDQPSR.+2y5.light	448.7	602.289	21
**LasB**	6FZX_1|Chain.YMDQPSR.+2y4.light	448.7	487.262	21
**LasB**	6FZX_1|Chain.YMDQPSR.+2y3.light	448.7	359.204	21
**LasB**	6FZX_1|Chain.YMDQPSR.+3y5.light	299.469	602.289	12.4
**LasB**	6FZX_1|Chain.YMDQPSR.+3y4.light	299.469	487.262	12.4
**LasB**	6FZX_1|Chain.YMDQPSR.+3y3.light	299.469	359.204	12.4
**LasB**	6FZX_1|Chain.SIDNASQYYNGIDVHHSSGVYNR.+3y10.light	866.065	1155.565	39.6
**LasB**	6FZX_1|Chain.SIDNASQYYNGIDVHHSSGVYNR.+3y9.light	866.065	1056.497	39.6
**LasB**	6FZX_1|Chain.SIDNASQYYNGIDVHHSSGVYNR.+3y8.light	866.065	919.438	39.6
**LasB**	6FZX_1|Chain.AFYLLANSPGWDTR.+2y9.light	805.902	1003.459	38.5
**LasB**	6FZX_1|Chain.AFYLLANSPGWDTR.+2y8.light	805.902	932.422	38.5
**LasB**	6FZX_1|Chain.AFYLLANSPGWDTR.+2y7.light	805.902	818.379	38.5
**LasB**	6FZX_1|Chain.AFYLLANSPGWDTR.+2y6.light	805.902	731.347	38.5
**LasB**	6FZX_1|Chain.AFYLLANSPGWDTR.+3y6.light	537.604	731.347	23.8
**LasB**	6FZX_1|Chain.AFYLLANSPGWDTR.+3y5.light	537.604	634.294	23.8
**LasB**	6FZX_1|Chain.AFYLLANSPGWDTR.+3y4.light	537.604	577.273	23.8
**LasB**	6FZX_1|Chain.AFEVFVDANR.+2y8.light	584.293	949.474	27.6

**LasB**	6FZX_1|Chain.AFEVFVDANR.+2y7.light	584.293	820.431	27.6
**LasB**	6FZX_1|Chain.AFEVFVDANR.+2y6.light	584.293	721.363	27.6
**LasB**	6FZX_1|Chain.AFEVFVDANR.+3y6.light	389.865	721.363	16.7
**LasB**	6FZX_1|Chain.AFEVFVDANR.+3y5.light	389.865	574.294	16.7
**LasB**	6FZX_1|Chain.AFEVFVDANR.+3y4.light	389.865	475.226	16.7
**LasB**	6FZX_1|Chain.YYWTATSNYNSGAC[CAM]GVIR.+2y10.light	1041.971	1096.52	50.1
**LasB**	6FZX_1|Chain.YYWTATSNYNSGAC[CAM]GVIR.+3y9.light	694.983	933.457	31.4
**LasB**	6FZX_1|Chain.YYWTATSNYNSGAC[CAM]GVIR.+3y8.light	694.983	819.414	31.4
**LasB**	6FZX_1|Chain.YYWTATSNYNSGAC[CAM]GVIR.+3y7.light	694.983	732.382	31.4
**LasB**	6FZX_1|Chain.NYSAADVTR.+2y7.light	498.741	719.368	23.4
**LasB**	6FZX_1|Chain.NYSAADVTR.+2y6.light	498.741	632.336	23.4
**LasB**	6FZX_1|Chain.NYSAADVTR.+2y5.light	498.741	561.299	23.4
**LasB**	6FZX_1|Chain.NYSAADVTR.+3y5.light	332.83	561.299	14
**LasB**	6FZX_1|Chain.NYSAADVTR.+3y4.light	332.83	490.262	14
**LasB**	6FZX_1|Chain.NYSAADVTR.+3y3.light	332.83	375.235	14
**LasB**	6FZX_1|Chain.AFSTVGVTC[CAM]PSAL.+2y9.light	655.326	903.46	31.1
**LasB**	6FZX_1|Chain.AFSTVGVTC[CAM]PSAL.+2y8.light	655.326	804.392	31.1
**LasB**	6FZX_1|Chain.AFSTVGVTC[CAM]PSAL.+2y7.light	655.326	747.371	31.1
**LasB**	6FZX_1|Chain.AFSTVGVTC[CAM]PSAL.+2y4.light	655.326	387.224	31.1
**LasB**	6FZX_1|Chain.AFSTVGVTC[CAM]PSAL.+3y7.light	437.22	747.371	19
**LasB**	6FZX_1|Chain.AFSTVGVTC[CAM]PSAL.+3y6.light	437.22	648.302	19
**LasB**	6FZX_1|Chain.AFSTVGVTC[CAM]PSAL.+3y5.light	437.22	547.254	19
**LasB**	6FZX_1|Chain.AFSTVGVTC[CAM]PSAL.+3y4.light	437.22	387.224	19

Bacterial growth inhibition assay. Assays regarding the determination of the MIC were performed as described recently for *P. aeruginosa* PA14.[58]

X-ray crystallography. LasB was expressed and purified as described previously.[30] The protein was concentrated to 12 mg/mL and mixed with compound **4b** at a final concentration of 1 mM. Complex crystals were obtained in 0.1 M Tris-Cl pH 8.5, and 1.6 ammonium sulfate. Crystals were cryoprotected in glycerol, and diffraction data was collected from single crystals at 100 K at beamline P11 at Petra III (DESY) at a wavelength of 0.967 Å.[59] Data were processed using Xia2 or XDS, and the structure solved using PHASER Molecular Replacement with *P. aeruginosa* elastase (PDB ID 1EZM) as a search model.[60,61,62,63] The models were manually rebuilt with COOT and refined using PHENIX and Refmac5.[63,64,65]

Supplementary Text

Synthesis of derivatives bearing different ZBGs. The synthesis of derivatives bearing different ZBGs is shown on Figures S1 and S2. Coupling of *N*-Boc protected L-leucine with *p*-toluidine using EDC·HCl as a coupling reagent gave compound **3a** in high yield. Boc-deprotection using trifluoroacetic acid in DCM afforded **3b** in moderate yield. 2-Hydroxy-4-methylpentanoic acid **S2**

under the same coupling conditions gave **S3**, which was used as a precursor for the sulfamate derivative **3c**. 2-(Ethoxycarbonyl)-4-methylpentanoic acid **S4**, obtained by mono-saponification of the α-isobutyl diethylmalonate served as a carboxylic acid partner in the coupling reaction to obtain **3d** in a good yield (70%). This ester derivative was subsequently used as a precursor to three other zinc binders. Hydrolysis with NaOH in EtOH/water mixture gave compound **3e** in excellent yield (97%). Reaction with hydroxylamine in presence of KCN in methanol afforded hydroxamic acid **3g** in moderate yield, while the amidation reaction with ammonia in methanol led to the amide derivative **3f** in 52% yield. Nucleophilic substitution of bromide in **S5a** using sodium cyanide in DMF/water mixture afforded cyano derivative **3h** in a good yield. This was followed by a click reaction using sodium azide and zinc(II)-bromide in iPrOH/water mixture at 120 °C, to afford tetrazole derivative **3j** in 14% yield. Another nucleophilic substitution of bromide **S5a** with sodium sulfite in ethanol/water mixture at 80 °C yielded sulfonic acid derivative **3i** (42%). The imidazole derivative **3k** and three triazole derivatives **3l–3n** were obtained in low to moderate yield using potassium carbonate in acetone at 70 °C and 1*H*-imidazole, 1*H*-1,2,3-triazole or 1*H*-1,2,4-triazole, respectively.

Synthesis of phosphonic acid derivatives is shown on Figures S3–S5. Reaction of the carboxylic acids and corresponding anilines, using EDC·HCl as a coupling reagent, was followed by an Arbuzov reaction in presence of triethylphosphite at 150 °C. In the final step, cleavage of the diethylphosphonate esters using TMSBr afforded the final phosponic acid derivatives in low to moderate yield (29–73%). Synthesis of 6-membered nitrogen-containing heterocyclic derivatives followed the same reaction cascade as in the case of substituted phenyl derivatives (Figure S4). We used four nitrogen-containing anilines with chlorine substituent, affording four corresponding diethylphosphonate esters. As expected for 2-chloropyrimidin-5-yl- and 6-chloropyridazin-3-yl-derivatives (**S7t** and **S7u**), during the final step we noticed the formation of additional bromo-substituted derivatives (**4u** and **4w**, respectively). In addition, we investigated the effect of another two electron-withdrawing substituents, such as carboxamide and carboxymethyl, giving in total eight heterocyclic derivatives containing one or two nitrogen atoms and different substitution patterns on the aromatic core (Figure S4).

In addition to the modifications on the aromatic core, we synthesized seven derivatives with side-chain variations – two with α-benzyl (**4x** and **4y**), two with α-methylcyclohexyl (**4z** and **4aa**) and

three bearing a propyl substituent (**4ab–4ad**, Figure S5). Interestingly, similar compounds bearing an α-benzyl side chain have been already reported in literature as antiparasitic agents.[66]

ADMET profiling. Calu-3 cells have been extensively used as a model system for studying compounds intended for a delivery to the lungs. This human lung cancer cell line with an epithelial morphology expresses the proteins of major intercellular junctions and produces secretory components, therefore showing a high resemblance to the native airway epithelium.[67,68]

Phosphonic acid derivatives **4a** and **4b** and the sulfonic acid **3i** showed the lowest P_{app} values in Calu-3 cells, while the hydroxamic acid **3g** and triazole derivatives **3l** and **3m** proved to be several fold more permeable. In addition to the desired low lung permeability, phosphonic acids were not metabolized in mouse or human liver S9 fractions, while triazole derivatives **3l** and **3m** as well as hydroxamic acid **3g** turned out to be poorly stable in mouse liver S9 fractions. Sulfonic acid **3i** shows a similar profile compared to the phosphonates, but a significantly weaker activity, which is the reason why we favored the phosphonates.

Selectivity against human off-targets. We selected a panel of metzincs, including six human matrix metalloproteases (MMP-1, -2, -3, -7, -8, -14) and TACE (ADAM-17), as well as two HDACs (HDAC-3 and HDAC-8), all of which have key functions in physiological processes from degradation of the extracellular matrix components, regulation of the release of bioactive molecules essential for signaling networks, to control of the gene expression and modulation of the transcription.[69,70,71] In our previous work, we showed *N*-aryl-2-isobutylmercaptoacetamide **1** to be highly selective towards six MMPs and HDACs, yet demonstrating certain activity against TACE and COX-1.[29] Cyclooxygenase COX-1, an integral membrane protein involved in the protection of the gastric mucosa,[72] was found to be one of the problematic off-targets for **1** in the Safetyscreen44 and was therefore included in the panel evaluating the new inhibitors. In addition to already discussed phosphonic acid derivatives, the sulfonic acid **3i** and the two triazole derivatives (**3l** and **3m**) showed an excellent selectivity profile as well, but are, however, significantly less potent against LasB. On the other hand, the hydroxamic acid **3g**, although being 2-fold more potent against LasB compared to the best phosphonic inhibitor, shows slight effect on MMP-1, -2 and -3 and the inhibition of TACE in the micromolar range.

Activity against *Clostridium histolyticum* collagenase ColH. Selected compounds were evaluated for their inhibitory activity against ColH. In general, newly developed inhibitors possess no or weak inhibition of ColH-PD. The exception is the compound **4g**, which due to 4-acetyl

substituent, which is known to be beneficial for ColH activity, shows 65% of inhibition when tested at 1 µM. The K_i for this compound has been determined to be 0.32 ± 0.07 µM, which is a 20-fold higher potency compared to **2 (**IC_{50} = 7 ± 1 µM).

Off-Target Safety Screen44^{TM} Panel. The safety screen panel was performed by the CRO Eurofins Cerep, according to their guidelines. In each experiment and if applicable, the respective reference compound was tested concurrently with the test compounds, and the data were compared with historical values determined by Eurofins. The experiment was accepted in accordance with their Standard Operating Procedures.

Zebrafish embryo toxicity. This model has been widely used to evaluate the compound's toxicity, as it is offering numerous advantages, from the size of zebrafish embryos and easy handling to more important ones such as high fertility rate and transparency, allowing for detection of malformation during the development.[73] Moreover, zebrafish are showing a significant homology to the human genome, making them an excellent model system for determining the compound's toxicity profile.[74] Table S9 shows the results obtained in the study with **4a** and **4b**.

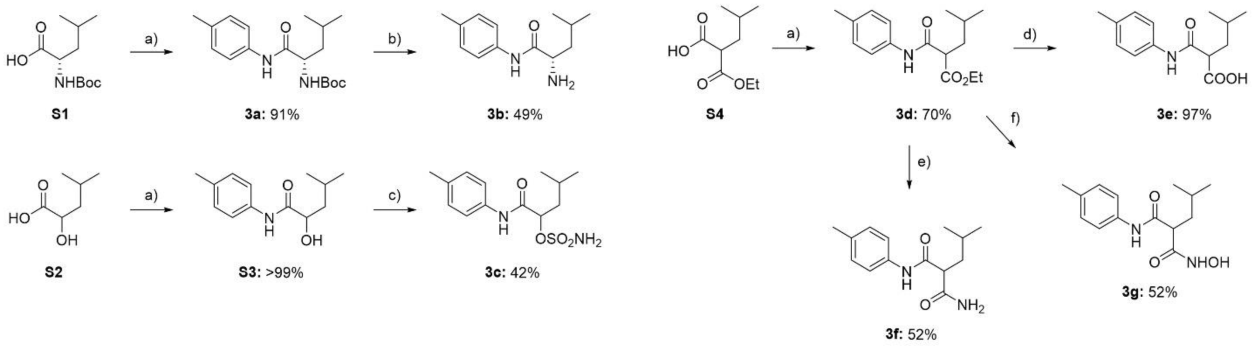

a) *p*-toluidine, EDC-HCl, DCM, r.t.; b) TFA, DCM, r.t.; c) SO_2ClNH_2, DMA, 0 ^oC to r.t.; d) NaOH, EtOH/H_2O, r.t.; e) NH_3; MeOH, r.t.; f) NH_2OH, KCN, MeOH, r.t.

Figure S1. Synthesis of derivatives bearing the most common zinc-binding groups (part I)

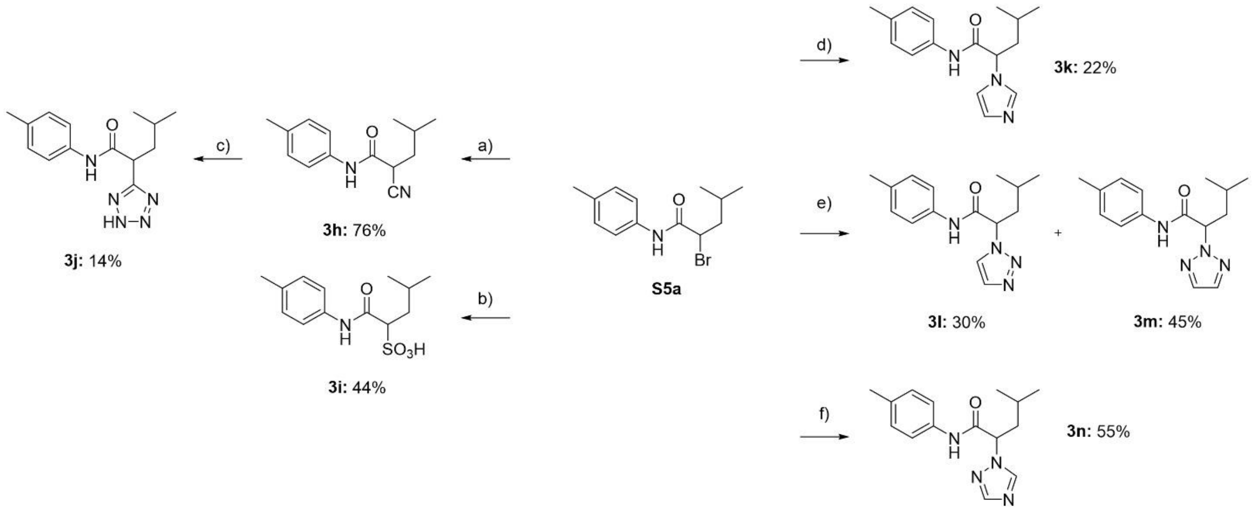

a) NaCN, DMF/H_2O, r.t.; b) Na_2SO_3, EtOH/H_2O, 80 ^oC; c) NaN_3, $ZnBr_2$, $^iPrOH/H_2O$, 120 ^oC; d) 1*H*-imidazole, K_2CO_3, acetone, 70 ^oC; e) 1*H*-1,2,3-triazole, K_2CO_3, acetone, 70 ^oC; f) 1*H*-1,2,4-triazole, K_2CO_3, acetone, 70 ^oC

Figure S2. Synthesis of derivatives bearing the most common zinc-binding groups (part II)

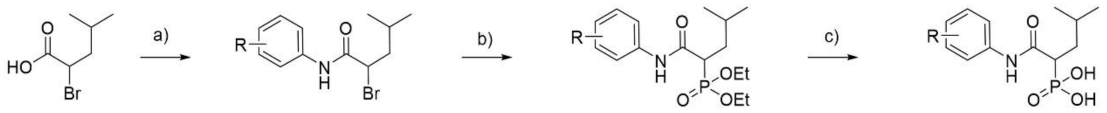

S6			
	**S5a:** Ar=4-Me-Ph (>99%)	**S7a:** Ar=4-Me-Ph (39%)	**4a:** Ar=4-Me-Ph (71%)
	**S5b:** Ar=4-CF_3-Ph (58%)	**S7b:** Ar=4-CF_3-Ph (82%)	**4b:** Ar=4-CF_3-Ph (45%)
	**S5c:** Ar=3,4-diCl-Ph (67%)	**S7c:** Ar=3,4-diCl-Ph (82%)	**4c:** Ar=3,4-diCl-Ph (41%)
	**S5d:** Ar=2-F-4-Me-Ph (76%)	**S7d:** Ar=2-F-4-Me-Ph (75%)	**4d:** Ar=2-F-4-Me-Ph (72%)
	**S5e:** Ar=2,4-diMe-Ph (85%)	**S7e:** Ar=2,4-diMe-Ph (64%)	**4e:** Ar=2,4-diMe-Ph (45%)
	**S5f:** Ar=4-OMe-Ph (98%)	**S7f:** Ar=4-OMe-Ph (38%)	**4f:** Ar=4-OMe-Ph (29%)
	**S5g:** Ar=4-Ac-Ph (53%)	**S7g:** Ar=4-Ac-Ph (61%)	**4g:** Ar=4-Ac-Ph (61%)
	**S5h:** Ar=4-F-Ph (87%)	**S7h:** Ar=4-F-Ph (74%)	**4h:** Ar=4-F-Ph (48%)
	**S5i:** Ar=4-Cl-Ph (62%)	**S7i:** Ar=4-Cl-Ph (71%)	**4i:** Ar=4-Cl-Ph (69%)
	**S5j:** Ar=4-Br-Ph (51%)	**S7j:** Ar=4-Br-Ph (66%)	**4j:** Ar=4-Br-Ph (78%)
	**S5k:** Ar= 4-CN-Ph (60%)	**S7k:** Ar= 4-CN-Ph (65%)	**4k:** Ar= 4-CN-Ph (35%)
	**S5l:** Ar= 4-SO_2CF_3-Ph (37%)	**S7l:** Ar= 4-SO_2CF_3-Ph (66%)	**4l:** Ar= 4-SO_2CF_3-Ph (53%)
	**S5m:** Ar=1-naphtyl (72%)	**S7m:** Ar=1-naphtyl (70%)	**4m:** Ar=1-naphtyl (73%)
	**S5n:** Ar=2-naphtyl (71%)	**S7n:** Ar=2-naphtyl (90%)	**4n:** Ar=2-naphtyl (66%)
	**S5o:** Ar=2,3-dihydro-1*H*-inden-5-yl (86%)	**S7o:** Ar=2,3-dihydro-1*H*-inden-5-yl (80%)	**4o:** Ar=2,3-dihydro-1*H*-inden-5-yl (56%)

a) substituted aniline, EDC-HCl, DCM, r.t.; b) $P(OEt)_3$, 150 ^oC; c) 1. TMSBr, DCM, Ar, r.t.; 2) MeOH

Figure S3. Synthesis of α-isobutyl phosphonic acid derivatives.

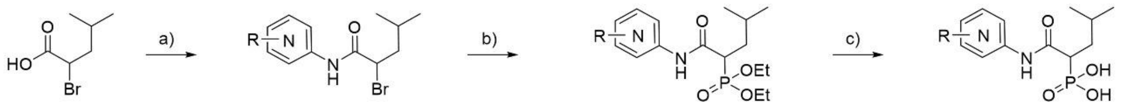

S6

S5p: Ar= 5-chloropyridin-2-yl (30%)
S5q: Ar= 6-chloropyridin-3-yl (63%)
S5r: Ar= 6-carboxamidepyridin-3-yl (20%)
S5s: Ar= 6-carboxymethylpyridin-3-yl (23%)
S5t: Ar= 2-chloropyrimidin-5-yl (63%)
S5u: Ar= 6-chloropyridazin-3-yl (20%)

S7p: Ar= 5-chloropyridin-2-yl (55%)
S7q: Ar= 6-chloropyridin-3-yl (49%)
S7r: Ar= 6-carboxamidepyridin-3-yl (41%)
S7s: Ar= 6-carboxymethylpyridin-3-yl (49%)
S7t: Ar= 2-chloropyrimidin-5-yl (72%)
S7u: Ar= 6-chloropyridazin-3-yl (57%)

4p: Ar= 5-chloropyridin-2-yl (45%)
4q: Ar= 6-chloropyridin-3-yl (52%)
4r: Ar= 6-carboxamidepyridin-3-yl (80%)
4s: Ar= 6-carboxymethylpyridin-3-yl (83%)
4t: Ar= 2-chloropyrimidin-5-yl (43%)
4u: Ar= 2-bromopyrimidin-5-yl (19%)
4v: Ar= 6-chloropyridazin-3-yl (35%)
4w: Ar= 6-bromopyridazin-3-yl (18%)

a) substituted aniline, EDC-HCl, DCM, r.t. or $SOCl_2$, THF, cat. DMF, 50 ^oC; b) $P(OEt)_3$, 150 ^oC; c) 1. TMSBr, DCM, Ar, r.t.; 2) MeOH

Figure S4. Synthesis of α-isobutyl phosphonic acid derivatives with 6-membered ring nitrogen heterocycles.

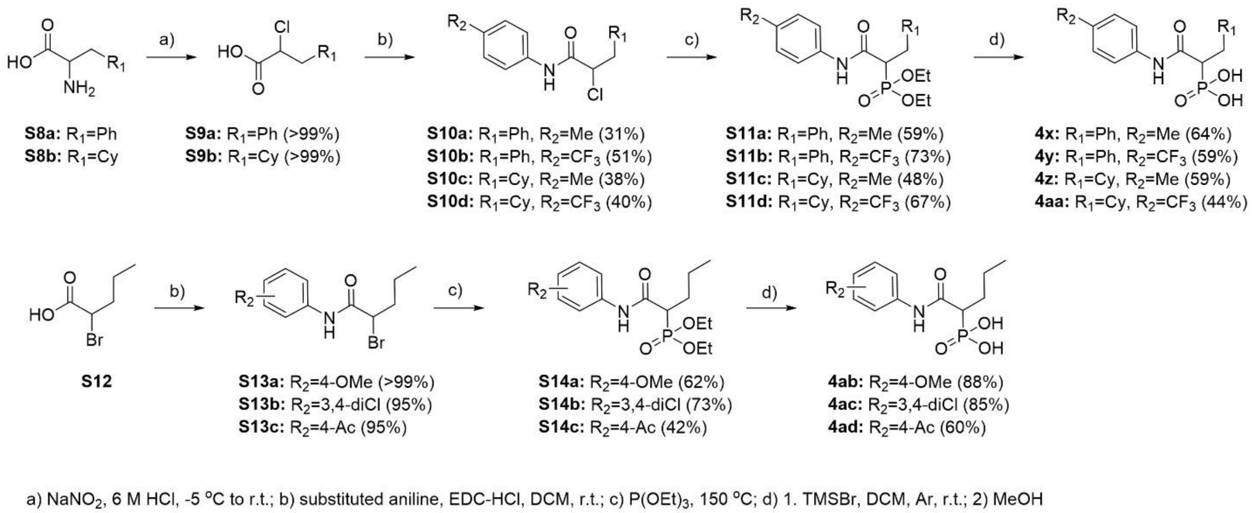

Figure S5. Synthesis of α-benzyl, α-cyclohexylmethyl and α-propyl phosphonic acid derivatives.

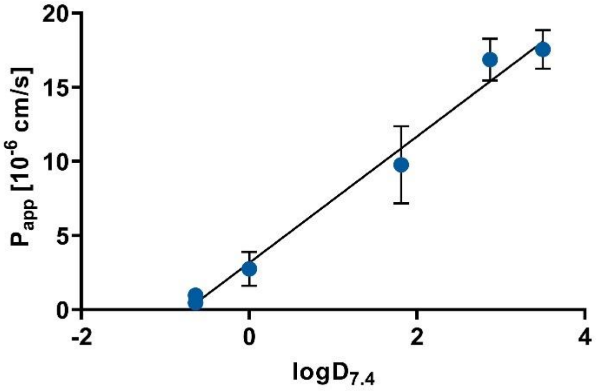

Figure S6. Correlation of permeability P_{app} and $logD_{7.4}$ for the selected ZBGs. Linear regression yielded $R^2 = 0.9865$.

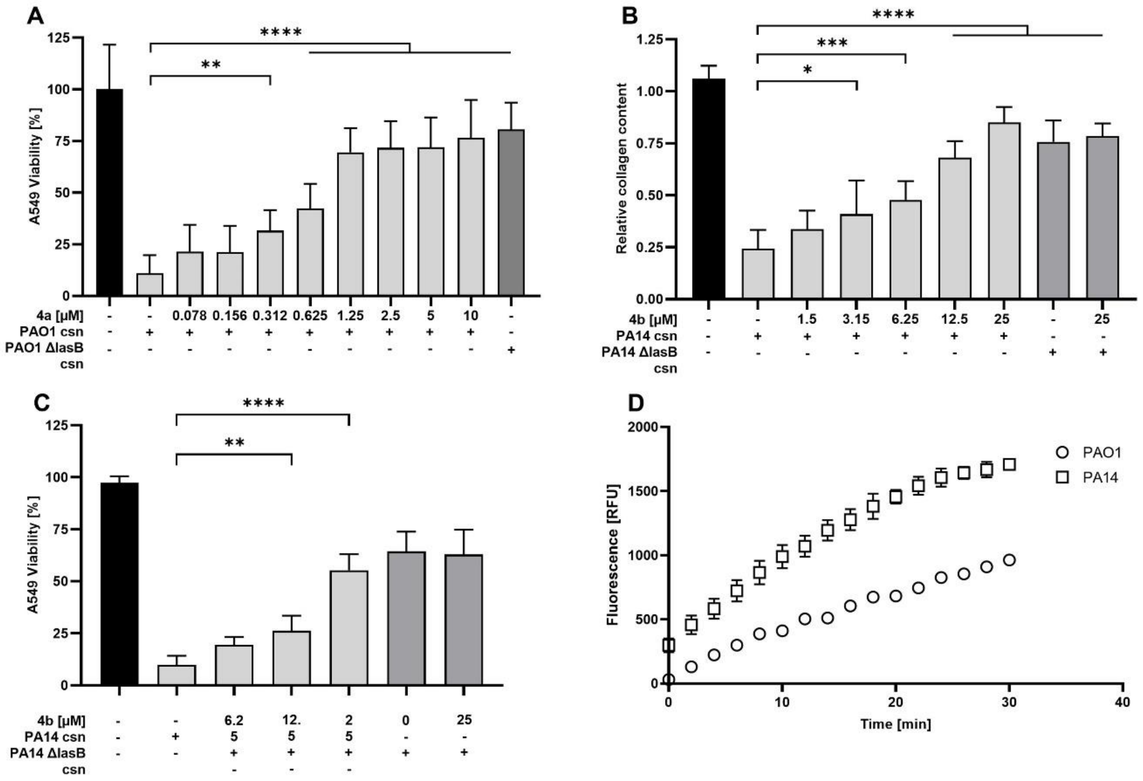

Figure S7. The effect of **4a** and **4b** on lung cells (A549) challenged with *P. aeruginosa* PAO1 or PA14 csn. (**A**) The dose-response inhibitory effect of **4a** against 10% (v/v) *P. aeruginosa* PAO1 csn (culture supernatant). (**B**) **4b** maintained the lung cells upon treatment with 15% (v/v) of wt PA14 csn. (**C**) **4b** maintained the collagen content of lung cells upon treatment with 15% (v/v) of wt PA14 csn or PA14 Δ*lasB* csn. Relative collagen content was calculated by dividing collagen amounts of treated conditions with csn and compound by collagen of the untreated healthy condition. Each graph represents three independent experiments ± SD. Statistical analysis was performed with one-way ANOVA and statistical significance was analyzed by Tukey test. Significance was calculated by comparing non-treated vs treated cells with **4a** or **4b** (mean ± SD, **** $p \leq 0.0001$, *** $p \leq 0.001$, * $p \leq 0.05$, ns non-significant) wt PA14: wild-type PA14, PA14 Δ*lasB*: LasB knockout PA14, csn: culture supernatant. (**D**) Comparing the activity of LasB in culture supernatants of PAO1 and PA14 using a FRET-based proteolytic assay,[30] based on the ability of LasB to cleave a synthetic quenched substrate introduced by Nishino and Powers:[35] Both strains exhibited significant LasB activity; however, PA14 demonstrated a considerably higher signal than PAO1.

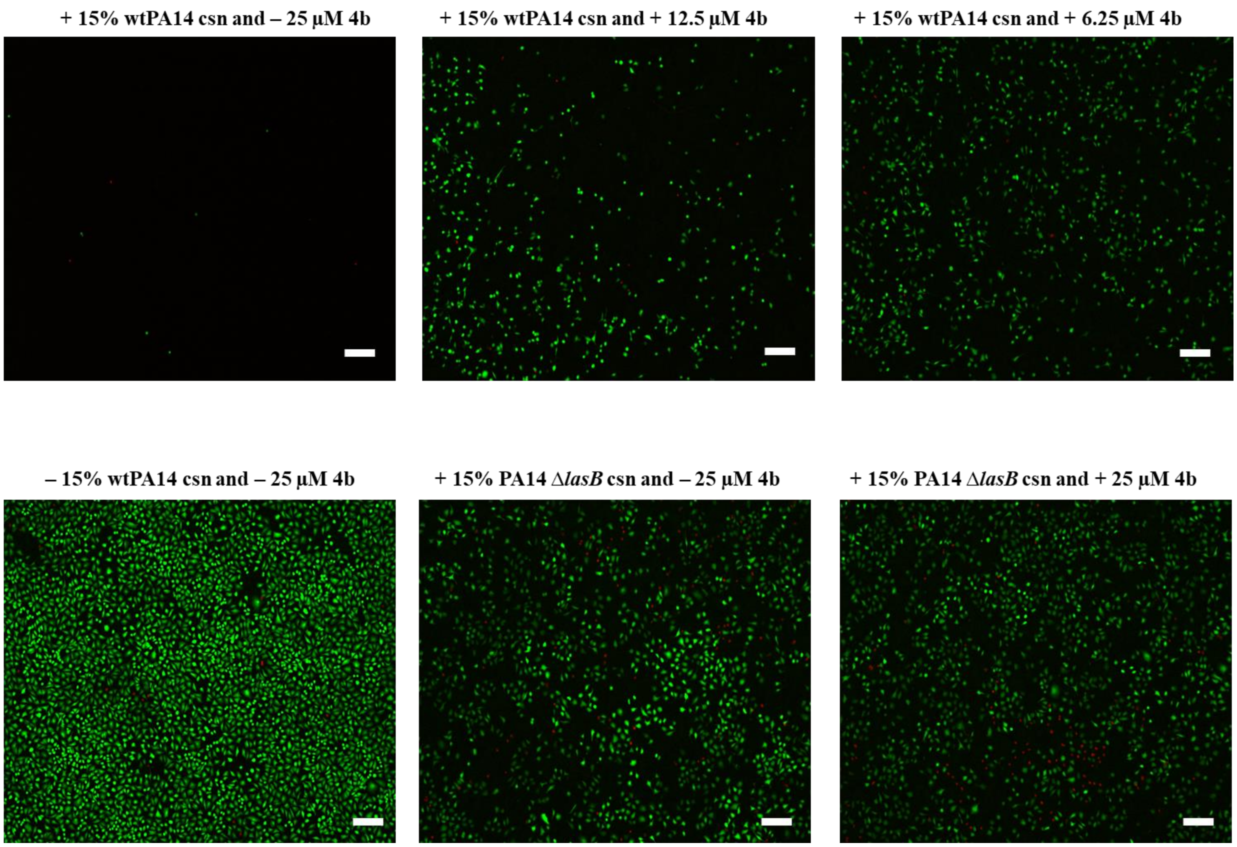

Figure S8. 4b maintained the viability of lung (A549) cells upon the treatment with wt 15% PA14. Live/dead imaging with A549 cells challenged with 15% (v/v) of PA14 or PA14 Δ*lasB* csn with and without **4b**. Green signals: living cells and red signals: dead cells, red signals in some cases were lost because the detached cells were washed away after the rinsing step with PBS. wt PA14: wild-type PA14, PA14 Δ*lasB*: LasB knockout PA14, csn: culture supernatant. Scale bar: 200 µm for images.

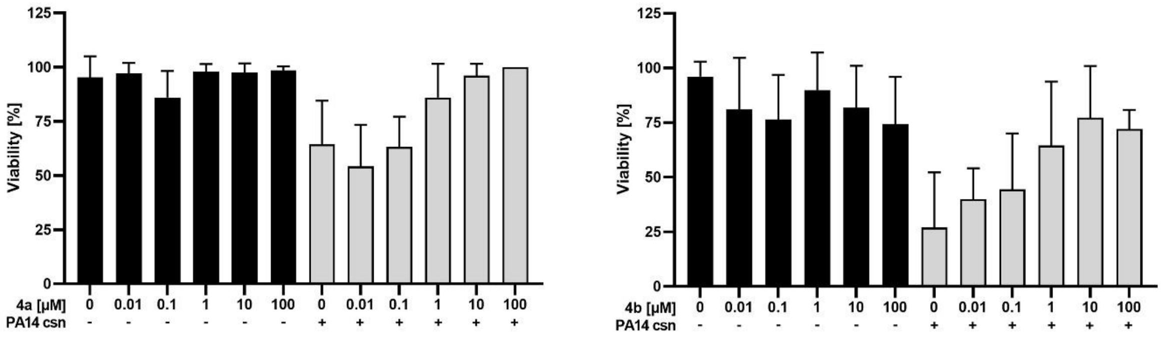

Figure S9. The effect of **4a** and **4b** on the viability of 3D lung organoids and the rescue effect by LasB inhibitors. Statistical analysis was performed using One-way ANOVA followed by Dunnett's multiple comparisons test, comparing the mean value of each concentration to the mean value of csn-added without any treatment with compounds (**** $p \leq 0.0001$, *** $p \leq 0.001$, ** $p \leq 0.01$, * $p \leq 0.05$).

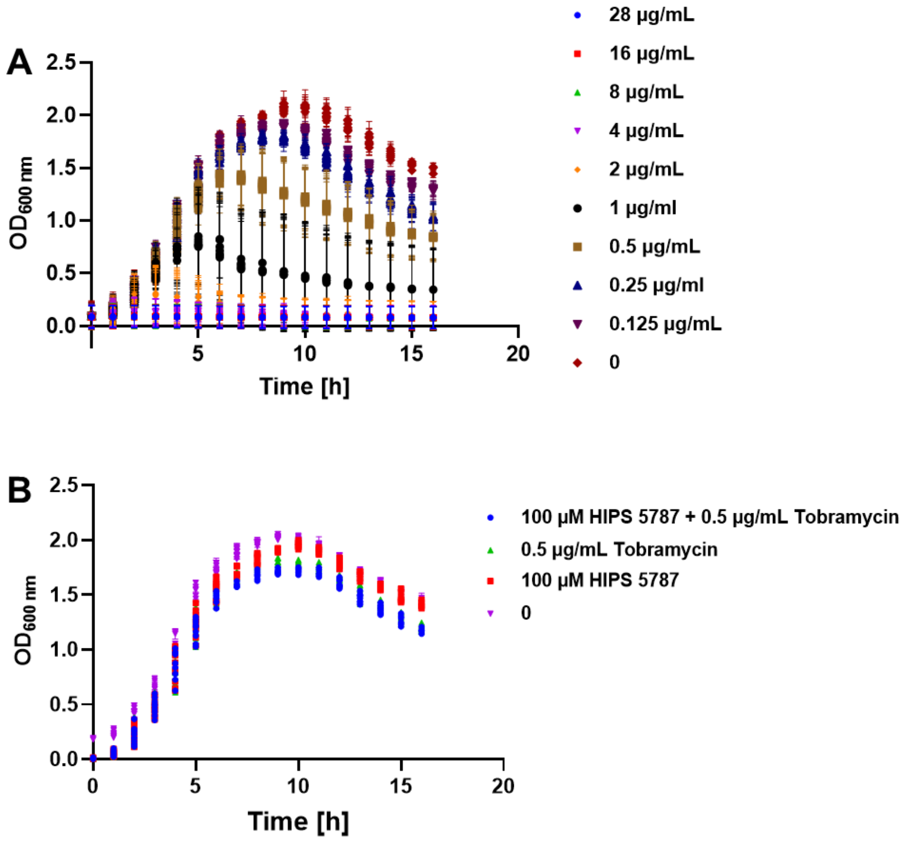

C

Compound	Percent (%) growth inhibition of *P. aeruginosa* PA14 at 100 µM compound concentration
**4a**	**<10**
**4b**	**<10**
**4k**	**<10**
**4l**	**<10**

Figure S10. The growth curves of PA14 treated with (**A**) tobramycin (0–28 µg/mL) and (**B**) 100 µM **4b** + 0.5 µg/mL tobramycin. Each experiment was performed at least three times. (**C**) Antibacterial activity against *P. aeruginosa* PA14.

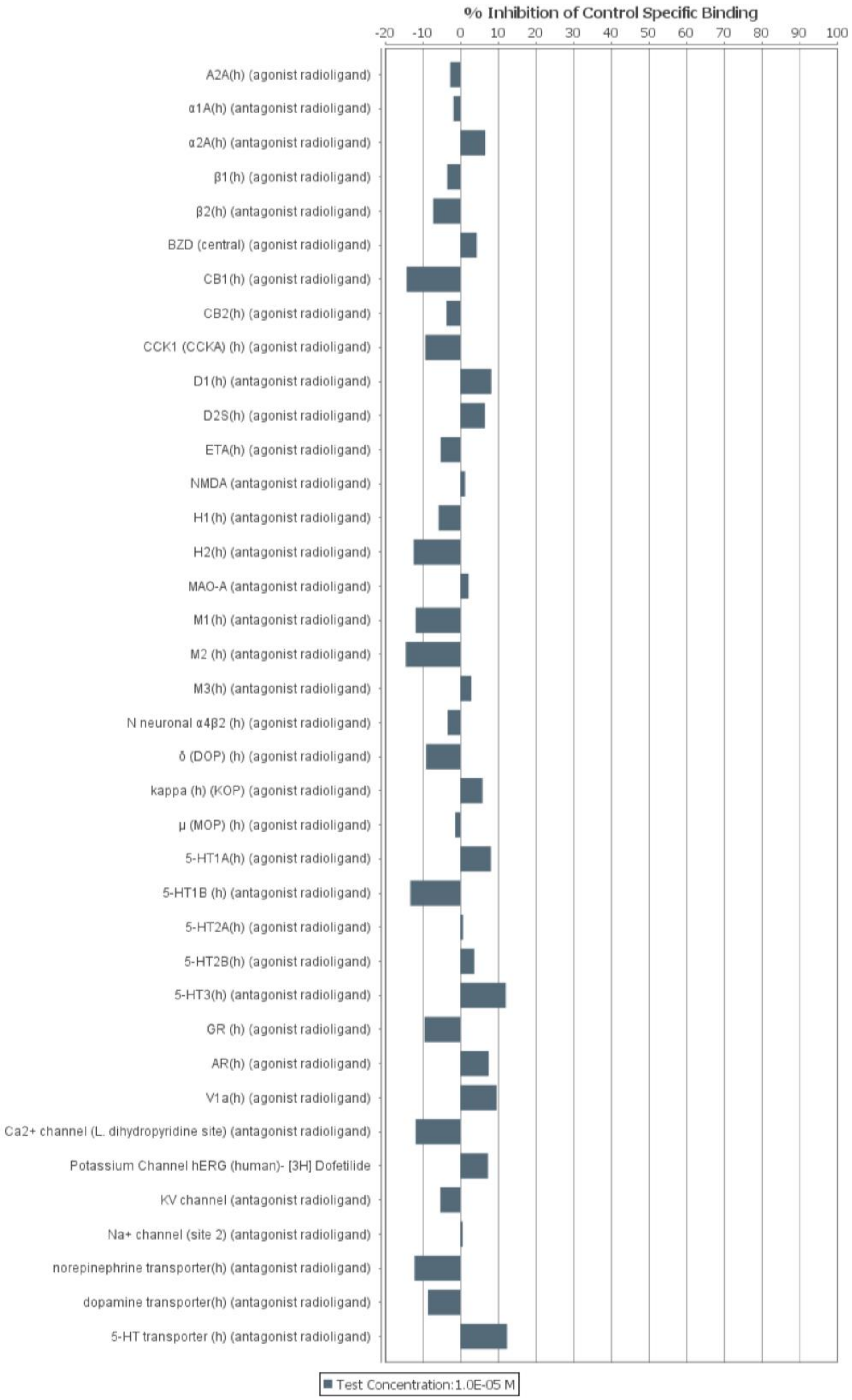

Figure S11. Safety screen 44 for compound **4a**.

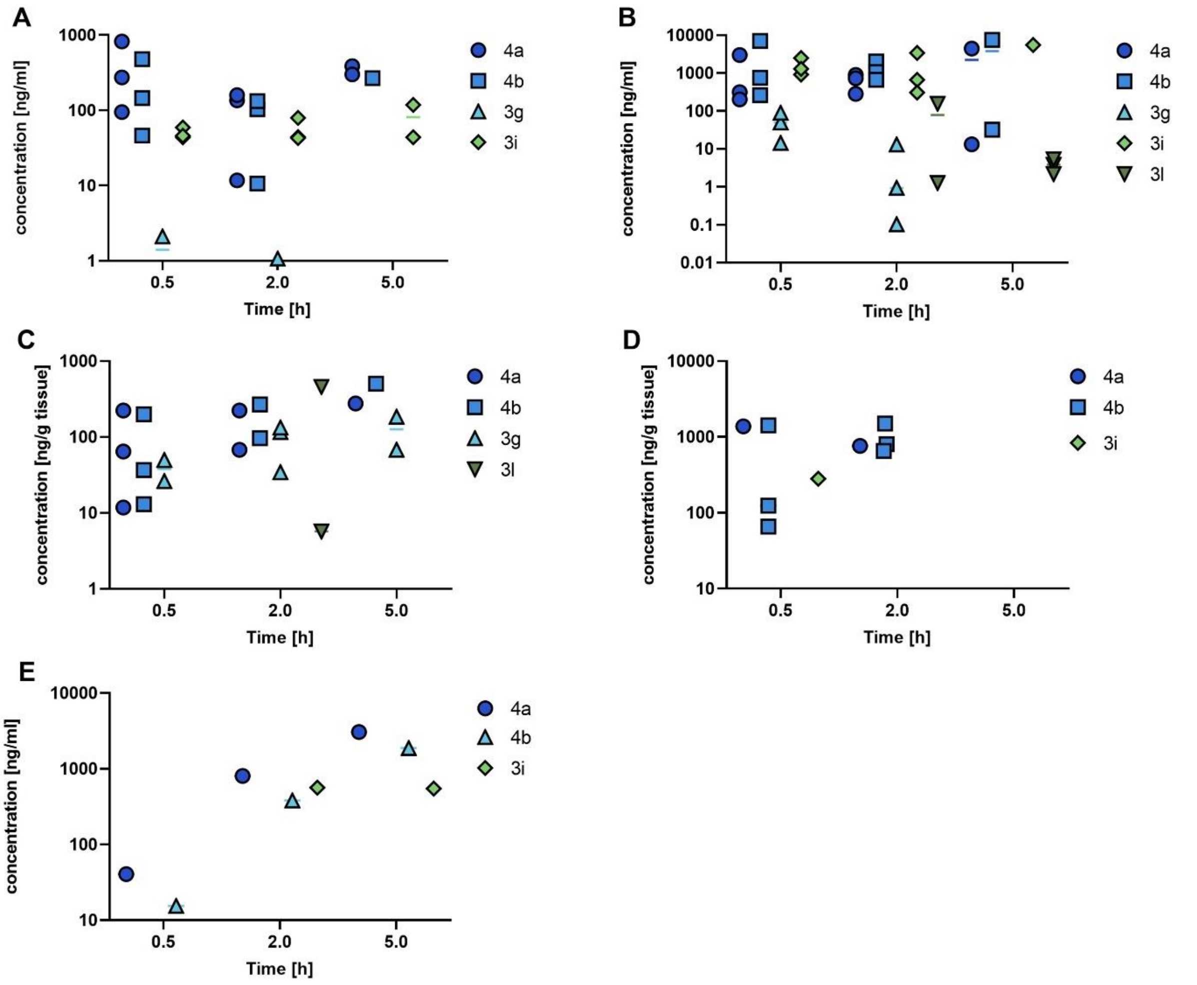

Figure S12. Concentration levels of the five selected compounds in (**A**) plasma, (**B**) BALF, (**C**) liver tissue, (**D**) kidney and (**E**) urine after IT administration at 0.25 mg/kg (cassette dosing).

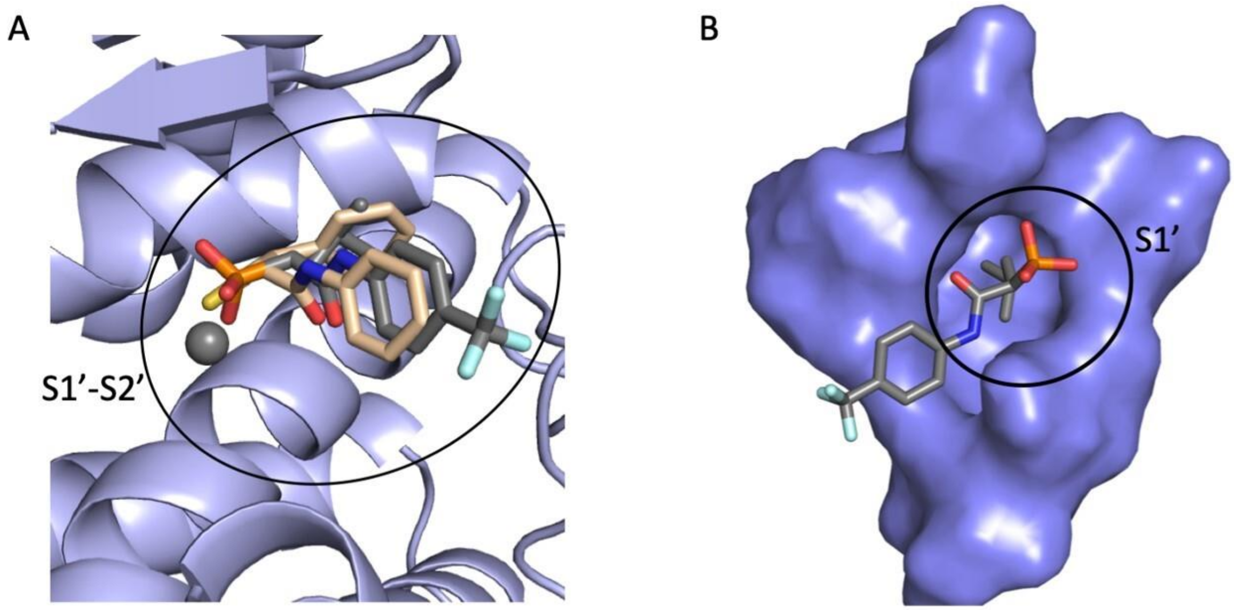

Figure S13. (**A**) Superposition of LasB (slate) structures in complex with **4b** (gray) or mercaptoacetamide derivative (wheat; major conformation shown; PDB code: 7OC7) showing S1'-S2' binding sites of the enzyme. The active-site Zn^{2+} cation is shown as gray sphere. (**B**) Surface representation of LasB substrate binding pocket highlighting the S1' pocket occupied by the *i*Bu group of **4b**.

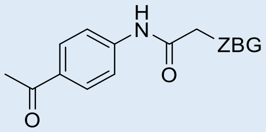

Cpd.	ZBG	% LasB inhibition @600 µM	Cpd.	ZBG	% inhibition LasB @600 µM
**S15a**	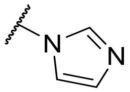	n.i.	**S15g**	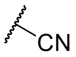	n.i.
**S15b**	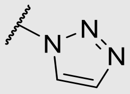	n.i.	**S15h**	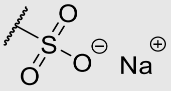	n.i.
**S15c**	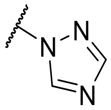	n.i.	**2**	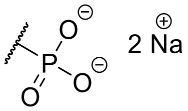	33 ± 4
**S15d**	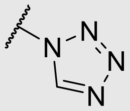	n.i.	**S15i**	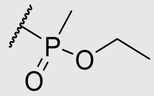	n.i.
**S15e**	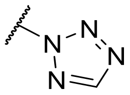	n.i.	**S15j**	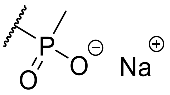	n.i.
**S15f**	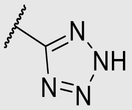	n.i.			

[a]Means and SD of at least two independent experiments. n.i. = inhibition <10%.

Table S1. Our previously published ColH inhibitors[34] bearing various ZBGs: In this work evaluated for their activities towards LasB[a]

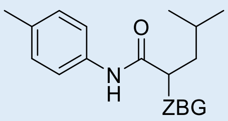

Cpd.	ZBG	% inh LasB @100 µM or IC_{50} (µM)	Cpd.	ZBG	% inh LasB @100 µM or IC_{50} (µM)
**1**[29]	SH	IC_{50} = 0.40 ± 0.13	**3h**	CN	n.i.
**3a**	NHBoc	n.i.	**3i**	SO_3H	IC_{50} = 16 ± 0
**3b**	NH_2	n.i.	**3j**	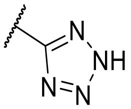	n.i.
**3c**	OSO_2NH_2	IC_{50} = 53 ± 4	**3k**	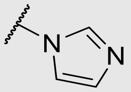	14 ± 4
**3d**	CO_2Et	n.i.	**3l**	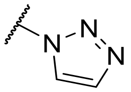	IC_{50} = 2.8 ± 0.1
**3e**	COOH	46 ± 3	**3m**	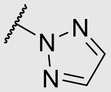	IC_{50} = 5.3 ± 0.2
**3f**	$CONH_2$	n.i.	**3n**	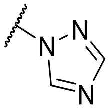	n.i.
**3g**	CONHOH	IC_{50} = 0.014 ± 0.001	**4a**	PO_3H_2	IC_{50} = 0.051 ± 0.007

[a]Means and SD of at least two independent experiments. n.i. = <10% inhibition.

Table S2. 4-Methyl-*N*-(*p*-tolyl)pentanamides bearing various ZBGs in alpha-position and their inhibition of LasB[a]

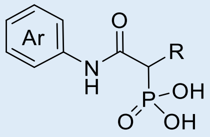

Cpd.	Ar	R	IC_{50} (nM)	Cpd.	Ar	R	IC_{50} (nM)
**4a**	4-Me-Ph	*i*Bu	51.2 ± 6.7 (123 ± 56[b])	**4l**	4-SO_2CF_3-Ph	*i*Bu	27.7 ± 5.5
**4b**	4-CF_3-Ph	*i*Bu	26.3 ± 7.6 (76 ± 23[b])	**4m**	1-naphtyl	*i*Bu	69.1 ± 7.5
**4c**	3,4-diCl-Ph	*i*Bu	25.7 ± 4.1	**4n**	2-naphtyl	*i*Bu	63.6 ± 9.9
**4d**	2-F-4-Me-Ph	*i*Bu	116.1 ± 16.1	**4o**	2,3-dihydro-1*H*-inden-5-yl	*i*Bu	44.3 ± 4.5
**4e**	2,4-diMe-Ph	*i*Bu	84.5 ± 2.7	**4x**	4-Me-Ph	Bn	137.4 ± 33.0
**4f**	4-OMe-Ph	*i*Bu	51.8 ± 10.4	**4y**	4-CF_3-Ph	Bn	101.5 ± 0.9
**4g**	4-Ac-Ph	*i*Bu	40.4 ± 11.6	**4z**	4-Me-Ph	CH_2Cy	2,029 ± 115
**4h**	4-F-Ph	*i*Bu	92.7 ± 21.6	**4aa**	4-CF_3-Ph	CH_2Cy	1,872 ± 25
**4i**	4-Cl-Ph	*i*Bu	47.6 ± 4.4	**4ab**	4-OMe-Ph	Pr	1,110 ± 20
**4j**	4-Br-Ph	*i*Bu	48.6 ± 2.3	**4ac**	3,4-diCl-Ph	Pr	2,050 ± 270
**4k**	4-CN-Ph	*i*Bu	25.0 ± 1.3 (85 ± 0[b])	**4ad**	4-Ac-Ph	Pr	870 ± 30

[a]Means and SD of at least two independent experiments. [b]IC_{50} in presence of 1% pulmonary surfactant

Table S3. LasB inhibition by α-isobutyl, α-benzyl, α-cyclohexylmethyl and α-propyl phosphonic acid derivatives[a]

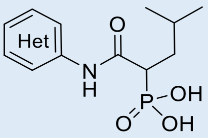

Cpd.	Ar	IC_{50} (μM)	Cpd.	Ar	IC_{50} (μM)
**4p**	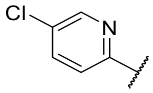	0.50 ± 0.04	**4t**	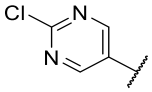	0.50 ± 0.02
**4q**	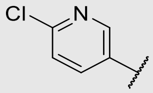	1.13 ± 0.02	**4u**	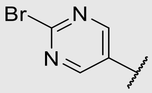	0.38 ± 0.00
**4r**	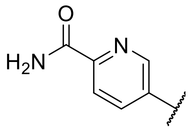	0.22 ± 0.02	**4v**	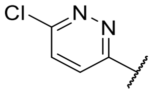	18.80 ± 0.74
**4s**	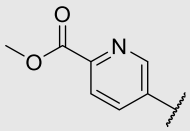	0.19 ± 0.01	**4w**	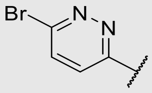	7.91 ± 0.39

[a]Means and SD of at least two independent experiments.

Table S4. LasB inhibition by α-isobutyl phosphonic acid derivatives with 6-membered ring nitrogen heterocycles[a]

Compound	Microsomal Cl_{int} [µl/mg/min]			Plasma $T_{1/2}$ [min]		
	Mouse (C57BL6)	Rat (Wistar)	Minipig (Göttingen)	Mouse (C57BL6)	Rat (Wistar)	Minipig (Göttingen)
**4a**	<11.6	<11.6	<11.6	>240	>240	>240
**4b**	<11.6	<11.6	<11.6	>240	>240	>240
**4h**	<11.6	<11.6	<11.6	>240	>240	>240

Table S5. Species profiling of 3 selected phosphonates. Cl_{int} = intrinsic clearance, $T_{1/2}$ = half-life.

Cpd.	Class	% growth inhibition after 48h at 100 µM HepG2	HEK293
**3g**	hydroxamic acid	26 ± 11	12 ± 6
**3i**	sulfonic acid	n.i.	n.i.
**3l**	1*H*-1,2,3-triazole	n.i.	n.i.
**3m**	2*H*-1,2,3-triazole	n.i.	21 ± 1
**4a**	phosphonic acid	n.i.	n.i.
**4b**		18 ± 11	11 ± 1
**4k**		n.i.	15 ± 23
**4l**		12 ± 6	23 ± 0

[a]Means and SD of at least two independent experiments. n.i= <10 % inhibition

Table S6. Cytotoxicity of selected compounds bearing different ZBGs against HepG2 and HEK293[a]

		% inhibition @ 100 µM						
**Cpd.**	**Class**	**MMP-1**	**MMP-2**	**MMP-3**	**HDAC-3**	**HDAC-8**	**TACE**	**COX-1**
**3g**	hydroxamic acid	29 ± 3	26 ± 2	16 ± 7	n.i.	n.i.	16 ± 2 µM[b]	n.i.
**3i**	sulfonic acid	12 ± 2	n.i.	n.i.	n.i.	n.i.	n.i.	n.i.
**3l**	1*H*-1,2,3-triazole	n.i.	n.i.	n.i.	n.i.	n.i.	n.i.	n.i.
**3m**	2*H*-1,2,3-triazole	n.i.	n.i.	n.i.	n.i.	n.i.	n.i.	n.i.
**4a**	phosphonic acid	n.i.	n.i.	n.i.	n.i.	n.i.	n.i.	n.i.
**4b**		21 ± 1	25 ± 4	29 ± 8	n.d.	n.d.	n.i.	n.i.
**4k**		10 ± 5	n.i.	11 ± 5	n.d.	n.d.	n.i.	n.i.
**4l**		n.i.	n.i.	n.i.	n.d.	n.d.	n.i.	n.i.

[a]Means and SD of at least two independent experiments. [b]IC_{50} value. n.i. = <10% inhibition; n.d. = not determined.

Table S7. Inhibition of three MMPs, two HDACs, TACE and COX-1 in presence of selected compounds bearing different ZBGs[a]

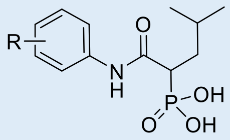

Cpd.	R	% inhibition ColH-PD @1 μM
**4a**	4-Me	n.i.
**4b**	4-CF_3	14 ± 8
**4c**	3,4-diCl	n.i.
**4d**	2-F-4-Me	n.i.
**4f**	4-OMe	n.i.
**4g**	4-Ac	65 ± 5

[a]Means and SD of at least two independent experiments;
n.i. = <10% inhibition

Table S8. ColH-PD inhibition by selected α-isobutyl phosphonic acid derivatives[a]

Compound	Conc. (µM)	Screening observations				Survival rate (%)
		2 dpf	3 dpf	4 dpf	5 dpf	
**4a**	100	-	-	-	3 nsb	100
	50	-	1 edema	1 edema	1 nsb, 1 edema	100
	25	-	-	-	-	100
	12.5	-	1 edema	1 edema	1 nsb, 1 edema	100
**4b**	100	-	1 edema	1 edema	1 nsb, 1 edema	100
	50	-	-	-	2 nsb	100
	25	-	-	-	1 nsb	100
	12.5	-	-	-	-	100
**DMSO**	1%	-	-	-	3 nsb	100
**Danieau's**	-	-	2 edema	2 edema	2 edema	100

Table S9. Zebrafish embryo toxicity of compounds **4a** and **4b**. Dpf: days post fertilization.

	**4a**	**4b**
C_{max} [ng/mL] Plasma	396	266
AUC 0-t Plasma [ng/ml*h]	1,139	806
AUC BALF [ng/ml*h]	5,959	11,177
AUC ELF [µg/ml*h]	42.8	79.4
Ratio AUC ELF/plasma	37.5	98.5

Table S10. PK parameters for **4a** and **4b** after IT administration at 0.25 mg/kg (cassette).

	**4b**
C_{max} [µg/mL] ELF	19.9 ± 14.0
T_{max} [h] ELF	0.58 ± 0.4
AUC 0-t [µg/mL*h] ELF	49.7 ± 16.2
ELF/plasma ratio	6.12
C_{max} [ng/g] lung	78.4 ± 15.9
T_{max} [h] lung	0.33 ± 0.1
AUC 0-t [ng/g*h] lung	249.0 ± 72.9

Table S11. PK parameters of **4b** in ELF and lung tissue after nebulization at 10 mg/kg.

	**4b**
$t_{1/2}$ [h]	4.0 ± 0.3
C_{max} [ng/mL]	633 ± 59
T_{max} [h]	4.0 ± 3.5
AUC 0-t [ng/mL*h]	8,133 ± 588
MRT [h]	6.8 ± 0.3
Vz/F_obs [l/kg]	7.1 ± 0.1
Cl/F_obs [mL/min/kg]	20.7 ± 2.0
F [%]	-

Table S12. PK parameters of **4b** in plasma after nebulization at 10 mg/kg.

LasB_4b	
**PDB ID**	8CC4
**Data collection**	
Space group	P 2 2_1 2_1
Cell dimension	
a, *b*, *c* (Å)	44.4, 77.1, 152.2
α, β, γ (°)	90.0, 90.0, 90.0
Wavelength (Å)	1.0332
Resolution	2.70 (2.83 – 2.70) *
R_{sym} or R_{merge}	0.190 (0.598)
R_{pim}	0.075 (0.242)
CC (1/2)	0.990 (0.864)
I / σI	6.8 (2.4)
Completeness (%)	96.4 (97.6)
Redundancy	5.9 (5.7)
**Refinement**	
Resolution (Å)	44.40 – 2.70
No. reflection	14190
R_{work} / R_{free}	0.206 / 0.260
No. atoms	4,731
Protein	4,629
Ligands	48
Solvent	54
Protein residues	596
B-factors	37.83
Protein	37.94
Ligands	34.04
Water	31.84
**R. m. s deviations**	
Bond length (Å)	0.004
Bond angels (°)	0.66
MolProbity score	1.81

*Values in parentheses are for highest-resolution shell

Table S13. Data collection and refinement statistics for **LasB_4b**.

REFERENCES

[50] F-M. Klingler, T. A. Wichelhaus, D. Frank, J. Cuesta-Bernal, J. El-Delik, H. F. Müller, H. Sjuts, S. Göttig, A. Koenigs, K. M. Pos, D. Pogoryelov, E. Proschak, Approved Drugs Containing Thiols as Inhibitors of Metallo-β-lactamases: Strategy To Combat Multidrug-Resistant Bacteria. *J. Med. Chem.* **58**, 3626–3630 (2015).

[51] J. Haupenthal, C. Baehr, S. Zeuzem, A. Piiper, RNAse A-like enzymes in serum inhibit the anti-neoplastic activity of siRNA targeting polo-like kinase 1. *Int. J. Cancer* **121**, 206−210 (2007).

[52] U. Eckhard, E. Schönauer, H. Brandstetter, Structural Basis for Activity Regulation and Substrate Preference of Clostridial Collagenases G, H, and T. *J. Biol. Chem.* **288**, 20184–20194 (2013).

[53] E. Schönauer, A. M. Kany, J. Haupenthal, K. Hüsecken, I. J. Hoppe, K. Voos, S. Yahiaoui, B. Elsässer, C. Ducho, H. Brandstetter, R. W. Hartmann, Discovery of a Potent Inhibitor Class with High Selectivity toward Clostridial Collagenases. *J. Am. Chem. Soc.* **139**, 12696–12703 (2017).

[54] R. Bals, C. Beisswenger, S. Blouquit, T. Chinet, Isolation and air–liquid interface culture of human large airway and bronchiolar epithelial cells. *J Cyst Fibros.* **3** (suppl 2), 49–51 (2004).

[55] J. Maes, L. Verlooy, O. E. Buenafe, P. A. M. de Witte, C. V. Esguerra, A. D. Crawford, Evaluation of 14 Organic Solvents and Carriers for Screening Applications in Zebrafish Embryos and Larvae. *PLoS One* **7**, e43850 (2012).

[56] Y. Zhang, M. Huo, J. Zhou, S. Xie, PKSolver: An add-in program for pharmacokinetic and pharmacodynamic data analysis in Microsoft Excel. *Comput. Methods Programs Biomed.* **99**, 306–314 (2010).

[57] S. Kiem, J. J. Schentag, Interpretation of antibiotic concentration ratios measured in epithelial lining fluid. *Antimicrob. Agents Chemother*. **52**, 24–36 (2008).

[58] W. Elgaher, M. Fruth, M. Groh, J. Haupenthal, R. W. Hartmann, Expanding the scaffold for bacterial RNA polymerase inhibitors: design, synthesis and structure–activity relationships of ureido-heterocyclic-carboxylic acids. *RSC Adv.* **4**, 2177–2194 (2014).

[59] A. Burkhardt, T. Pakendorf, B. Reime, J. Meyer, P. Fischer, N. Stübe, S. Panneerselvam, O. Lorbeer, K. Stachnik, M. Warmer, P. Rödig, D. Göries, A. Meents, Status of the crystallography beamlines at PETRA III. *Eur. Phys. J. Plus* **131**, 56 (2016).

[60] G. Winter, Xia2: An Expert System for Macromolecular Crystallography Data Reduction. *J. Appl. Crystallogr.* **43**, 186–190 (2010).

[61] W. Kabsch, XDS. *Acta Crystallogr. Sect. D Biol. Crystallogr.* **66**, 125–132 (2010).

[62] A. J. McCoy, R. W. Grosse-Kunstleve, P. D. Adams, M. D. Winn, L. C. Storoni, R. J. Read, Phaser Crystallographic Software. *J. Appl. Crystallogr.* **40**, 658–674 (2007).

[63] P. Emsley, B. Lohkamp, W. G. Scott, K. Cowtan, Features and Development of Coot. *Acta Crystallogr. D. Biol. Crystallogr.* **66**, 486–501 (2010).

[64] P. D. Adams, P. V. Afonine, G. Bunkóczi, V. B. Chen, I. W. Davis, N. Echols, J. J. Headd, L-W. Hung, G. J. Kapral, R. W. Grosse-Kunstleve, A. J. McCoy, N. W. Moriarty, R. Oeffner, R. J. Read, D. C. Richardson, J. S. Richardson, T. C. Terwilliger, P. H. Zwart, PHENIX: A Comprehensive Python-Based System for Macromolecular Structure Solution. *Acta Crystallogr. D. Biol. Crystallogr.* **66**, 213–221 (2010).

[65] P. Skubák, G. N. Murshudov, N. S. Pannu, Direct Incorporation of Experimental Phase Information in Model Refinement. *Acta Crystallogr. Sect. D* **60**, 2196–2201 (2004).

[66] C. M. Adeyemi, M. Isaacs, D. Mnkandhla, R. Klein, H. C. Hoppe, R. W. M. Krause, K. A. Lobb, P. T. Kaye, Synthesis and anti-parasitic activity of C-benzylated (N-arylcarbamoyl)alkylphosphonate esters. *Tetrahedron* **73**, 1661–1667 (2017).

[67] Y. Zhu, A. Chidekel, T. H. Shaffer, Cultured Human Airway Epithelial Cells (Calu-3): A Model of Human Respiratory Function, Structure, and Inflammatory Responses. *Crit. Care Res. Pract.* **2010**, 394578 (2010).

[68] H. X. Ong, D. Traini, P. M. Young, Pharmaceutical applications of the Calu-3 lung epithelia cell line. *Expert Opin. Drug Deliv.* **10**, 1287–1302 (2013).

[69] H. Laronha, J. Caldeira, Structure and Function of Human Matrix Metalloproteinases. *Cells* **9**, 1076 (2020).

[70] S. Löffek, O. Schilling, C-W. Franzke, Biological role of matrix metalloproteinases: a critical balance. *Eur. Respir. J.* **38**, 191–208 (2011).

[71] M. Haberland, R. L. Montgomery, E. N. Olson, The many roles of histone deacetylases in development and physiology: implications for disease and therapy. *Nat Rev Genet.* **10**, 32–42 (2009).

[72] K. Gudis, C. Sakamoto, The Role of Cyclooxygenase in Gastric Mucosal Protection. *Dig Dis Sci* **50**, 16–23 (2005).

[73] A. J. Hill, H. Teraoka, W. Heideman, R. E. Peterson, Zebrafish as a Model Vertebrate for Investigating Chemical Toxicity. *Toxicol. Sci.* **86**, 6–19 (2005).

[74] J. R. Goldsmith, C. Jobin, Think Small: Zebrafish as a Model System of Human Pathology. *J Biomed Biotechnol.* **2012**, 817341 (2012).
